# Effectiveness, Safety, and Workflow Burden of Large Language Model–Based Medical Report Generation: Systematic Review

**DOI:** 10.2196/97007

**Published:** 2026-09-09

**Authors:** Jie-Lin Huang, Ji-Qing Zhu, Xiao-Guang Ni

**Affiliations:** 1 Department of Endoscopy, National Cancer Center/National Clinical Research Center for Cancer/Cancer Hospital, Chinese Academy of Medical Sciences and Peking Union Medical College, No. 17 Panjiayuan South Lane, Chaoyang District, Beijing, 100021, China, +86 10 8778 7606

**Keywords:** large language models, generative AI, medical report generation, radiology, pathology, endoscopy, workflow, systematic review

## Abstract

**Background:**

Systems based on large language models (LLMs), multimodal LLMs, and vision-language foundation models are increasingly being evaluated for medical report generation in imaging and related clinical workflows. Existing reviews have summarized technical architectures, radiology applications, readability, and benchmark performance, but clinical readiness remains uncertain because safety, human oversight, and workflow outcomes are sparsely and inconsistently reported.

**Objective:**

The aim of this study is to assess the effectiveness (expert acceptance and blinded preference), safety (clinically significant, omission, and commission errors), and workflow burden (reporting time, corrections, edit distance, and editing burden) of LLM-based medical report generation.

**Methods:**

We searched PubMed/MEDLINE, Embase, Web of Science Core Collection, Scopus, and the Cochrane Library for studies published from January 1, 2016, through May 15, 2026. Eligible studies evaluated LLMs, multimodal LLMs, or vision-language foundation models for image-to-report generation, impression generation from findings, report drafting, or structured reporting in imaging workflows. Two reviewers performed screening, extraction, risk-of-bias assessment, and Grading of Recommendations Assessment, Development, and Evaluation–informed narrative certainty assessment. Outcomes were clinically significant error rate, omission error rate, commission error rate, reporting time, edit burden, expert acceptance, and blinded expert preference. Meta-analysis was not performed because no comparable outcome had at least 2 studies with compatible task structure and analyzable data.

**Results:**

A total of 101 studies were included. Chest x-ray was the largest modality group (36 studies), followed by computed tomography, magnetic resonance imaging (MRI), ultrasound, endoscopy, pathology, ophthalmic, electrocardiographic, dental, and mixed-modality contexts. No study was judged at low risk of bias; 15 were moderate, 72 high, and 14 serious. Safety and workflow evidence remained heterogeneous and largely nonpoolable. In a chest x-ray study, AI report acceptance was similar to that of radiologist reports (6047/8580, 70.5% vs 6288/8580, 73.3%), but false-negative findings were slightly higher (1584/8580, 18.5% vs 1527/8580, 17.8%). In a clinician-collaboration chest x-ray study, AI reports were equivalent or preferred in 233 of 300 (77.7%) and 170 of 303 (56.1%) cases across 2 datasets; yet, clinically significant errors persisted. In a brain MRI study, AI assistance reduced reading time from 61 to 53 seconds, whereas impression drafting increased editing time and edit distance.

**Conclusions:**

This review shifts the synthesis from plausible report generation to clinically interpretable effectiveness, safety, and workflow effects. Expert acceptance and preference suggested assistive value in selected supervised settings, but these signals were limited by inconsistent reporting of clinically significant errors, omissions, commissions, and failed generations. Workflow effects were mixed, with some studies reporting shorter reading time or drafting support, and others reporting greater editing time or edit distance. The evidence remains too heterogeneous, biased, and sparse on case-level end points to support a pooled meta-analysis or autonomous clinical-readiness claims. Adoption should remain locally validated, clinician-supervised, and accompanied by standardized reporting of acceptance, preference, omissions, commissions, failed generations, reporting time, corrections, and editing burden.

## Introduction

Medical report generation is a central bottleneck in imaging practice. In radiology, pathology, and endoscopy, clinicians must convert findings into coherent, clinically actionable reports under rising volume, workforce pressure, fatigue, and time constraints. Clinical documentation consumes substantial physician time, and reporting work is a major part of the information-transfer burden in image-based specialties [1]. Reports are also safety-critical communication artifacts: omissions, unsupported statements, ambiguous impressions, and delays in finalization can affect downstream clinical decisions. This reporting burden has therefore made automation and decision support an important target.

Earlier medical imaging report generation research predated the current large language model (LLM) wave and relied on neural architectures designed for specific text generation tasks rather than general purpose foundation models [2,3]. More recent systems based on LLMs have been reported for radiology impression drafting and lumbar spine magnetic resonance imaging (MRI) reporting [4,5], chest radiography and computed tomography (CT) report generation [6,7], endoscopy reporting [8,9], multimodal clinical or PACS (picture archiving and communication system)–linked applications [10,11], oral radiology and multimodal spinal MRI tasks [12,13], and ultrasound reporting [14]. These systems differ substantially in their inputs and intended roles: some generate complete reports from images, some draft impressions from existing findings, and others restructure or edit clinician-authored text. Treating these workflows as a single intervention can obscure important differences in risk, human oversight, and measurable benefit. Outcome interpretation, therefore, requires task-level classification rather than generic aggregation of all report-generation systems.

The central translational question remains unresolved: do these systems improve clinical reporting, or do they generate plausible but unsafe text? The literature is broad but fragmented across endoscopy [8,9], mixed-modality or workflow-linked settings [10,11], chest x-ray and cross-sectional imaging [15,16], pathology [17,18], and workflows ranging from autonomous generation to drafting, editing, and structuring support. This breadth does not yet translate into coherent clinical inference because studies differ in clinical input, generation task, comparator, human oversight, and outcome definition. A study that reports linguistic similarity to a reference report does not answer the same question as a study that measures missed findings, hallucinated statements, clinician editing burden, or final report acceptance.

The safety and workflow context for LLM-based report generation is broader than report text quality alone. Automation bias can reduce human verification of decision-support outputs when users overrely on automated suggestions, especially when verification is complex [19]. Clinical AI safety literature also emphasizes that bias, distribution shift, unclear failure modes, and insufficient monitoring can create risks even when model performance appears favorable in development settings [20]. For LLMs specifically, medical use raises concerns about fluent but unsupported outputs [21], and hallucination is a recognized limitation of natural language generation systems [22]. Recent radiology guidance on generative AI similarly emphasizes human oversight, local validation, monitoring, and workflow-specific safeguards before clinical deployment [23]. These risks make safety and workflow end points central to assessing clinical usefulness rather than secondary technical details.

Existing reviews show why a new synthesis is needed now. Reviews and commentaries have described the rapid emergence of AI report generation, prompt engineering and fine-tuning, structured reporting, foundation models in imaging, and endoscopy applications [24-28]. More recent systematic and scoping reviews have summarized automated radiology report-generation methods, structured-reporting applications, and deep learning trends [29-31], while others have focused on LLM use in radiology reports, report readability or simplification, and broader radiology adoption trajectories [32-34]. These reviews are valuable, but generally emphasize technical progress, radiology-centered use cases, readability, or benchmark-oriented evaluation. They do not resolve whether the peer-reviewed evidence base reports on clinically interpretable safety and workflow outcomes consistently enough to guide supervised implementation or cumulative clinical inference. In particular, prior syntheses have not consistently separated autonomous image-to-report generation from supervised drafting or report-structuring support, although those categories carry different clinical responsibilities and failure modes.

This gap is important because technical evaluation and clinical decision-making require different evidence. Many studies still emphasize benchmark language metrics such as BLEU (Bilingual Evaluation Understudy), ROUGE (Recall-Oriented Understudy for Gisting Evaluation), CIDEr (Consensus-Based Image Description Evaluation), and BERTScore (a BERT-based semantic similarity metric, where BERT stands for Bidirectional Encoder Representations from Transformers) [35-38], while clinically important outcomes are reported less consistently. For this review, effectiveness was defined as expert acceptance and blinded expert preference; safety as clinically significant error rate, omission error rate, and commission error rate; and workflow burden as reporting time, number of corrections, edit distance, and editing burden. Here, omission errors are missed clinically relevant findings, and commission errors are unsupported or hallucinated findings introduced into the report. Benchmark similarity metrics help technical development but do not show whether a generated report missed a critical lesion, introduced a misleading statement, or reduced physicians’ finalization effort. Image-aware evaluation frameworks have begun to address this gap, but they remain uncommon and unharmonized for clinical synthesis [39]. In addition, many reports provide reader ratings or preference judgments without case-level denominators, independent validation, or a clear account of failed generations. Consequently, individual positive studies do not yet establish whether the field is approaching clinical readiness or overstating progress through benchmark-dominated evaluation.

The objective of this systematic review was to assess the effectiveness (expert acceptance and blinded preference), safety (clinically significant errors, omission errors, and commission errors), and workflow burden (reporting time, corrections, edit distance, and editing burden) of LLM-based medical report generation across imaging modalities and related clinical reporting contexts. To support clinically meaningful interpretation, we separated task types and levels of human oversight so that autonomous image-to-report generation, supervised drafting, impression generation, and report-structuring support were not treated as equivalent interventions.

## Methods

### Protocol and Registration

This systematic review was conducted in accordance with the PRISMA (Preferred Reporting Items for Systematic Reviews and Meta-Analyses) 2020 statement (Checklist 1) [40]. Literature search reporting followed the PRISMA-S (Preferred Reporting Items for Systematic Reviews and Meta-Analyses Literature Search Extension) [41], and narrative synthesis was reported with attention to the Synthesis Without Meta-Analysis (SWiM) guidance [42]. The review question, primary outcomes, and subgroup structure were specified a priori, and the review was registered with PROSPERO (International Prospective Register of Systematic Reviews) 2026 (CRD420261302844) before formal screening began. The prespecified review protocol and extraction backbone are available from the corresponding author upon reasonable request. The PRISMA-S checklist was completed item by item in the Checklist 1, and when a PRISMA-S item was not part of the search methodology or was not applicable, it was explicitly marked and justified.

### Eligibility Criteria

Eligible studies evaluated generative AI systems based on LLMs for generating an imaging report, report draft, or diagnostic impression from medical imaging, pathology whole-slide imaging, endoscopy, or multimodal, clinically relevant inputs. For eligibility, an intervention was considered eligible if an LLM, multimodal LLM, vision-language foundation model, or a language model component contributed directly to clinical report text generation, diagnostic impression generation, or clinical report drafting. Image classifiers, segmentation tools, retrieval systems, and image captioning systems were not eligible unless a language model or foundation model contributed directly to report generation.

Prespecified task types were report generation from images, generation of impressions from findings, and report drafting. Human oversight was categorized as autonomous generation, human-supervised drafting, or report editing or structuring support. Autonomous generation referred to systems that generated reports or report outputs from images or clinical inputs before human evaluation. Human-supervised drafting referred to workflows in which clinicians actively used, or iteratively collaborated on, AI-generated text during drafting. Report editing or structuring support referred to settings in which AI was applied after source report text, findings, or draft content was already available. We included randomized and nonrandomized comparative studies, reader studies, prospective workflow studies, retrospective validation studies, and single-arm technical validation studies if they used an explicit expert or clinical reference standard. Conference abstracts, reviews, editorials, letters without original data, dissertations, protocols, and preprints were excluded. Because preprints were excluded, this review was designed to evaluate peer-reviewed clinical evidence rather than the absolute frontier of technical model capability.

Effectiveness outcomes were expert acceptance rate and blinded expert preference. Safety outcomes were clinically significant error rate, omission error rate, and commission error rate. Workflow burden outcomes were reporting time, number of corrections, edit distance, and editing burden after generation. Omission errors were defined as missed clinically relevant findings, whereas commission errors were defined as unsupported or hallucinated findings introduced into the generated report. Exploratory outcomes included discrepancy rates between findings and impressions, and text similarity or language quality metrics, such as BLEU, ROUGE, CIDEr, and BERTScore.

### Information Sources

We searched PubMed/MEDLINE, Embase, Web of Science Core Collection, Scopus, and the Cochrane Library from January 1, 2016, through May 15, 2026. Europe PMC, OpenAlex, and reference lists of eligible studies and relevant reviews were used as supplemental sources through May 17, 2026, to identify additional records. Study registries were not used as bibliographic search sources because the eligibility criteria targeted completed peer-reviewed studies of medical report generation; this nonuse is reported explicitly in the PRISMA-S checklist. For studies that reported clinically important workflow or safety outcomes in nonconvertible formats, corresponding authors were contacted to request additional analyzable summary statistics or case-level denominator clarification.

### Search Strategy

The database strategy combined controlled vocabulary and free-text terms related to LLMs, multimodal LLMs, vision-language models, foundation models, and generative AI; report generation, report drafting, diagnostic impression generation, and structured reporting; and imaging domains including radiology, CT, MRI, radiography, pathology whole-slide imaging, ultrasound, endoscopy, and other medical image or signal interpretation settings. Exact database-specific search strings, interfaces, limits, dates, and record counts for PubMed/MEDLINE, Embase, Web of Science Core Collection, Scopus, and the Cochrane Library are provided in Multimedia Appendix 1; the completed PRISMA-S item-level reporting checklist is provided in the reporting guideline checklist file. Database searches were first completed on March 18, 2026, and were updated in May 2026 before final analysis. Across the original and updated database searches, 6936 records were identified before deduplication. Records retrieved in both searches were deduplicated by DOI, PMID, or normalized title and were counted once. After removal of 3008 duplicate records and 499 records already represented in the search corpus, 3429 database records remained for title and abstract screening.

### Study Selection

Title and abstract screening and full-text eligibility assessment were performed independently by 2 reviewers after deduplication, with disagreements resolved by discussion or adjudication by a third reviewer. No interrater reliability coefficient was generated; therefore, reviewer agreement is described procedurally rather than as a kappa statistic. Full-text exclusions were logged with one dominant reason per report.

### Data Extraction

Extraction frameworks at the study and outcome levels were used, and key extraction fields were checked in duplicate for studies that contributed clinically important safety or workflow outcomes. Extracted variables included study characteristics, clinical specialty, imaging modality, task type, level of human oversight, study design, validation setting, model characteristics, comparator type, reference standard, outcome definitions, and raw data sufficient for effect-size computation when available.

### Risk of Bias and Certainty Assessment

Risk of bias was assessed according to study design. Comparative and workflow studies were appraised using ROBINS-I (Risk of Bias in Non-Randomized Studies of Interventions) logic [43], with reporting appraisal informed by CLAIM (Checklist for Artificial Intelligence in Medical Imaging) as an adjunct for assessing AI reporting quality [44]. Retrospective technical validation studies that were not well captured by conventional intervention tools were assessed using a prespecified custom AI validation risk-of-bias framework. The core domains of that framework were representativeness of case selection, reference standard quality, blinding of expert assessment, validation independence, data leakage risk, model or prompt transparency, handling of failed or missing generations, and risk from outcome definition or selective reporting.

For the custom framework, moderate concern was assigned when a limitation was present but limited in scope or of limited consequence for clinical interpretation. High concern was assigned when a limitation was important and likely to influence interpretation, such as unclear validation independence, incomplete blinding, limited external validation, or incomplete failure reporting. Serious concern was assigned when a limitation could directly undermine clinical validity, such as likely data leakage, clearly nonrepresentative case selection, materially weak or unclear reference standards, the absence of handling of failed generations when failures could alter outcome interpretation, or strongly selective reporting of clinically important outcomes. The framework was used as a structured, qualitative, domain-based appraisal rather than a formally validated measurement instrument, and no interrater reliability coefficient was generated for this review. Its judgments should, therefore, be interpreted as transparent qualitative risk signals rather than as validated scale scores.

Because no outcomes met the criteria for meta-analysis, certainty was judged using a narrative certainty assessment informed by GRADE (Grading of Recommendations Assessment, Development, and Evaluation) domains rather than rating pooled effect estimates [45]. We summarized these judgments in a GRADE Summary of Findings table format, using outcome-domain study counts from the selected clinically interpretable end point studies, and reported relative effects as not pooled or not estimable when compatible effect structures were unavailable.

### Data Synthesis

Eligibility for quantitative synthesis was assessed at the outcome level according to clinical coherence, task structure, comparator or reference standard design, and availability of analyzable event counts or summary statistics. For outcomes amenable to direct quantitative reconstruction, planned effect measures were risk differences for dichotomous outcomes and mean differences for continuous outcomes, with 95% CIs when derivable from published data. Outcomes reported across reader-case pairs were not treated as independent case-level events when clustering could not be reconstructed.

Quantitative synthesis was prespecified only for outcomes reported by at least 2 studies with compatible definitions, task structures, comparator or reference standard designs, and analyzable data structures. Outcomes that did not meet these criteria were summarized using structured narrative synthesis. When individual studies provided sufficient data, targeted quantitative reconstruction was performed to support interpretation without pooling. Narrative synthesis was organized by modality, task type, outcome domain, and level of human oversight. Formal subgroup, sensitivity, heterogeneity, and reporting bias analyses were reserved for poolable synthesis sets.

### Deviations From Protocol

The protocol anticipated quantitative synthesis when clinically and methodologically compatible synthesis sets were available. Supplemental citation discovery and reference screening were added to improve retrieval completeness. These procedures did not change the review question, primary outcomes, eligibility framework, or planned subgroup structure. When outcomes did not meet pooling criteria, synthesis followed the prespecified nonpooled approach of structured narrative synthesis with targeted quantitative reconstruction of individual studies where defensible.

## Results

### Study Selection

**Figure 1.**
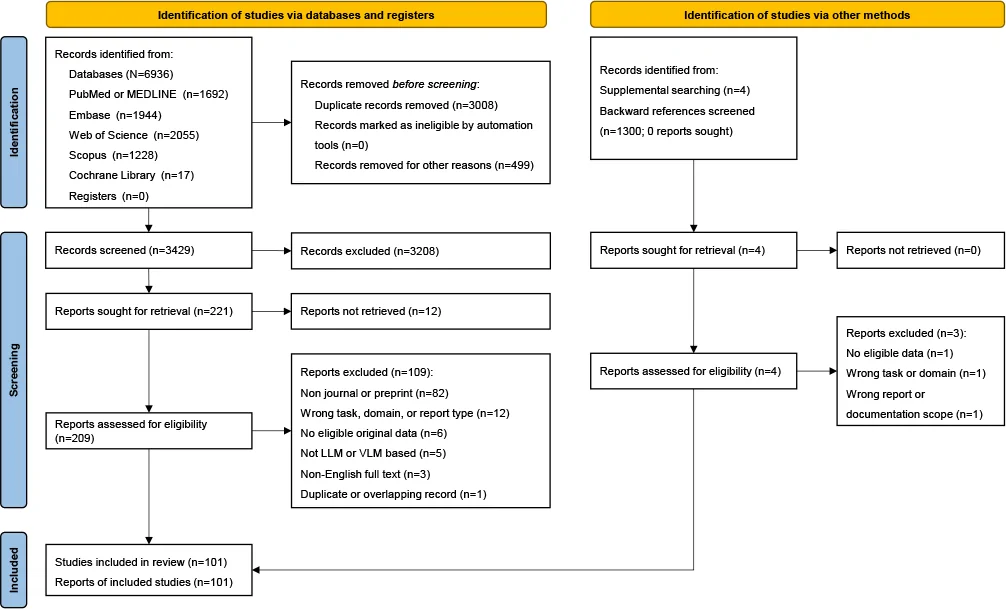
PRISMA (Preferred Reporting Items for Systematic Reviews and Meta-Analyses) 2020 flow diagram for the systematic review. The figure summarizes database searching through May 15, 2026, other-method identification through supplemental citation discovery and backward reference screening, full-text assessment, and final inclusion of 101 studies. LLM: large language model; VLM: vision-language model.

The full study selection process is shown in Figure 1. Across the original and updated database searches, 6936 records were identified before deduplication. After removal of 3008 duplicate records and 499 records already represented in the search corpus, 3429 database records proceeded to title and abstract screening. Public supplemental screening and citation-discovery screening contributed 1 additional eligible study, whereas backward reference screening did not identify additional reports for full-text assessment. Overall, 101 studies met the inclusion criteria for this systematic review. Study-level records are provided in Supplementary Table S1 in Multimedia Appendix 2, and full-text exclusions are listed in Supplementary Table S2 in Multimedia Appendix 2.

### Study Characteristics

**Figure 2.**
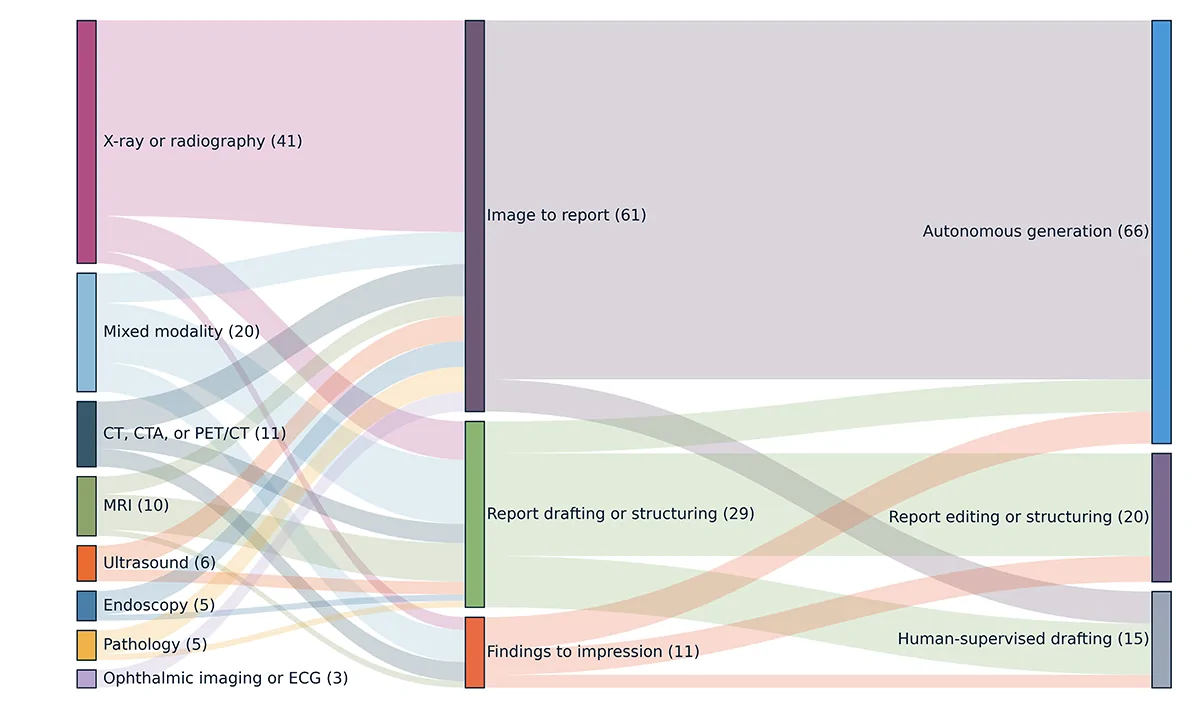
Sankey diagram linking clinical modality or report source, report generation task, and AI role in the reporting workflow for the 101-study evidence base. Flow width is proportional to the number of studies in each pathway. The figure shows continued concentration in x-ray or radiography, report generation from images, and autonomous generation, while also showing smaller evidence streams in computed tomography (CT), computed tomography angiography (CTA), or positron emission tomography (PET)/CT; magnetic resonance imaging (MRI); ultrasound; endoscopy; pathology; ophthalmic imaging or electrocardiogram (ECG); mixed modality; human-supervised drafting; and report editing or structuring workflows.

**Figure 3.**
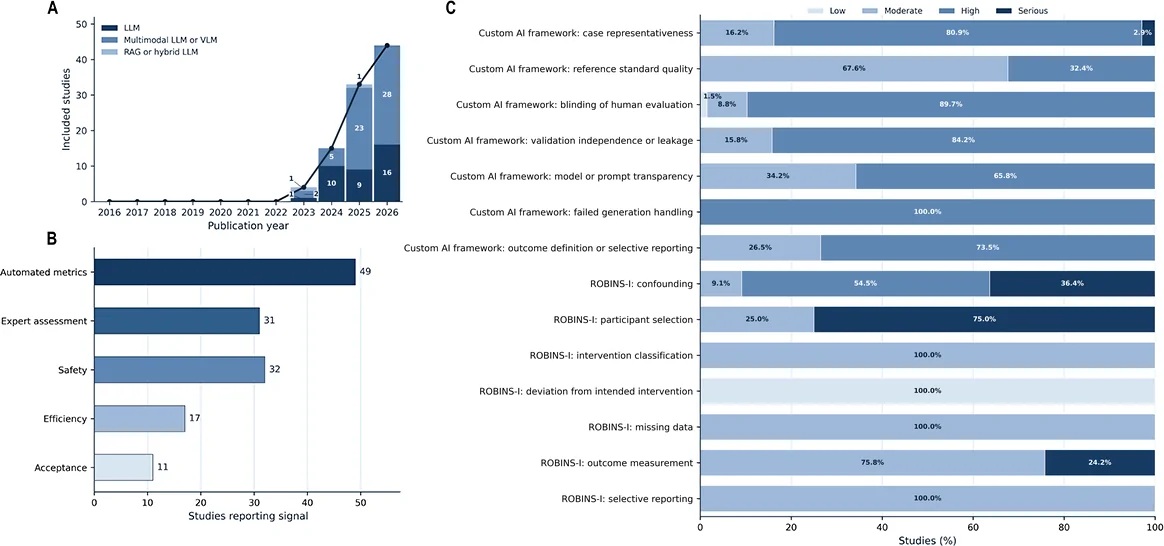
Temporal evolution, evaluation signals, and domain-level risk of bias. Panel A summarizes annual counts among studies for which a publication year was captured, stratified by broad model family or by multimodal or hybrid grouping. Panel B displays nonexclusive standardized evaluation signal categories across the 101-study evidence base; individual automated metric names were grouped under a single automated metrics category because studies used heterogeneous benchmark measures. Panel C summarizes domain-level risk-of-bias judgments across custom AI validation domains (n=68) and ROBINS-I-informed domains (n=33). LLM: large language model; RAG: retrieval-augmented generation; ROBINS-I: Risk of Bias in Non-Randomized Studies of Interventions; VLM: vision-language model.

The included literature was broad in technical scope but remained concentrated in a limited set of clinical scenarios (Table 1). Of the 101 included studies, 68 (67.3%) were retrospective or technical validation studies, 28 (27.7%) were nonrandomized comparative, reader, or workflow studies, and 5 (5.0%) were prospective studies or real-world workflow or validation studies. Chest x-ray remained the largest modality group (36, 35.6%), followed by mixed-modality settings (20, 19.8%), CT, CT angiography, or positron emission tomography/CT (11, 10.9%), MRI (10, 9.9%), ultrasound (6, 5.9%), endoscopy (5, 5.0%), pathology reporting settings (5, 5.0%), other x-ray or radiography (5, 5.0%), ophthalmic imaging (2, 2.0%), and electrocardiogram (1, 1.0%). By task type, 61 (60.4%) studies evaluated report generation from images, 29 (28.7%) evaluated report drafting or structured report generation, and 11 (10.9%) evaluated impression or diagnostic conclusion generation from findings or reports. By human oversight, 66 (65.3%) studies were classified as autonomous generation, 15 (14.9%) as human-supervised drafting, and 20 (19.8%) as report editing or structuring support. The relationship among modality, task type, and human oversight is shown in Figure 2, and the rapid expansion of the literature after 2023 is summarized in Figure 3A.

**Table 1.** Evidence profile of included studies.

Domain and category	Studies, n (%)
Overall	
Total included studies	101
Study design	
Retrospective or technical validation	68 (67.3)
Nonrandomized comparative, reader, or workflow study	28 (27.7)
Prospective or real-world workflow or validation study	5 (5.0)
Clinical source	
Chest x-ray	36 (35.6)
Mixed modality	20 (19.8)
CT^a^, CTA^b^, or PET/CT^c^	11 (10.9)
MRI^d^	10 (9.9)
Ultrasound	6 (5.9)
Endoscopy	5 (5.0)
Pathology report or whole-slide image	5 (5.0)
Other x-ray or radiography	5 (5.0)
Ophthalmic imaging	2 (2.0)
Electrocardiogram	1 (1.0)
Generation task	
Image to report generation	61 (60.4)
Report drafting or structuring	29 (28.7)
Findings or report to impression generation	11 (10.9)
AI role in reporting workflow	
Autonomous generation	66 (65.3)
Human-supervised drafting	15 (14.9)
Report editing or structuring support	20 (19.8)
Overall risk of bias	
Low	0 (0)
Moderate	15 (14.9)
High	72 (71.3)
Serious	14 (13.9)

a CT: computed tomography.

b CTA: computed tomography angiography.

c PET: positron emission tomography.

d MRI: magnetic resonance imaging.

Outcome reporting was imbalanced. Benchmark metrics such as BLEU, ROUGE, CIDEr, and BERTScore were common, especially in chest x-ray and technical validation studies, whereas clinically important outcomes, including significant errors, omissions, commissions, expert acceptance, blinded preference, reporting time, and edit burden, were inconsistently reported and used heterogeneous denominators. Thirty-six studies reported at least one clinically relevant human evaluation, safety, workflow, report quality, acceptability, or time outcome, but these outcomes differed by task, comparator, denominator, and reporting format. Benchmark-heavy chest x-ray studies were represented among the included studies [46-50]. Standardized evaluation signal reporting is summarized in Figure 3B. The subset of studies with clinically interpretable effectiveness, safety, and workflow-burden end points is summarized in Table 2.

**Table 2.** Selected studies with clinically interpretable evaluation end points^a^.

Study and year	Clinical source	Generation task	AI role in reporting workflow	Assessment method	Reported evaluation outcomes
Hong et al [51], 2025	Chest x-ray	Image to report	Autonomous generation	Human reader assessment	Acceptance, safety
Tanno et al [52], 2025	Chest x-ray	Image to report	Human-supervised drafting	Human reader assessment	Acceptance, safety
Wang et al [53], 2026	MRI^b^	Findings to impression	Report editing or structuring support	Human reader assessment	Accuracy, efficiency
Serapio et al [54], 2024	Mixed modality	Findings to impression	Human-supervised drafting	Workflow comparison	Efficiency
Li et al [55], 2025	CT^c^	Image to report	Autonomous generation	Human expert assessment	Report quality, accuracy
Luo et al [56], 2026	Endoscopy	Image to report	Autonomous generation	Human expert assessment	Report quality, accuracy
Ayaz et al [57], 2025	Mixed modality	Image to report	Autonomous generation	Human expert assessment	Report quality
Li et al [58], 2026	Mixed modality	Findings to impression	Human-supervised drafting	Clinical validation	Report quality, accuracy, efficiency
de Margerie-Mellon et al [59], 2026	Mixed modality	Report drafting or structuring	Human-supervised drafting	Workflow comparison	Safety, efficiency
Jiang et al [60], 2026	Endoscopy	Image to report	Autonomous generation	Clinical validation	Safety, accuracy, efficiency
Tekdemir et al [61], 2026	CT	Findings to impression	Autonomous generation	Human expert assessment	Report quality, safety
Lorusso et al [62], 2026	CTA^d^	Findings to impression	Autonomous generation	Reference standard comparison	Accuracy, efficiency
Dong et al [63], 2025	MRI	Report drafting or structuring	Human-supervised drafting	Human expert assessment	Report quality, accuracy, efficiency
Pellegrini et al [64], 2026	Chest x-ray	Image to report	Human-supervised drafting	Workflow comparison	Report quality, efficiency
Tan [65], 2026	Mixed modality	Report drafting or structuring	Human-supervised drafting	Implementation assessment	Acceptance, efficiency
Wang et al [66], 2024	Ultrasound	Report drafting or structuring	Report editing or structuring support	Human expert assessment	Clinician feedback, accuracy, efficiency
Hong et al [67], 2026	Chest x-ray	Image to report	Autonomous generation	Robustness and sensitivity analysis	Acceptance, safety, accuracy
Zanardo et al [68], 2026	MRI	Report drafting or structuring	Autonomous generation	Human expert assessment	Report quality
Xie et al [69], 2025	MRI	Report drafting or structuring	Report editing or structuring support	Human expert assessment	Acceptance, accuracy, efficiency
Woźnicki et al [70], 2025	Chest x-ray	Report drafting or structuring	Report editing or structuring support	Reference standard comparison	Report quality, accuracy
Shao et al [71], 2025	Ophthalmic imaging	Image to report	Autonomous generation	Human expert assessment	Report quality

a Categories are standardized for scanability and do not imply comparable outcome definitions across studies. Human-supervised drafting denotes AI involvement during report drafting, whereas report editing or structuring support denotes AI use after source report text, findings, or draft content were already available.

b MRI: magnetic resonance imaging.

c CT: computed tomography.

d CTA: computed tomography angiography.

### Risk of Bias and Certainty of Evidence

The risk-of-bias assessment framework is provided in Supplementary Table S3A in Multimedia Appendix 2. Overall risk of bias was moderate in 15 studies, high in 72, and serious in 14; no study was at low risk (Table 3). Although the evidence base included multicenter, external validation, and prospective validation studies, most studies were vulnerable to retrospective selection, incomplete external validation, nonrandomized workflow or reader comparisons, incomplete failure reporting, limited transparency in prompting or model handling, and selective emphasis on benchmark outcomes. Recurrent custom-framework concerns involved validation independence or leakage risk, absent or unclear blinding, heterogeneous outcome definitions, and poor accounting for failed or unusable reports. Risk-of-bias judgments are summarized in Table 3; complete study-level judgments are provided in Supplementary Table S3B in Multimedia Appendix 2; and domain-level bias patterns are shown in Figure 3C.

**Table 3.** Risk-of-bias summary by assessment domain^a^.

Assessment set and domain	Risk-of-bias judgment distribution, n/N (%)
All studies	
Overall	Low: 0; moderate: 15/101 (14.9); high: 72/101 (71.3); serious: 14/101 (13.9)
Custom AI validation+CLAIM^b^	
Overall	Low: 0; moderate: 12/68 (17.6); high: 54/68 (79.4); serious: 2/68 (2.9)
Case selection	Low: 0; moderate: 11/68 (16.2); high: 55/68 (80.9); serious: 2/68 (2.9)
Reference standard	Low: 0; moderate: 46/68 (67.6); high: 22/68 (32.4); serious: 0
Blinding	Low: 1/68 (1.5); moderate: 6/68 (8.8); high: 61/68 (89.7); serious: 0
Validation or leakage	Low: 0; moderate: 9/68 (13.2); high: 57/68 (83.8); serious: 2/68 (2.9)
Model or prompt transparency	Low: 0; moderate: 41/68 (60.3); high: 27/68 (39.7); serious: 0
Failure handling	Low: 0; moderate: 0; high: 66/68 (97.1); serious: 2/68 (2.9)
Outcome reporting	Low: 0; moderate: 18/68 (26.5); high: 50/68 (73.5); serious: 0
ROBINS-I^c^+CLAIM	
Overall	Low: 0; moderate: 3/33 (9.1); high: 18/33 (54.5); serious: 12/33 (36.4)
Confounding	Low: 0; moderate: 3/33 (9.1); high: 18/33 (54.5); serious: 12/33 (36.4)
Participant selection	Low: 0; moderate: 23/33 (69.7); high: 0; serious: 10/33 (30.3)
Intervention classification	Low: 0; moderate: 33/33 (100); high: 0; serious: 0
Intervention deviations	Low: 33/33 (100); moderate: 0; high: 0; serious: 0
Missing data	Low: 0; moderate: 33/33 (100); high: 0; serious: 0
Outcome measurement	Low: 0; moderate: 25/33 (75.8); high: 0; serious: 8/33 (24.2)
Reported result selection	Low: 0; moderate: 33/33 (100); high: 0; serious: 0

a This table summarizes risk-of-bias judgments across the 101-study evidence base. Complete study-level judgments are provided in Supplementary Table S3B in Multimedia Appendix 2.

b CLAIM: Checklist for Artificial Intelligence in Medical Imaging.

c ROBINS-I: Risk of Bias in Non-Randomized Studies of Interventions.

Certainty was judged very low across all 4 clinically important outcome domains in the GRADE Summary of Findings table (Table 4). This rating does not mean that all findings were clinically unfavorable; rather, it reflects a high or serious risk of bias, incompatible outcome definitions, indirectness across task types and clinical sources, sparse or nonreconstructable effect data, and concern that clinically unfavorable or failed report-generation outputs may be incompletely reported.

**Table 4.** GRADE^a^ summary of findings for clinically important outcome domains^b^.

Outcome	Studies (n)	Study design	Risk of bias	Inconsistency	Indirectness	Imprecision	Other considerations	Impact	Certainty of the evidence (GRADE)	Importance
Expert acceptance	6	Nonrandomized studies	Serious	Serious	Serious	Serious	Strongly suspected publication or reporting bias	Not pooled. Acceptance measures varied; one clustered reader study reported acceptance of 70.5% (6047/8580) for AI-generated reports compared with 73.3% (6288/8580) for radiologist reports.	Very low	Important
Blinded expert preference	2	Nonrandomized studies	Serious	Serious	Serious	Serious	Strongly suspected publication or reporting bias	Not pooled. One study reported AI-generated reports as equivalent or preferred in 77.7% (233/300) and 56.1% (170/303) of 2 datasets; another study reported that 74.2% (49/66) were indistinguishable from human-written reports.	Very low	Important
Safety outcomes (clinically significant errors, omission errors, and commission errors)	6	Nonrandomized studies	Serious	Serious	Serious	Serious	Strongly suspected publication or reporting bias	Not pooled. Error definitions and denominators varied; one clustered reader study reported false-negative ratings of 18.5% (1584/8580) with AI compared with 17.8% (1527/8580) for radiologist reports, and false-positive ratings of 11.3% (971/8580) compared with 9.7% (831/8580).	Very low	Critical
Workflow burden	11	Nonrandomized studies	Serious	Serious	Serious	Serious	Strongly suspected publication or reporting bias	Not pooled. Workflow effects were mixed: AI shortened MRI^c^ reading time (53 vs 61 s) but increased impression-editing time (18.29 vs 12.20 s) and edit distance (12.32 vs 5.74 words) in another study.	Very low	Important

a GRADE: Grading of Recommendations Assessment, Development, and Evaluation.

b This table follows the GRADEpro (Evidence Prime Inc) or GRADE Summary of Findings structure for narrative evidence. The full review included 101 studies; 36 reported at least one clinically relevant human evaluation, safety, workflow, report quality, acceptability, or time signal; the GRADEpro table uses the 21 selected studies with clinically interpretable end points, as shown in Table 2.

c MRI: magnetic resonance imaging.﻿

### Clinically Relevant Safety and Workflow Outcomes

Table 2 summarizes studies with clinically relevant human evaluation, safety, or workflow findings using standardized outcome domains, while key extractable quantitative findings are reported in this section, where they can be interpreted with study-specific context. In a large chest radiograph study of autonomous AI preliminary reports, acceptance was 70.5% (6047/8580) for AI-generated reports versus 73.3% (6288/8580) for radiologist reports, whereas false-positive findings were 11.3% (1527/8580) versus 9.7% (831/8580) and false-negative findings were 18.5% (1584/8580) versus 17.8% (1527/8580) in clustered reader evaluations [51]. These evaluations were repeated reader-report or reader-case ratings rather than independent case-level events. In a clinician collaboration study for chest radiograph report generation, AI-generated reports were equivalent to or preferred over clinician reports in 77.7% (233/300) of one dataset and 56.1% (170/303) of another when judged by at least half of the raters, but clinically significant errors occurred in both AI-generated and clinician-generated reports [52]. Outcome-level extraction details are provided in Supplementary Table S4 in Multimedia Appendix 2.

Other human evaluation studies of autonomous multimodal generation reported expert indistinguishability, acceptance, or quality ratings in 3D brain CT report generation, bronchoscopy report generation, and medical vision–language reporting across mixed devices [55-57]. Related autonomous report generation studies in chest x-ray, MRI, and fine-tuned LLaMA-based pipelines reported technical performance in additional settings [5,6,72].

Workflow outcomes differed across task types. In a brain MRI reader study, AI assistance improved diagnostic performance and reduced reading time from 61 to 53 seconds, with greater reported benefit among junior radiologists [53]. In contrast, in a study of radiological impression drafting with multiple readers, drafts generated by the model required more editing time than the radiologist baseline (18.29 vs 12.20 s) and a greater edit distance (12.32 vs 5.74 words changed) [54]. For this study of impression drafting, arm-specific means and CIs were sufficient to support the cautious quantitative reconstruction of single studies for sensitivity purposes, but no second clinically comparable workflow study was available for pooling (Supplementary Table S5 in Multimedia Appendix 2) [54]. Additional workflow studies in lumbar spine MRI, breast ultrasound, structured positron emission tomography/CT reporting, and practical drafting with ChatGPT reported on workflow integration in heterogeneous settings [73-76].

Additional studies in the evidence base evaluated blinded radiologist assessment, structured report reader experiments, emergency CT impression drafting with assessment of critical omissions, real-world or pilot reporting workflows, prospective endoscopy report validation, and reporting time comparisons with LLM assistance (Table 2). Reported findings varied by task: some studies reported improved reporting efficiency, structured report usability, or expert acceptability, whereas others reported persistent omissions, hallucinations, lower quality ratings from specialists compared with human reports, or workflow benefits limited to narrow, standardized settings. The characteristics of studies added to the final evidence set are summarized in Supplementary Table S1 in Multimedia Appendix 2 [58-71,77-130].

### Feasibility of Meta-Analysis and Narrative Synthesis

No prespecified effectiveness, safety, or workflow burden outcome had at least 2 studies with directly comparable definitions, coherent designs, and analyzable effect structures suitable for meta-analysis. The expanded evidence base broadened clinical contexts but also added heterogeneity in inputs, outputs, comparators, human oversight, and denominator definitions. The strongest quantitative workflow signal came from the single impression-drafting study in Table 2 and Supplementary Table S5 in Multimedia Appendix 2. Safety and expert evaluation outcomes were usually reported as clustered reader-level counts, threshold preferences, paired overlap summaries, qualitative ratings, or pilot metrics that could not be pooled. These reporting barriers were summarized in Supplementary Table S6 in Multimedia Appendix 2 and structured the narrative synthesis.

No additional unpublished outcome data were available by May 19, 2026; therefore, all quantitative reconstruction and narrative synthesis relied on publicly reported results.

## Discussion

### Principal Findings

This systematic review assessed the effectiveness (expert acceptance and blinded preference), safety (clinically significant errors, omission errors, and commission errors), and workflow burden (reporting time, corrections, edit distance, and editing burden) of LLM-based medical report generation. Relative to these objectives, the principal finding is that the field is active and clinically diverse, but the reported evidence remains too heterogeneous to support stable conclusions about overall effectiveness, safety, or workflow benefit. Effectiveness outcomes suggested possible assistive value in selected settings [51,52], but these outcomes were not consistently paired with case-level safety end points. Safety and workflow burden outcomes were reported with incompatible denominators, task structures, and comparison designs [53,54].

The findings, therefore, support a cautious, task-specific interpretation rather than a general claim that LLM-based report generation is effective or safe. Some supervised workflows appeared acceptable to experts or were useful for selected reporting tasks, but current evidence does not justify autonomous replacement of clinicians [20,23,131]. Benefit and risk differed by task and workflow because impression generation from findings, human-supervised drafting, report editing or structuring, and autonomous image-to-report generation involve different clinical inputs, opportunities for human correction, and failure modes. These task categories should not be treated as interchangeable when interpreting acceptance, reporting errors, workflow burden, or implementation readiness.

### Interpretation and Comparison With Existing Literature

Prior reviews and commentaries have described the rapid emergence of AI report generation, structured reporting, prompt engineering, and foundation-model applications in imaging and endoscopy [24-28]. Recent systematic and scoping reviews have further summarized automated radiology report generation, structured reporting approaches, and deep learning trends [29-31], as well as LLM use in radiology reports, readability, simplification, and broader radiology adoption trajectories [32-34]. These publications establish that the technical literature is active, but leave a practical clinical question unresolved: whether published studies report clinically interpretable effectiveness, safety, and workflow outcomes in a way that can guide supervised implementation. This review differs from those prior reviews by prioritizing outcome definitions, the level of human oversight, and synthesis feasibility rather than technical capability alone. This emphasis allowed us to map not only what models can generate but also how reported outcomes relate to clinical usefulness.

The effectiveness findings need to be interpreted according to the outcomes that were actually reported. Expert acceptance, blinded preference, and report-quality judgments were the most common effectiveness signals, and several studies suggested that generated reports or AI-assisted reports could be acceptable, equivalent, or preferred in selected reader-evaluation settings [51,52]. However, acceptance and preference are not the same as clinical effectiveness unless they are accompanied by evidence that final reports are accurate, clinically complete, and not more burdensome to verify [20,23]. In this review, positive effectiveness signals were often limited by single-study designs, clustered reader ratings, heterogeneous rating scales, and incomplete linkage to omissions, commissions, or final clinician-edited report quality. Therefore, the evidence supports a narrower conclusion: LLM-generated or LLM-assisted report text may be useful in some supervised contexts, but published outcomes do not yet establish a generalizable effectiveness advantage across medical reporting workflows.

The safety and workflow outcomes further explain why broad effectiveness claims would be premature. In chest x-ray and clinician-collaboration settings, expert acceptance or preference could coexist with false-negative findings or clinically significant errors, showing that acceptable language does not necessarily imply safe reporting [51,52]. Workflow findings were also mixed: a brain MRI study reported shorter reading time with AI assistance, whereas an impression-drafting study reported longer editing time and greater edit distance than the radiologist baseline [53,54]. These findings suggest that workflow benefit depends on where the system enters the reporting process, whether clinicians start from images, findings, or draft text, and how much verification or correction the AI output requires.

The nonpoolability of the evidence base is itself an important synthesis finding. Meta-analysis was inappropriate not because publication volume was small, but because the evidence structure was incompatible across studies. Clustered reader-level counts, threshold preference summaries, paired workflow results without reconstructible dispersion, heterogeneous quality scales, pilot outcomes, and unclear denominators prevented valid pooling. This pattern aligns with SWiM principles: when effect estimates cannot be validly combined, transparent, structured narrative synthesis is preferable to forced quantitative aggregation [42]. The expanded search increased clinical breadth but did not create a coherent synthesis set for any primary or key secondary outcome, indicating a reporting-standardization problem that limits cumulative clinical inference.

The dominance of chest x-ray studies also needs careful interpretation. Public corpora such as MIMIC-CXR have accelerated model development and benchmarking [15,16], but repeated use of the same or related datasets can increase the risk of overlapping test sets, model and data leakage, and overly optimistic generalizability. This concern is especially relevant when training, validation, and external testing boundaries are not reported clearly. Endoscopy, pathology whole-slide imaging, CT, MRI, and ultrasound reporting involve different information densities, reference standard structures, and workflow constraints; consequently, evidence from templated chest radiography should not be generalized uncritically to more complex reporting domains.

The safety findings also require interpretation at the level of the clinical task. Human preference or acceptance of generated text should not be interpreted as equivalent to safety unless omissions, commissions, clinically significant discrepancies, and failed generations are measured explicitly [19,21,23]. A fluent report can still omit an important abnormality, introduce an unsupported finding, or increase the review burden for the clinician who must verify it [21-23]. This distinction is especially important for autonomous image-to-report systems, where errors may arise before a clinician’s review, but it also matters for impression-drafting and report-structuring tools because they can shape the language and emphasis of the final report. Accordingly, implementation decisions should require direct measurement of error types in the specific workflow under consideration.

### Clinical and Research Implications

For clinical practice, the real-world implication is that LLM-based reporting systems should be evaluated as supervised assistive tools, not as unsupervised replacements [23,131]. Implementation decisions should be workflow specific because an AI tool that drafts an impression from existing findings has a different safety profile from an autonomous image-to-report model, and a tool that improves readability or structure does not necessarily reduce diagnostic error. Local validation should measure the task actually being deployed, the final clinician-edited report, and the workload created by reviewing or correcting generated text [20,23,131]. This means evaluating systems in the local modality, report template, patient mix, clinician review process, and information-technology environment in which they would actually be used.

For developers and clinical investigators, these findings point toward a staged evidence pathway. Benchmark metrics may be appropriate for early technical screening, but progression toward clinical use should require prospective or externally validated studies with safety outcomes at the case level and reconstructable workflow statistics [20,23,131]. Evaluation should separate omissions from commissions, report failed or unusable generations, state case-level denominators, and measure reporting time, corrections, edit distance, acceptance, blinded preference, and final report quality in realistic workflows [23,44,131]. These reporting elements would make future evidence more useful for health systems because they connect apparent effectiveness to the safety and labor required to produce a clinically usable final report.

For evidence synthesis, the field needs more consistent reporting before meta-analysis can become informative. Future studies should define whether the unit of analysis is the patient, examination, report, reader-report pair, or generated text segment; specify whether evaluators were blinded; report how unusable generations were handled; and provide enough event counts or summary statistics to reconstruct effects [42,44,131]. They should also report effectiveness, safety, and workflow outcomes together rather than presenting acceptance, errors, and time savings as isolated signals. Without these elements, additional studies may increase publication volume without improving certainty or allowing health systems to judge whether LLM-assisted reporting improves outcomes that matter in practice.

### Limitations

This review has limitations. The evidence base was dominated by retrospective studies; prospective workflow research was sparse; and no main outcome supported a robust pooled estimate across multiple directly comparable studies. The custom AI validation framework was prespecified and transparently domain-based, but it was not a formally validated measurement instrument, and interrater agreement statistics were not generated; accordingly, those judgments should be interpreted as structured qualitative bias signals rather than as validated scale outputs. The narrative GRADE-informed certainty assessment was likewise constrained by the absence of pooled estimates; it used GRADE domains to structure certainty judgments but did not produce certainty ratings around pooled effect sizes [45].

Excluding preprints may underrepresent the newest model capabilities in a fast-moving field, but this choice was appropriate for a clinical review focused on peer-reviewed evidence. Some included and excluded studies may share public datasets or related benchmarks, reducing independence across studies [15,16]. We also could not always determine whether model training data overlapped with evaluation data, especially for studies using public radiology corpora or proprietary foundation models. Finally, structured narrative synthesis provides less statistical compression than meta-analysis but better reflects the current nonpoolable evidence structure [42].

### Conclusions and Broader Implications

This review provides a clinically oriented synthesis of LLM-based medical report generation across effectiveness, safety, workflow burden, and human oversight. It differs from radiology-specific, readability-oriented, and benchmark-focused reviews by asking whether peer-reviewed evidence can support clinical judgments about expert acceptance, blinded preference, clinically significant errors, omissions, commissions, reporting time, corrections, edit distance, and editing burden. Its main contribution is to show that the barrier is no longer simply whether models can produce plausible reports; it is whether studies report acceptance, preference, omissions, commissions, failed generations, time, corrections, edit distance, and human oversight well enough to guide practice. The broader real-world implication is that adoption should remain assistive, clinician-supervised, locally validated, and specific to the intended reporting workflow until stronger prospective safety and workflow evidence is available [20,23,131]. In practical terms, LLM-based reporting should be viewed as a candidate workflow aid whose value depends on the clinical task, validation setting, and human review process, not as a general replacement for expert report generation. The next stage of research should therefore move from benchmark-centered demonstrations toward standardized, case-level effectiveness, safety, and workflow burden evaluations that can support both local implementation decisions and future quantitative synthesis.

## Supplementary material

10.2196/97007 Multimedia Appendix 1 Exact search strategies and search update summary.

10.2196/97007 Multimedia Appendix 2 Supplementary evidence tables.

10.2196/97007 Checklist 1 Reporting guideline checklists: PRISMA 2020, PRISMA abstracts, PRISMA-S, and SWiM.

## Abbreviations

BERT: Bidirectional Encoder Representations from Transformers
BLEU: Bilingual Evaluation Understudy
CIDEr: Consensus-Based Image Description Evaluation
CLAIM: Checklist for Artificial Intelligence in Medical Imaging
CT: computed tomography
GRADE: Grading of Recommendations Assessment, Development, and Evaluation
LLM: large language model
MRI: magnetic resonance imaging
PACS: picture archiving and communication system
PRISMA: Preferred Reporting Items for Systematic Reviews and Meta-Analyses
PRISMA-S: Preferred Reporting Items for Systematic Reviews and Meta-Analyses Literature Search Extension
PROSPERO: International Prospective Register of Systematic Reviews
ROBINS-I: Risk of Bias in Non-Randomized Studies of Interventions
ROUGE: Recall-Oriented Understudy for Gisting Evaluation
SWiM: Synthesis Without Meta-Analysis

## References

[1] Sinsky C, Colligan L, Li L (2016). Allocation of physician time in ambulatory practice: a time and motion study in 4 specialties. Ann Intern Med.

[2] Jing B, Xie P, Xing E (2018). On the automatic generation of medical imaging reports. Proc Assoc Comput Linguist Annu Meet.

[3] Chen Z, Song Y, Chang TH, Wan X (2020). Generating radiology reports via memory-driven transformer. Proc Conf Empir Methods Nat Lang Process.

[4] Zhong T, Zhao W, Zhang Y (2026). ChatRadio-Valuer: a chat large language model for generalizable radiology impression generation on multi-institution and multi-system data. IEEE Trans Biomed Eng.

[5] Yeasin M, Moinuddin KA, Havugimana F, Wang L, Park P (2024). Auto-Rad: end-to-end report generation from lumber spine MRI using vision-language model. J Clin Med.

[6] Lee RW, Lee KH, Yun JS, Kim MS, Choi HS (2024). Comparative analysis of M4CXR, an LLM-based chest X-ray report generation model, and ChatGPT in radiological interpretation. J Clin Med.

[7] Chen Z, Bie Y, Jin H, Chen H (2025). Large language model with region-guided referring and grounding for CT report generation. IEEE Trans Med Imaging.

[8] Massimi D, Stefano LD, Rizkala T (2026). Large language model-driven analysis and report generation of endoscopy videos-A pilot study. Dig Endosc.

[9] Zhang X, Zheng X, Li Z (2026). A fine-tuning multimodal large language model for endoscopic report generation. Biomed Signal Process Control.

[10] Joyson JL, Soundar KR, Nancy P (2026). A multi-modal fusion-based deep learning with finetuned LLaMA 3 for lung disease diagnosis using PACS radiology reports. Biomed Signal Process Control.

[11] Liu F, Zhou H, Wang K (2025). MetaGP: a generative foundation model integrating electronic health records and multimodal imaging for addressing unmet clinical needs. Cell Rep Med.

[12] Dasanayaka C, Dandeniya K, Dissanayake MB, Gunasena C, Jayasinghe R (2025). Multimodal AI and large language models for orthopantomography radiology report generation and Q&A. Appl Syst Innov.

[13] Helmy H, Hosseini A, Ibrahim A, Baig-Mirza A, Sadek AR, Serag A (2026). SPINE: segmentation-guided processing and integration of multimodal spinal MRI for natural-language enhanced report generation. Appl Artif Intell.

[14] Li Z, Li M, Wang W, Huang Q (2025). Ultrasound report generation with fuzzy knowledge and multi-modal large language model. Expert Syst Appl.

[15] Wang Z, Liu L, Wang L, Zhou L (2023). R2GenGPT: radiology report generation with frozen LLMs. Meta-Radiology.

[16] Liu Y, Li Y, Wang Z (2024). A systematic evaluation of GPT-4V’s multimodal capability for chest X-ray image analysis. Meta-Radiology.

[17] Kim KA, Hong S, Yoo S, Kang Y, Shim HS (2025). Enhancing structured pathology report generation with foundation model and modular design. IEEE Access.

[18] Yan H, Shao D (2025). Multimodal medical image analysis: integrating LLM and RAG deep learning strategies. J Adv Inf Technol.

[19] Lyell D, Coiera E (2017). Automation bias and verification complexity: a systematic review. J Am Med Inform Assoc.

[20] Challen R, Denny J, Pitt M, Gompels L, Edwards T, Tsaneva-Atanasova K (2019). Artificial intelligence, bias and clinical safety. BMJ Qual Saf.

[21] Lee P, Bubeck S, Petro J (2023). Benefits, limits, and risks of GPT-4 as an AI chatbot for medicine. N Engl J Med.

[22] Ji Z, Lee N, Frieske R (2023). Survey of hallucination in natural language generation. ACM Comput Surv.

[23] Yi PH, Haver HL, Jeudy JJ (2025). Best practices for the safe use of large language models and other generative AI in radiology. Radiology.

[24] Seah JCY, Tang JSN, Tran A (2025). Drafting the future: the dawn of AI report generation in radiology. Radiology.

[25] Kim TT, Makutonin M, Sirous R, Javan R (2025). Optimizing large language models in radiology and mitigating pitfalls: prompt engineering and fine-tuning. Radiographics.

[26] Oda M (2025). Generative AI and foundation models in medical image. Radiol Phys Technol.

[27] Labaki C, Uche-Anya EN, Berzin TM (2024). Artificial intelligence in gastrointestinal endoscopy. Gastroenterol Clin North Am.

[28] Biswas S, Khan S, Awal SS (2024). Can ChatGPT write radiology reports?. Chin J Acad Radiol.

[29] Sloan P, Clatworthy P, Simpson E, Mirmehdi M (2025). Automated radiology report generation: a review of recent advances. IEEE Rev Biomed Eng.

[30] Busch F, Hoffmann L, Dos Santos DP (2025). Large language models for structured reporting in radiology: past, present, and future. Eur Radiol.

[31] Izhar A, Idris N, Japar N (2025). Medical radiology report generation: a systematic review of current deep learning methods, trends, and future directions. Artif Intell Med.

[32] Lee RC, Hadidchi R, Coard MC (2026). Use of large language models on radiology reports: a scoping review. J Am Coll Radiol.

[33] Patwardhan V, Balchander D, Fussell D (2026). Leveraging large language models to enhance radiology report readability: a systematic review. J Am Coll Radiol.

[34] Al Zaabi A, Alshibli R, AlAmri A, AlRuheili I, Lutfi SL (2025). Trends and trajectories in the rise of large language models in radiology: scoping review. JMIR Med Inform.

[35] Papineni K, Roukos S, Ward T, Zhu WJ, Isabelle P, Charniak E, Lin D (2002). Proceedings of the 40th Annual Meeting of the Association for Computational Linguistics.

[36] Lin CY (2004). Text Summarization Branches Out: Proceedings of the ACL-04 Workshop.

[37] Vedantam R, Zitnick CL, Parikh D (2015). CIDEr: consensus-based image description evaluation. Proc IEEE Comput Vis Pattern Recognit Conf (CVPR).

[38] Zhang T, Kishore V, Wu F, Weinberger KQ, Artzi Y (2020). BERTScore: evaluating text generation with BERT. https://openreview.net/pdf?id=SkeHuCVFDr.

[39] Gholipour Picha S, Al Chanti D, Caplier A (2026). Trust but verify: image-aware evaluation of radiology report generators. Mach Learn Appl.

[40] Page MJ, McKenzie JE, Bossuyt PM (2021). The PRISMA 2020 statement: an updated guideline for reporting systematic reviews. BMJ.

[41] Rethlefsen ML, Kirtley S, Waffenschmidt S (2021). PRISMA-S: an extension to the PRISMA statement for reporting literature searches in systematic reviews. Syst Rev.

[42] Campbell M, McKenzie JE, Sowden A (2020). Synthesis without meta-analysis (SWiM) in systematic reviews: reporting guideline. BMJ.

[43] Sterne JA, Hernán MA, Reeves BC (2016). ROBINS-I: a tool for assessing risk of bias in non-randomised studies of interventions. BMJ.

[44] Tejani AS, Klontzas ME, Gatti AA (2024). Checklist for artificial intelligence in medical imaging (CLAIM): 2024 update. Radiol Artif Intell.

[45] Guyatt GH, Oxman AD, Vist GE (2008). GRADE: an emerging consensus on rating quality of evidence and strength of recommendations. BMJ.

[46] Wang X, Wang F, Wang H (2026). Activating associative disease-aware vision token memory for LLM-based X-ray report generation. IEEE Trans Med Imaging.

[47] Noh WJ, Pi SW, Lee BD (2026). Hybrid framework for lesion-aware, clinically coherent chest X-ray report generation using contrastive learning and large language models. Sci Rep.

[48] Han P, Li X, Jing S, Wei J (2026). Dynamic feature fusion guiding and multimodal large language model refining for medical image report generation. Expert Syst Appl.

[49] Jia X, Xiong Y, Pei S, Zhang Y, Yan C, Fang Z (2026). Semantic feedback-based RAG for radiology report generation. Big Data Min Anal.

[50] Wang X, Li Y, Wang F, Wang S, Li C, Jiang B R2GenCSR: mining contextual and residual information for LLM-based radiology report generation. IEEE J Biomed Health Inform.

[51] Hong EK, Ham J, Roh B (2025). Diagnostic accuracy and clinical value of a domain-specific multimodal generative AI model for chest radiograph report generation. Radiology.

[52] Tanno R, Barrett DGT, Sellergren A (2025). Collaboration between clinicians and vision-language models in radiology report generation. Nat Med.

[53] Wang ML, Zhang RP, Wu WJ (2026). Evaluation of large language models for diagnostic impression generation from brain MRI report findings: a multicenter benchmark and reader study. NPJ Digit Med.

[54] Serapio A, Chaudhari G, Savage C (2024). An open-source fine-tuned large language model for radiological impression generation: a multi-reader performance study. BMC Med Imaging.

[55] Li CY, Chang KJ, Yang CF (2025). Towards a holistic framework for multimodal LLM in 3D brain CT radiology report generation. Nat Commun.

[56] Luo X, Huang X, Liang X (2026). Towards automated reporting: a bronchoscopy report dataset for enhancing multimodality large language models. Sci Data.

[57] Ayaz M, Khan M, Saqib M, Khelifi A, Sajjad M, Elsaddik A (2025). MedVLM: medical vision–language model for consumer devices. IEEE Consum Electron Mag.

[58] Li M, Wang Y, Miao Z (2026). Fine-tuned large language model for automated radiology impression generation: a multicenter evaluation. Radiol Artif Intell.

[59] de Margerie-Mellon C, Duron L, Fournier L, Ouakil L, Soulat G, Teixeira PG (2026). Reporting efficiency in diagnostic imaging: can plug-and-play general-purpose large language models outperform conventional speech recognition?. Eur Radiol.

[60] Jiang R, Chen B, Dong Z (2026). Domain specific multimodal large language model for automated endoscopy reporting with multicenter prospective validation. NPJ Digit Med.

[61] Tekdemir H, Çıvgın E, Akpınar Ş (2026). Evaluating large language models for Turkish emergency CT impression drafting: quality, critical omissions, and readability. J Imaging Inform Med.

[62] Lorusso G, Ruscino G, Spitaleri A (2026). Comparative evaluation of large language models for generating CAD-RADS 2.0-compliant diagnostic conclusions in cardiac CT reports. Insights Imaging.

[63] Dong F, Nie S, Chen M, Xu F, Li Q (2025). Keyword-based AI assistance in the generation of radiology reports: a pilot study. NPJ Digit Med.

[64] Pellegrini C, Özsoy E, Gassert FT (2026). Enhancing radiology workflows through collaborative AI-assisted chest X-ray reporting using large vision-language models: a proof-of-concept study. Insights Imaging.

[65] Tan N (2026). Large language model–assisted radiology reporting in a single-radiologist implementation: a retrospective cohort study interpreted through a UTAUT lens. Abdom Radiol.

[66] Wang Z, Guo R, Sun P, Qian L, Hu X (2024). Enhancing diagnostic accuracy and efficiency with GPT-4-generated structured reports: a comprehensive study. J Med Biol Eng.

[67] Hong EK, Lee S, Song O, Leung A, Hammer M, Suh CH (2026). Temperature setting of a multimodal generative artificial intelligence model: association with accuracy and quality of artificial intelligence-generated chest radiograph reports. AJR Am J Roentgenol.

[68] Zanardo M, Albano D, Molinari V (2026). Can AI write reports like a radiologist? A blinded evaluation of large language model-generated lumbar spine MRI reports. Eur Radiol Exp.

[69] Xie Y, Hu Z, Tao H (2025). Large language models for efficient whole-organ MRI score-based reports and categorization in knee osteoarthritis. Insights Imaging.

[70] Woźnicki P, Laqua C, Fiku I (2025). Automatic structuring of radiology reports with on-premise open-source large language models. Eur Radiol.

[71] Shao A, Liu X, Shen W (2025). Generative artificial intelligence for fundus fluorescein angiography interpretation and human expert evaluation. NPJ Digit Med.

[72] Voinea ȘV, Mămuleanu M, Teică RV, Florescu LM, Selișteanu D, Gheonea IA (2024). GPT-driven radiology report generation with fine-tuned Llama 3. Bioengineering (Basel).

[73] Park J, Han K, Oh JS (2026). Prospective evaluation of artificial intelligence (AI) in lumbar spine magnetic resonance imaging (MRI) workflow: from deep learning (DL)–enhanced accelerated acquisition to simultaneous vision language model (VLM)–based automated report generation. Eur J Radiol.

[74] Soleimani M, Seyyedi N, Ayyoubzadeh SM, Kalhori SRN, Keshavarz H (2024). Practical evaluation of ChatGPT performance for radiology report generation. Acad Radiol.

[75] Azhar K, Lee BD, Byon SS, Lee S, Cho KR, Song SE (2026). Semiautomated breast ultrasound report generation using multimodal large language models and deep learning. Front Med (Lausanne).

[76] Chen K, Xu W, Li X (2025). The potential of Gemini and GPTs for structured report generation based on free-text ^18^F-FDG PET/CT breast cancer reports. Acad Radiol.

[77] Raminedi S, Shridevi S, Won D (2024). Multi-modal transformer architecture for medical image analysis and automated report generation. Sci Rep.

[78] Bosbach WA, Heide MS, Gözlügöl N (2026). Conceptual proposal for LLM-generated FDG PET/CT follow-up reports in melanoma: a pilot study on model stability and blinded expert evaluation. Front Nucl Med.

[79] Tran M, Schmidle P, Guo RR (2025). Generating dermatopathology reports from gigapixel whole slide images with HistoGPT. Nat Commun.

[80] Liu D, Li W, Xu N (2026). SpineVLM: a markdown-guided structured fine-tuning framework for spine X-ray report generation. IEEE J Biomed Health Inform.

[81] Ye X, Shen Y, Chen Q (2026). Report generation system for slit-lamp image interpretation using vision-language models. Ophthalmol Ther.

[82] Barzegar M, Rostami H, Sanati A, Afshoon R, Kosari A, Keshavarz A (2026). Enhancing chest X-ray report generation with pathology-guided prompts and vision-language shortcut bias mitigation. Biomed Signal Process Control.

[83] Allaberdiev S, Khan A, Mamarasulov S, Chen X (2026). Chestxgen: dynamic memory–augmented vision-language transformer with context-aware gating for radiology report generation. J Artif Intell Soft Comput Res.

[84] Lin KH, Chang CP, Kuo CT (2026). Automatic speech recognition and large language models for multilingual pathology report generation: proof-of-concept study. JMIR Form Res.

[85] Khatoon S, Mahmood A (2026). Automated radiological report generation from breast ultrasound images using vision and language transformers. J Imaging.

[86] Park YW, Kang M, Ryu H (2026). A robust vision language model for molecular status prediction and radiology report generation in adult-type diffuse gliomas. NPJ Digit Med.

[87] Hossain MZ, Ahmed M, Samu MSS, Islam MR (2025). Privacy-preserving chest X-ray report generation via multimodal federated learning with ViT and GPT2. Biomed Mater Devices.

[88] Hosseini A, Ibrahim A, Serag A (2025). M3: multimodal artificial intelligence for medical report generation and visual question answering from 3D abdominal CT scans. BJR Artif Intell.

[89] Zhang L, Liu M, Wang L (2024). Constructing a large language model to generate impressions from findings in radiology reports. Radiology.

[90] Sun Z, Ong H, Kennedy P (2023). Evaluating GPT-4 on impressions generation in radiology reports. Radiology.

[91] Huang J, Neill L, Wittbrodt M (2023). Generative artificial intelligence for chest radiograph interpretation in the emergency department. JAMA Netw Open.

[92] Mallio CA, Bernetti C, Sertorio AC, Zobel BB (2024). ChatGPT in radiology structured reporting: analysis of ChatGPT-3.5 Turbo and GPT-4 in reducing word count and recalling findings. Quant Imaging Med Surg.

[93] Stephan D, Bertsch A, Burwinkel M (2024). AI in dental radiology improving the efficiency of reporting with ChatGPT: comparative study. J Med Internet Res.

[94] Kim C, Cho S, Yoon JH (2025). Utility of multimodal large language models in analyzing chest X-rays with incomplete contextual information. Healthc Inform Res.

[95] Di Palma L, Darvizeh F, Alì M, Fazzini D (2025). Structured transformation of unstructured prostate MRI reports using large language models. Tomography.

[96] Gupta A, Malhotra H, Garg AK, Rangarajan K (2025). Enhancing radiological reporting in head and neck cancer: converting free-text CT scan reports to structured reports using large language models. Indian J Radiol Imaging.

[97] Lee S, Youn J, Kim H, Kim M, Yoon SH (2025). CXR-LLaVA: a multimodal large language model for interpreting chest X-ray images. Eur Radiol.

[98] Pesapane F, Nicosia L, Rotili A (2025). A preliminary investigation into the potential, pitfalls, and limitations of large language models for mammography interpretation. Discov Oncol.

[99] Hartsock I, Araujo C, Folio L, Rasool G (2026). Improving radiology report conciseness and structure via local large language models. J Imaging Inform Med.

[100] Silbergleit M, Tóth A, Chamberlin JH (2025). ChatGPT vs Gemini: comparative accuracy and efficiency in CAD-RADS score assignment from radiology reports. J Imaging Inform Med.

[101] Adams LC, Truhn D, Busch F (2023). Leveraging GPT-4 for post hoc transformation of free-text radiology reports into structured reporting: a multilingual feasibility study. Radiology.

[102] Jiang H, Xia S, Yang Y (2024). Transforming free-text radiology reports into structured reports using ChatGPT: a study on thyroid ultrasonography. Eur J Radiol.

[103] Hasani AM, Singh S, Zahergivar A (2024). Evaluating the performance of generative pre-trained transformer-4 (GPT-4) in standardizing radiology reports. Eur Radiol.

[104] Tsaniya H, Fatichah C, Suciati N, Obi T, Lee JS (2025). Medical report generation with knowledge distillation and multi-stage hierarchical attention in vision transformer encoder and GPT-2 decoder. IEEE Access.

[105] Yan L, Zhou X, Wang Y, Chang X, Li Q, Han G (2025). Automated ultrasound diagnosis via CLIP-GPT synergy: a multimodal framework for image classification and report generation. IEEE Access.

[106] López-Úbeda P, Martín-Noguerol T, Escartín J, Cabrera-Zubizarreta A, Luna A (2025). Automated MRI pituitary structured reporting from free-text using a fine-tuned Llama model: a feasibility study. Jpn J Radiol.

[107] Tian W, Huang X, Cheng T (2025). A medical multimodal large language model for pediatric pneumonia. IEEE J Biomed Health Inform.

[108] Li Y, Lin Z, Zhang Q (2026). Towards generalizable pathology reports via a multimodal LLM with the multicenter in-context learning. Med Image Anal.

[109] García Hidalgo C, Consentino Hernández JA, Cayuela Espí JV (2026). Informe radiológico estructurado asistido por modelos de lenguaje en residentes de Radiología: piloto de implementación en Urgencias [Article in Spanish]. Rev Esp Edu Med.

[110] Tasneem N, van der Pol CB, Zahoor A (2026). From radiology findings to artificial intelligence–powered impressions: a retrospective study on the comparative performance of recent large language models. Intell Med.

[111] Sheng W, Wang Y, Xiao L (2026). BI-RADS-compliant structured mammography reporting using locally deployed large language models under privacy constraints. Eur Radiol.

[112] Liu R, Bai Y, Yue X, Zhang P (2026). Teaching multimodal LLMs to comprehend 12-lead electrocardiographic images. NPJ Digit Med.

[113] Lee C, Park S, Shin CI (2026). Read like a radiologist: efficient vision-language model for 3D medical imaging interpretation. Med Image Anal.

[114] Alkhaldi A, Alnajim R, Alabdullatef L, Alyahya R, Chen J, Zhu D (2026). MiniGPT-MED: a unified vision-language model for radiology image understanding. Trans Mach Learn Res.

[115] Abdaoui H, Barbaria S, Dergaa I (2026). MedFusionT5: cross-modal attention boosts semantic quality and reduces hallucinations in dental AI. Int Dent J.

[116] Mao Y, Xu W, Qin Y, Gao Y (2026). CT-Agent: a multimodal-LLM agent for 3D CT radiology question answering. Sci China Inf Sci.

[117] Rouhizadeh H, Sandralegar A, Yazdani A (2026). The detectability paradox: bilingual medical report generation with open-weight models and the limits of human oversight. J Am Med Inform Assoc.

[118] Zhang Y, Pan Y, Zhong T (2024). Potential of multimodal large language models for data mining of medical images and free-text reports. Meta-Radiology.

[119] Bosbach WA, Senge JF, Nemeth B (2024). Ability of ChatGPT to generate competent radiology reports for distal radius fracture by use of RSNA template items and integrated AO classifier. Curr Probl Diagn Radiol.

[120] Fang J, Xing S, Li K, Guo Z, Li G, Yu C (2025). Automated generation of chest X-ray imaging diagnostic reports by multimodal and multi granularity features fusion. Biomed Signal Process Control.

[121] Edirisinghe D, Nimalsiri W, Hennayake M, Meedeniya D, Lim G (2025). Chest X-ray report generation using abnormality guided vision language model. IEEE Access.

[122] Li H, Wang H, Sun X, He H, Feng J (2025). Context-enhanced framework for medical image report generation using multimodal contexts. Knowl Based Syst.

[123] Lin Z, Hong Z, Zhou Z (2026). DC-RRG: diagnosis-centered cascaded radiology report generation. Expert Syst Appl.

[124] Izhar A, Idris N, Japar N (2025). Engaging preference optimization alignment in large language model for continual radiology report generation: a hybrid approach. Cogn Comput.

[125] Leonardi G, Portinale L, Santomauro A (2024). Enhancing radiology report generation through pre-trained language models. Prog Artif Intell.

[126] Aslam SM (2025). Generative artificial intelligence for the automated generation of medical reports: a modified poor and rich optimization (MPRO)–based approach. Traitement du Signal.

[127] Deng Q, Huang Z, Wang Y (2026). Grounded knowledge–enhanced medical vision–language pre-training for chest X-ray. Biomed Signal Process Control.

[128] Li Y, Liu Y, Wang Z (2025). S-RRG-Bench: structured radiology report generation with fine-grained evaluation framework. Meta-Radiology.

[129] Ucan M, Kaya B, Kaya M (2025). Turkish chest X-ray report generation model using the Swin enhanced yield transformer (Model-SEY) framework. Diagnostics (Basel).

[130] Rakibul Islam M, Zahid Hossain M, Ahmed M, Sharmin Sultana Samu M (2025). Vision-language models for automated chest X-ray interpretation: leveraging ViT and GPT-2. Eng Rep.

[131] Vasey B, Nagendran M, Campbell B (2022). Reporting guideline for the early-stage clinical evaluation of decision support systems driven by artificial intelligence: DECIDE-AI. Nat Med.

