# Supplementary material for: Effectiveness, Safety, and Workflow Burden of Large Language Model–Based Medical Report Generation: Systematic Review
Source: J Med Internet Res. 2026 Sep 9;28:e97007. doi: 10.2196/97007 (PMC13557449; doi:10.2196/97007)
Supplement: Supplementary file 1 [file jmir-v28-e97007-s001.docx]

**Supplementary Appendix 1. Exact Search Strategies and Search Update Summary**

Coverage: January 1, 2016, to the May 2026 updated search.

# Search Run Summary

| **Source** | **Interface / vendor** | **Date searched** | **Records retrieved before deduplication** |
| --- | --- | --- | --- |
| PubMed/MEDLINE | PubMed / NCBI E-utilities | 2026-05-14 | 1419 |
| Embase | Embase institutional interface | 2026-05-14 | 1641 |
| Web of Science Core Collection | Web of Science | 2026-05-14 | 1462 |
| Scopus | Scopus | 2026-05-15 | 1041 |
| Cochrane Library | Cochrane Library Search Manager | 2026-05-14 | 16 |

Formal database total before deduplication: 5579 records. Formal database total after deduplication: 3001 records. Records already represented in the original March 18, 2026 corpus: 499. New formal records screened during the updated search: 2502.

# Search Principles

This expanded strategy prioritizes sensitivity and uses controlled vocabulary, field modifiers, and broader synonyms. Outcome terms are intentionally not required, because using terms such as safety, workflow, acceptance, omission, or commission could miss eligible report generation studies that did not label their endpoints in those terms.

The strategy uses 3 concept blocks:

1. Generative AI / LLM / VLM / foundation-model terms

2. Report generation / impression generation / report drafting terms

3. Medical imaging / pathology / endoscopy domain terms

Screening rather than restrictive search exclusions determined final eligibility. Database filters were used for date range, English language, and peer-reviewed article type when supported by the database interface, while record counts before deduplication were preserved for transparency.

# PubMed/MEDLINE

Filters applied or used when available:

- Date: January 1, 2016, to May 14, 2026
- Language: English
- Article type: Journal Article when supported by the database interface

```text

(

"Large Language Models"[Mesh]

OR "Artificial Intelligence"[Mesh]

OR "Natural Language Processing"[Mesh]

OR "Machine Learning"[Mesh]

OR "large language model*"[tiab]

OR "large-language model*"[tiab]

OR LLM[tiab]

OR LLMs[tiab]

OR "multimodal large language model*"[tiab]

OR "multi-modal large language model*"[tiab]

OR MLLM[tiab]

OR MLLMs[tiab]

OR "vision-language model*"[tiab]

OR "vision language model*"[tiab]

OR "large vision-language model*"[tiab]

OR "large vision language model*"[tiab]

OR VLM[tiab]

OR VLMs[tiab]

OR LVLM[tiab]

OR LVLMs[tiab]

OR "foundation model*"[tiab]

OR "generative artificial intelligence"[tiab]

OR "generative AI"[tiab]

OR "generative pretrained transformer*"[tiab]

OR "generative pre-trained transformer*"[tiab]

OR GPT[tiab]

OR GPT-3[tiab]

OR GPT-4[tiab]

OR GPT-4V[tiab]

OR ChatGPT[tiab]

OR Gemini[tiab]

OR "Med-PaLM"[tiab]

OR transformer*[tiab]

)

AND

(

"report generation"[tiab]

OR "report generat*"[tiab]

OR "generat *report*"[tiab]

OR "automated report*"[tiab]

OR "automatic report*"[tiab]

OR "preliminary report*"[tiab]

OR "preliminary interpretation*"[tiab]

OR "report draft*"[tiab]

OR "draft report*"[tiab]

OR "drafting report*"[tiab]

OR "impression generation"[tiab]

OR "diagnostic impression*"[tiab]

OR "findings-to-impression"[tiab]

OR "findings to impression"[tiab]

OR "medical report*"[tiab]

OR "radiology report*"[tiab]

OR "pathology report*"[tiab]

OR "endoscopy report*"[tiab]

OR "structured report*"[tiab]

OR "image caption*"[tiab]

OR "image-to-text"[tiab]

OR "image to text"[tiab]

OR "caption generation"[tiab]

OR "medical caption*"[tiab]

)

AND

(

"Diagnostic Imaging"[Mesh]

OR "Radiology"[Mesh]

OR "Radiography"[Mesh]

OR "Tomography, X-Ray Computed"[Mesh]

OR "Magnetic Resonance Imaging"[Mesh]

OR "Ultrasonography"[Mesh]

OR "Mammography"[Mesh]

OR "Positron-Emission Tomography"[Mesh]

OR "Pathology"[Mesh]

OR "Endoscopy"[Mesh]

OR "Colonoscopy"[Mesh]

OR radiolog*[tiab]

OR imaging[tiab]

OR radiograph*[tiab]

OR xray[tiab]

OR x-ray[tiab]

OR "x ray"[tiab]

OR "chest x-ray"[tiab]

OR "chest xray"[tiab]

OR CXR[tiab]

OR CT[tiab]

OR "computed tomography"[tiab]

OR MRI[tiab]

OR "magnetic resonance"[tiab]

OR ultrasound[tiab]

OR ultrason*[tiab]

OR sonograph*[tiab]

OR mammograph*[tiab]

OR PET[tiab]

OR "nuclear medicine"[tiab]

OR pathology[tiab]

OR histopatholog*[tiab]

OR cytolog*[tiab]

OR "whole slide"[tiab]

OR "whole-slide"[tiab]

OR WSI[tiab]

OR endoscop*[tiab]

OR colonoscop*[tiab]

OR gastroscop*[tiab]

OR bronchoscop*[tiab]

)

NOT

(

review[pt]

OR editorial[pt]

OR letter[pt]

OR comment[pt]

OR protocol[ti]

)

```

# Embase

Filters applied or used when available:

- Date: January 1, 2016, to May 2026 search date
- Language: English
- Source type/document type: article; remove conference abstracts after preserving raw count when supported by the database interface

```text

(

'large language model'/exp

OR 'artificial intelligence'/exp

OR 'natural language processing'/exp

OR 'machine learning'/exp

OR 'large language model*':ti,ab,kw

OR 'large-language model*':ti,ab,kw

OR llm:ti,ab,kw

OR llms:ti,ab,kw

OR 'multimodal large language model*':ti,ab,kw

OR 'multi-modal large language model*':ti,ab,kw

OR mllm:ti,ab,kw

OR mllms:ti,ab,kw

OR 'vision-language model*':ti,ab,kw

OR 'vision language model*':ti,ab,kw

OR 'large vision-language model*':ti,ab,kw

OR 'large vision language model*':ti,ab,kw

OR vlm:ti,ab,kw

OR vlms:ti,ab,kw

OR lvlm:ti,ab,kw

OR lvlms:ti,ab,kw

OR 'foundation model*':ti,ab,kw

OR 'generative artificial intelligence':ti,ab,kw

OR 'generative ai':ti,ab,kw

OR 'generative pretrained transformer*':ti,ab,kw

OR 'generative pre-trained transformer*':ti,ab,kw

OR gpt:ti,ab,kw

OR 'gpt-3':ti,ab,kw

OR 'gpt-4':ti,ab,kw

OR 'gpt-4v':ti,ab,kw

OR chatgpt:ti,ab,kw

OR gemini:ti,ab,kw

OR 'med-palm':ti,ab,kw

OR transformer*:ti,ab,kw

)

AND

(

'report generation':ti,ab,kw

OR 'report generat*':ti,ab,kw

OR 'generat *report*':ti,ab,kw

OR 'automated report*':ti,ab,kw

OR 'automatic report*':ti,ab,kw

OR 'preliminary report*':ti,ab,kw

OR 'preliminary interpretation*':ti,ab,kw

OR 'report draft*':ti,ab,kw

OR 'draft report*':ti,ab,kw

OR 'drafting report*':ti,ab,kw

OR 'impression generation':ti,ab,kw

OR 'diagnostic impression*':ti,ab,kw

OR 'findings-to-impression':ti,ab,kw

OR 'findings to impression':ti,ab,kw

OR 'medical report*':ti,ab,kw

OR 'radiology report*':ti,ab,kw

OR 'pathology report*':ti,ab,kw

OR 'endoscopy report*':ti,ab,kw

OR 'structured report*':ti,ab,kw

OR 'image caption*':ti,ab,kw

OR 'image-to-text':ti,ab,kw

OR 'image to text':ti,ab,kw

OR 'caption generation':ti,ab,kw

OR 'medical caption*':ti,ab,kw

)

AND

(

'diagnostic imaging'/exp

OR 'radiology'/exp

OR 'radiography'/exp

OR 'computer assisted tomography'/exp

OR 'nuclear magnetic resonance imaging'/exp

OR 'echography'/exp

OR 'mammography'/exp

OR 'positron emission tomography'/exp

OR 'pathology'/exp

OR 'endoscopy'/exp

OR 'colonoscopy'/exp

OR radiolog*:ti,ab,kw

OR imaging:ti,ab,kw

OR radiograph*:ti,ab,kw

OR xray:ti,ab,kw

OR 'x-ray':ti,ab,kw

OR 'x ray':ti,ab,kw

OR 'chest x-ray':ti,ab,kw

OR 'chest xray':ti,ab,kw

OR cxr:ti,ab,kw

OR ct:ti,ab,kw

OR 'computed tomography':ti,ab,kw

OR mri:ti,ab,kw

OR 'magnetic resonance':ti,ab,kw

OR ultrasound:ti,ab,kw

OR ultrason*:ti,ab,kw

OR sonograph*:ti,ab,kw

OR mammograph*:ti,ab,kw

OR pet:ti,ab,kw

OR 'nuclear medicine':ti,ab,kw

OR pathology:ti,ab,kw

OR histopatholog*:ti,ab,kw

OR cytolog*:ti,ab,kw

OR 'whole slide':ti,ab,kw

OR 'whole-slide':ti,ab,kw

OR wsi:ti,ab,kw

OR endoscop*:ti,ab,kw

OR colonoscop*:ti,ab,kw

OR gastroscop*:ti,ab,kw

OR bronchoscop*:ti,ab,kw

)

NOT

(

'review'/it

OR 'editorial'/it

OR 'letter'/it

OR 'conference abstract'/it

OR protocol:ti

)

```

# Web of Science Core Collection

Filters applied or used when available:

- Timespan: January 1, 2016, to May 2026 search date
- Language: English
- Document type: Article; exclude Review Article, Editorial Material, Letter, Meeting Abstract after preserving raw count when supported by the database interface

```text

TS=(

(

"large language model*" OR "large-language model*" OR LLM OR LLMs

OR "multimodal large language model*" OR "multi-modal large language model*" OR MLLM OR MLLMs

OR "vision-language model*" OR "vision language model*" OR "large vision-language model*" OR "large vision language model*"

OR VLM OR VLMs OR LVLM OR LVLMs

OR "foundation model*" OR "generative artificial intelligence" OR "generative AI"

OR "generative pretrained transformer*" OR "generative pre-trained transformer*"

OR GPT OR "GPT-3" OR "GPT-4" OR "GPT-4V" OR ChatGPT OR Gemini OR "Med-PaLM" OR transformer*

)

AND

(

"report generation" OR "report generat*" OR "generat* report*"

OR "automated report*" OR "automatic report*" OR "preliminary report*" OR "preliminary interpretation*"

OR "report draft*" OR "draft report*" OR "drafting report*"

OR "impression generation" OR "diagnostic impression*" OR "findings-to-impression" OR "findings to impression"

OR "medical report*" OR "radiology report*" OR "pathology report*" OR "endoscopy report*" OR "structured report*"

OR "image caption*" OR "image-to-text" OR "image to text" OR "caption generation" OR "medical caption*"

)

AND

(

radiolog *OR imaging OR radiograph* OR xray OR "x-ray" OR "x ray" OR "chest x-ray" OR "chest xray" OR CXR

OR CT OR "computed tomography" OR MRI OR "magnetic resonance"

OR ultrasound OR ultrason *OR sonograph* OR mammograph* OR PET OR "nuclear medicine"

OR pathology OR histopatholog *OR cytolog* OR "whole slide" OR "whole-slide" OR WSI

OR endoscop *OR colonoscop* OR gastroscop *OR bronchoscop*

)

)

```

# Scopus

Filters applied or used when available:

- Date: January 1, 2016, to May 2026 search date
- Language: English
- Document type: Article; exclude reviews, editorials, letters, and conference papers after preserving raw count when supported by the database interface

```text

TITLE-ABS-KEY(

(

"large language model*" OR "large-language model*" OR LLM OR LLMs

OR "multimodal large language model*" OR "multi-modal large language model*" OR MLLM OR MLLMs

OR "vision-language model*" OR "vision language model*" OR "large vision-language model*" OR "large vision language model*"

OR VLM OR VLMs OR LVLM OR LVLMs

OR "foundation model*" OR "generative artificial intelligence" OR "generative AI"

OR "generative pretrained transformer*" OR "generative pre-trained transformer*"

OR GPT OR "GPT-3" OR "GPT-4" OR "GPT-4V" OR ChatGPT OR Gemini OR "Med-PaLM" OR transformer*

)

AND

(

"report generation" OR "report generat*" OR "generat* report*"

OR "automated report*" OR "automatic report*" OR "preliminary report*" OR "preliminary interpretation*"

OR "report draft*" OR "draft report*" OR "drafting report*"

OR "impression generation" OR "diagnostic impression*" OR "findings-to-impression" OR "findings to impression"

OR "medical report*" OR "radiology report*" OR "pathology report*" OR "endoscopy report*" OR "structured report*"

OR "image caption*" OR "image-to-text" OR "image to text" OR "caption generation" OR "medical caption*"

)

AND

(

radiolog *OR imaging OR radiograph* OR xray OR "x-ray" OR "x ray" OR "chest x-ray" OR "chest xray" OR CXR

OR CT OR "computed tomography" OR MRI OR "magnetic resonance"

OR ultrasound OR ultrason *OR sonograph* OR mammograph* OR PET OR "nuclear medicine"

OR pathology OR histopatholog *OR cytolog* OR "whole slide" OR "whole-slide" OR WSI

OR endoscop *OR colonoscop* OR gastroscop *OR bronchoscop*

)

)

AND PUBYEAR > 2015

AND NOT DOCTYPE(re)

AND NOT DOCTYPE(ed)

AND NOT DOCTYPE(le)

AND NOT DOCTYPE(cp)

```

# Cochrane Library

Use in this review: supplementary database for trials, reviews with reference leads, and comparative workflow studies. All text fields were searched when supported by the interface.

```text

(

"large language model*" OR LLM OR LLMs OR "multimodal large language model*" OR MLLM

OR "vision-language model*" OR "vision language model*" OR VLM OR "foundation model*"

OR "generative artificial intelligence" OR "generative AI" OR GPT OR ChatGPT OR Gemini

)

AND

(

"report generation" OR "report generat*" OR "generat* report*" OR "report draft*" OR "draft report*"

OR "impression generation" OR "diagnostic impression*" OR "radiology report*" OR "medical report*"

OR "pathology report*" OR "endoscopy report*" OR "image caption*" OR "caption generation"

)

AND

(

radiology OR imaging OR radiograph* OR x-ray OR xray OR CT OR MRI OR ultrasound OR pathology

OR endoscopy OR colonoscopy OR bronchoscopy

)

```

# Backward Citation Searching

For every included study after the updated database search:

1. Screen all references in the article and relevant supplementary material.

2. Record each potentially eligible citation in the citation-search screening log.

3. Deduplicate against database records using DOI, PMID, title, and year.

4. Screen title/abstract and full text according to the same eligibility criteria.

# Forward Citation Searching

For every included study after the updated database search:

1. Use Google Scholar, Web of Science, or Scopus cited-reference tools when available.

2. Record date searched, platform, number of citing records, and number moved to screening.

3. Deduplicate against database and backward-search records.

4. Screen and document exclusions with the same reason hierarchy as the database search.

# Search Documentation Elements

The following elements were recorded for each source where applicable:

- Database/interface name
- Platform/vendor
- Date searched
- Full date range covered
- Exact final search string
- Limits or filters applied
- Number of records retrieved before deduplication
- Export format
- Deduplication method and software
- Any deviations from planned search strategy
