# Supplementary material for: Effectiveness, Safety, and Workflow Burden of Large Language Model–Based Medical Report Generation: Systematic Review
Source: J Med Internet Res. 2026 Sep 9;28:e97007. doi: 10.2196/97007 (PMC13557449; doi:10.2196/97007)
Supplement: Supplementary file 2 [file jmir-v28-e97007-s002.docx]

**Supplementary Evidence Tables**

Complete evidence appendix. Articles are identified using manuscript citation labels or formal author-year study citations; screening and extraction codes are omitted.

**Contents**

This appendix consolidates study-level evidence, risk-of-bias judgments, outcome-level extraction records, and synthesis decisions for the systematic review.

| **Section** | **Navigation note** |
| --- | --- |
| Supplementary Table S1 | Included studies in the full evidence base |
| Supplementary Table S2 | Full-text exclusions with reasons |
| Supplementary Table S3A | Risk-of-bias framework domains |
| Supplementary Table S3B | Overall risk-of-bias judgments at the study level |
| Supplementary Table S4 | Outcome-level extraction summary |
| Supplementary Table S5 | Targeted quantitative results and pooling decisions |
| Supplementary Table S6 | Reasons clinically relevant outcomes could not be pooled |

**Supplementary Table S1. Included Studies in the Full Evidence Base**

| **Study citation** | **Article details** | **Evidence category** | **Risk-of-bias judgment and framework** | **Synthesis or outcome note** |
| --- | --- | --- | --- | --- |
| Zhong et al, 2026 [4] | ChatRadio-Valuer: A Chat Large Language Model for Generalizable Radiology Impression Generation on Multi-Institution and Multi-System Data. (2026; IEEE Trans Biomed Eng) | Retrospective/technical validation; Mixed modality; Findings-to-impression; Post-editing/structuring | Moderate risk; custom AI validation RoB domains + CLAIM adjunct | Reporting time |
| Yeasin M et al, 2024 [4] | Auto-Rad: End-to-End Report Generation from Lumber Spine MRI Using Vision-Language Model. (2024; J Clin Med) | Retrospective/technical validation; MRI; Image-to-report; Autonomous | Moderate risk; custom AI validation RoB domains + CLAIM adjunct | No extractable metric specified. |
| Lee et al, 2024 [6] | Comparative Analysis of M4CXR, an LLM-Based Chest X-Ray Report Generation Model, and ChatGPT in Radiological Interpretation. (2024; J Clin Med) | Nonrandomized reader/workflow study; Chest X-ray; Image-to-report; Autonomous | Serious risk; ROBINS-I + CLAIM adjunct | No extractable metric specified. |
| Chen et al, 2025 [7] | Large Language Model With Region-Guided Referring and Grounding for CT Report Generation. (2025; IEEE Trans Med Imaging) | Retrospective/technical validation; CT/CTA/PET-CT; Image-to-report; Autonomous | Moderate risk; custom AI validation RoB domains + CLAIM adjunct | No extractable metric specified. |
| Massimi et al, 2026 [8] | Large Language Model-Driven Analysis and Report Generation of Endoscopy Videos-A Pilot Study. (2026; Dig Endosc) | Retrospective/technical validation; Endoscopy; Image-to-report; Autonomous | High risk; custom AI validation RoB domains + CLAIM adjunct | No extractable metric specified. |
| Zhang et al, 2026 [9] | A Fine-tuning Multimodal Large Language Model for Endoscopic Report Generation. (2026; BIOMEDICAL SIGNAL PROCESSING AND CONTROL) | Retrospective/technical validation; Endoscopy; Image-to-report; Autonomous | High risk; custom AI validation RoB domains + CLAIM adjunct | BLEU; ROUGE; CIDEr; BERTScore |
| Joyson et al, 2026 [10] | A multi-modal fusion-based deep learning with finetuned LLaMA 3 for lung disease diagnosis using PACS radiology reports. (; ) | Retrospective/technical validation; Mixed modality; Image-to-report; Autonomous | High risk; custom AI validation RoB domains + CLAIM adjunct | BLEU |
| Liu et al, 2025 [11] | MetaGP: A Generative Foundation Model Integrating Electronic Health Records and Multimodal Imaging for Addressing Unmet Clinical Needs. (; ) | Retrospective/technical validation; Chest X-ray; Image-to-report; Autonomous | High risk; custom AI validation RoB domains + CLAIM adjunct | No extractable metric specified. |
| Dasanayaka et al, 2025 [12] | Multimodal AI and Large Language Models for Orthopantomography Radiology Report Generation and Q&A. (2025; APPLIED SYSTEM INNOVATION) | Retrospective/technical validation; Chest X-ray; Image-to-report; Autonomous | High risk; custom AI validation RoB domains + CLAIM adjunct | No extractable metric specified. |
| Helmy et al, 2026 [13] | SPINE: Segmentation-guided Processing and Integration of Multimodal Spinal MRI for Natural-Language Enhanced Report Generation. (2026; APPLIED ARTIFICIAL INTELLIGENCE) | Retrospective/technical validation; MRI; Image-to-report; Autonomous | High risk; custom AI validation RoB domains + CLAIM adjunct | METEOR; Clinical accuracy score; Narrative quality score |
| Li et al, 2025 [14] | Ultrasound report generation with fuzzy knowledge and multi-modal large language model. (2025; EXPERT SYSTEMS WITH APPLICATIONS) | Retrospective/technical validation; Ultrasound; Image-to-report; Autonomous | High risk; custom AI validation RoB domains + CLAIM adjunct | No extractable metric specified. |
| Wang et al, 2023 [15] | R2GenGPT: Radiology Report Generation with frozen LLMs. (2023; META-RADIOLOGY) | Retrospective/technical validation; Mixed modality; Image-to-report; Autonomous | High risk; custom AI validation RoB domains + CLAIM adjunct | No extractable metric specified. |
| Liu et al, 2024 [16] | A systematic evaluation of GPT-4V's multimodal capability for chest X-ray image analysis. (2024; META-RADIOLOGY) | Nonrandomized reader/workflow study; Chest X-ray; Image-to-report; Autonomous | Serious risk; ROBINS-I + CLAIM adjunct | Findings-impression discrepancy rate |
| Kim et al, 2025 [17] | Enhancing Structured Pathology Report Generation With Foundation Model and Modular Design. (2025; IEEE ACCESS) | Retrospective/technical validation; Pathology whole slide imaging; Image-to-report; Autonomous | Moderate risk; custom AI validation RoB domains + CLAIM adjunct | BLEU; ROUGE |
| Yan et al, 2025 [18] | Multimodal Medical Image Analysis: Integrating LLM and RAG Deep Learning Strategies. (2025; JOURNAL OF ADVANCES IN INFORMATION TECHNOLOGY) | Retrospective/technical validation; Pathology whole slide imaging; Image-to-report; Autonomous | High risk; custom AI validation RoB domains + CLAIM adjunct | No extractable metric specified. |
| Gholipour et al, 2026 [39] | Trust but verify: Image-aware evaluation of radiology report generators. (2026; MACHINE LEARNING WITH APPLICATIONS) | Nonrandomized reader/workflow study; Chest X-ray; Image-to-report; Autonomous | Serious risk; ROBINS-I + CLAIM adjunct | MCSE; image-aware semantic consistency |
| Wang et al, 2026 [46] | Activating Associative Disease-Aware Vision Token Memory for LLM-Based X-Ray Report Generation. (2026; IEEE Trans Med Imaging) | Retrospective/technical validation; Chest X-ray; Image-to-report; Autonomous | Moderate risk; custom AI validation RoB domains + CLAIM adjunct | No extractable metric specified. |
| Noh et al, 2026 [47] | Hybrid framework for lesion-aware, clinically coherent chest X-ray report generation using contrastive learning and large language models. (2026; Sci Rep) | Retrospective/technical validation; Chest X-ray; Image-to-report; Autonomous | High risk; custom AI validation RoB domains + CLAIM adjunct | METEOR; ROUGE; GREEN; RadGraph F1; narrative human consistency score |
| Han et al, 2026 [48] | Dynamic feature fusion guiding and multimodal large language model refining for medical image report generation. (2026; EXPERT SYSTEMS WITH APPLICATIONS) | Retrospective/technical validation; Chest X-ray; Image-to-report; Human-supervised | High risk; custom AI validation RoB domains + CLAIM adjunct | BLEU; ROUGE; CIDEr |
| Jia et al, 2026 [49] | Semantic Feedback-Based RAG for Radiology Report Generation. (2026; BIG DATA MINING AND ANALYTICS) | Retrospective/technical validation; Chest X-ray; Image-to-report; Autonomous | High risk; custom AI validation RoB domains + CLAIM adjunct | BLEU; ROUGE |
| Wang et al, 2026 [50] | R2GenCSR: Mining Contextual and Residual Information for LLMs-based Radiology Report Generation. (2026; IEEE J Biomed Health Inform) | Retrospective/technical validation; Chest X-ray; Image-to-report; Autonomous | High risk; custom AI validation RoB domains + CLAIM adjunct | No extractable metric specified. |
| Hong et al, 2025 [51] | Diagnostic Accuracy and Clinical Value of a Domain-specific Multimodal Generative AI Model for Chest Radiograph Report Generation. (2025; Radiology) | Retrospective/technical validation; Chest X-ray; Image-to-report; Autonomous | Moderate risk; custom AI validation RoB domains + CLAIM adjunct | Expert acceptance rate; Commission error rate; Omission error rate |
| Tanno et al, 2025 [52] | Collaboration between clinicians and vision-language models in radiology report generation. (2025; Nat Med) | Retrospective/technical validation; Chest X-ray; Image-to-report; Human-supervised | Moderate risk; custom AI validation RoB domains + CLAIM adjunct | Clinically significant error rate; Blinded expert preference |
| Li et al, 2025 [53] | Towards a holistic framework for multimodal LLM in 3D brain CT radiology report generation. (2025; Nat Commun) | Retrospective/technical validation; CT/CTA/PET-CT; Image-to-report; Autonomous | Moderate risk; custom AI validation RoB domains + CLAIM adjunct | Blinded expert preference; forte f1 |
| Luo et al, 2026 [54] | Towards Automated Reporting: A Bronchoscopy Report Dataset for Enhancing Multimodality Large Language Models. (2026; Sci Data) | Retrospective/technical validation; Endoscopy; Image-to-report; Autonomous | Moderate risk; custom AI validation RoB domains + CLAIM adjunct | Expert acceptance rate; caption accuracy |
| Ayaz et al, 2025 [55] | MedVLM: Medical Vision-Language Model for Consumer Devices. (2025; IEEE CONSUMER ELECTRONICS MAGAZINE) | Retrospective/technical validation; Mixed modality; Image-to-report; Autonomous | High risk; custom AI validation RoB domains + CLAIM adjunct | Expert quality rating |
| Voinea et al, 2024 [56] | GPT-Driven Radiology Report Generation with Fine-Tuned Llama 3. (2024; Bioengineering (Basel)) | Retrospective/technical validation; Mixed modality; Image-to-report; Autonomous | High risk; custom AI validation RoB domains + CLAIM adjunct | No extractable metric specified. |
| Wang et al, 2026 [57] | Evaluation of large language models for diagnostic impression generation from brain MRI report findings: a multicenter benchmark and reader study. (2026; NPJ Digit Med) | Nonrandomized reader/workflow study; MRI; Findings-to-impression; Post-editing/structuring | Serious risk; ROBINS-I + CLAIM adjunct | Reporting time |
| Serapio et al, 2024 [58] | An open-source fine-tuned large language model for radiological impression generation: a multi-reader performance study. (2024; BMC Med Imaging) | Nonrandomized reader/workflow study; Mixed modality; Findings-to-impression; Human-supervised | Serious risk; ROBINS-I + CLAIM adjunct | Reporting time; Edit distance; ROUGE |
| Park J et al, 2026 [47] | Prospective evaluation of artificial intelligence (AI) in lumbar spine magnetic resonance imaging (MRI) workflow: from deep learning (DL)-enhanced accelerated acquisition to simultaneous vision-language model (VLM)-based automated report generation. (2026; Eur J Radiol) | Prospective or real-world workflow/validation study; MRI; Report drafting/structuring; Human-supervised | Serious risk; ROBINS-I + CLAIM adjunct | Reporting time |
| Soleimani et al, 2024 [60] | Practical Evaluation of ChatGPT Performance for Radiology Report Generation. (2024; Acad Radiol) | Nonrandomized reader/workflow study; Chest X-ray; Report drafting/structuring; Post-editing/structuring | Serious risk; ROBINS-I + CLAIM adjunct | Reporting time |
| Azhar et al, 2026 [61] | Semiautomated breast Ultrasound report generation using multimodal large language models and deep learning. (2026; Front Med (Lausanne)) | Retrospective/technical validation; Ultrasound; Image-to-report; Human-supervised | High risk; custom AI validation RoB domains + CLAIM adjunct | Reporting time |
| Chen K et al, 2025 [50] | The Potential of Gemini and GPTs for Structured Report Generation based on Free-Text (18)F-FDG PET/CT Breast Cancer Reports. (2025; Acad Radiol) | Retrospective/technical validation; CT/CTA/PET-CT; Report drafting/structuring; Human-supervised | High risk; custom AI validation RoB domains + CLAIM adjunct | Structured extraction accuracy; progress-decision accuracy |
| Bosbach et al, 2026 [63] | Conceptual proposal for LLM-generated FDG PET/CT follow-up reports in melanoma: a pilot study on model stability and blinded expert evaluation. (2026; Frontiers in Nuclear Medicine) | Nonrandomized reader/workflow study; CT/CTA/PET-CT; Report drafting/structuring; Autonomous | Serious risk; ROBINS-I + CLAIM/custom AI reporting adjunct | Blinded expert evaluation; small PET/CT follow-up sample. |
| Lorusso et al, 2026 [64] | Comparative evaluation of large language models for generating CAD-RADS 2.0-compliant diagnostic conclusions in cardiac CT reports. (2026; Insights into Imaging) | Nonrandomized reader/workflow study; CT/CTA/PET-CT; Impression/conclusion generation; Autonomous | High risk; ROBINS-I + CLAIM/custom AI reporting adjunct | CAD-RADS conclusion; recommendation accuracy; execution time. |
| Zanardo et al, 2026 [65] | Can AI write reports like a radiologist? A blinded evaluation of large language model-generated lumbar spine MRI reports. (2026; European Radiology Experimental) | Nonrandomized reader/workflow study; MRI; Report drafting/structuring; Autonomous | High risk; ROBINS-I + CLAIM/custom AI reporting adjunct | Evidence-base inclusion; RoB summarized. |
| Tran et al, 2025 [66] | Generating dermatopathology reports from gigapixel whole slide images with HistoGPT. (2025; Nature Communications) | Retrospective/technical validation; Pathology whole slide imaging; Report generation from images; Autonomous | Moderate risk; custom AI validation RoB domains + CLAIM adjunct | Evidence-base inclusion; RoB summarized. |
| Raminedi et al, 2024 [67] | Multi-modal transformer architecture for medical image analysis and automated report generation. (2024; Scientific Reports) | Retrospective/technical validation; Chest x-ray; Report generation from images; Autonomous | High risk; custom AI validation RoB domains + CLAIM adjunct | Evidence-base inclusion; RoB summarized. |
| Liu et al, 2026 [68] | SpineVLM: A Markdown-Guided Structured Fine-Tuning Framework for Spine X-ray Report Generation. (2026; IEEE journal of biomedical and health informatics) | Nonrandomized reader/workflow study; Other x-ray or radiography; Report generation from images; Human-supervised | High risk; ROBINS-I + CLAIM/custom AI reporting adjunct | Evidence-base inclusion; RoB summarized. |
| Ye et al, 2026 [69] | Report Generation System for Slit-Lamp Image Interpretation Using Vision-Language Models (2026; Ophthalmology and Therapy) | Retrospective/technical validation; Ophthalmic imaging; Report generation from images; Autonomous | High risk; custom AI validation RoB domains + CLAIM adjunct | Evidence-base inclusion; RoB summarized. |
| Tan et al, 2026 [70] | Large language model-assisted radiology reporting in a single-radiologist implementation: a retrospective cohort study interpreted through a UTAUT lens. (2026; Abdominal radiology (New York)) | Prospective/real-world workflow; Mixed modality; Report drafting/structuring; Human-supervised | Serious risk; ROBINS-I + CLAIM/custom AI reporting adjunct | Evidence-base inclusion; RoB summarized. |
| Li et al, 2026 [71] | Fine-Tuned Large Language Model for Automated Radiology Impression Generation: A Multicenter Evaluation. (2026; Radiology. Artificial intelligence) | Nonrandomized reader/workflow study; Mixed modality; Impression/conclusion generation; Human-supervised | Moderate risk; ROBINS-I + CLAIM/custom AI reporting adjunct | Evidence-base inclusion; RoB summarized. |
| Barzegar et al, 2026 [72] | Enhancing chest X-ray report generation with pathology-guided prompts and vision-language shortcut bias mitigation (2026; Biomedical Signal Processing and Control) | Retrospective/technical validation; Chest x-ray; Report generation from images; Autonomous | High risk; custom AI validation RoB domains + CLAIM adjunct | Evidence-base inclusion; RoB summarized. |
| Allaberdiev et al, 2026 [73] | Chestxgen: Dynamic Memory-Augmented Vision-Language Transformer with Context-Aware Gating for Radiology Report Generation (2026; Journal of Artificial Intelligence and Soft Computing Research) | Retrospective/technical validation; Chest x-ray; Report generation from images; Autonomous | High risk; custom AI validation RoB domains + CLAIM adjunct | Evidence-base inclusion; RoB summarized. |
| Lin et al, 2026 [74] | Automatic Speech Recognition and Large Language Models for Multilingual Pathology Report Generation: Proof-of-Concept Study. (2026; JMIR formative research) | Retrospective/technical validation; Pathology report or whole slide imaging; Report drafting/structuring; Human-supervised | Serious risk; custom AI validation RoB domains + CLAIM adjunct | Pathology dictation/transcript-to-report; serious RoB. |
| Khatoon et al, 2026 [75] | Automated Radiological Report Generation from Breast Ultrasound Images Using Vision and Language Transformers. (2026; Journal of imaging) | Retrospective/technical validation; Ultrasound; Report generation from images; Autonomous | High risk; custom AI validation RoB domains + CLAIM adjunct | Evidence-base inclusion; RoB summarized. |
| Park et al, 2026 [76] | A robust vision language model for molecular status prediction and radiology report generation in adult-type diffuse gliomas. (2026; NPJ digital medicine) | Retrospective/technical validation; MRI; Report generation from images; Autonomous | Moderate risk; custom AI validation RoB domains + CLAIM adjunct | Evidence-base inclusion; RoB summarized. |
| Hossain et al, 2025 [77] | Privacy-Preserving Chest X-Ray Report Generation via Multimodal Federated Learning with ViT and GPT2 (2025; Biomedical Materials and Devices) | Retrospective/technical validation; Chest x-ray; Report generation from images; Autonomous | High risk; custom AI validation RoB domains + CLAIM adjunct | Evidence-base inclusion; RoB summarized. |
| Hosseini et al, 2025 [78] | M3: multimodal artificial intelligence for medical report generation and visual question answering from 3D abdominal CT scans. (2025; BJR artificial intelligence) | Retrospective/technical validation; CT/CTA/PET-CT; Report generation from images; Autonomous | High risk; custom AI validation RoB domains + CLAIM adjunct | Evidence-base inclusion; RoB summarized. |
| Wang et al, 2024 [79] | Enhancing Diagnostic Accuracy and Efficiency with GPT-4-Generated Structured Reports: A Comprehensive Study (2024; Journal of Medical and Biological Engineering) | Nonrandomized reader/workflow study; Ultrasound; Report drafting/structuring; Editing/structuring support | High risk; ROBINS-I + CLAIM/custom AI reporting adjunct | Evidence-base inclusion; RoB summarized. |
| Zhang et al, 2024 [80] | Constructing a Large Language Model to Generate Impressions from Findings in Radiology Reports. (2024; Radiology) | Nonrandomized reader/workflow study; Mixed modality; Impression/conclusion generation; Editing/structuring support | High risk; ROBINS-I + CLAIM/custom AI reporting adjunct | Evidence-base inclusion; RoB summarized. |
| Sun et al, 2023 [81] | Evaluating GPT4 on Impressions Generation in Radiology Reports. (2023; Radiology) | Nonrandomized reader/workflow study; Chest x-ray; Impression/conclusion generation; Autonomous | High risk; ROBINS-I + CLAIM/custom AI reporting adjunct | Evidence-base inclusion; RoB summarized. |
| Huang et al, 2023 [82] | Generative Artificial Intelligence for Chest Radiograph Interpretation in the Emergency Department. (2023; JAMA network open) | Nonrandomized reader/workflow study; Chest x-ray; Report generation from images; Autonomous | High risk; ROBINS-I + CLAIM/custom AI reporting adjunct | Evidence-base inclusion; RoB summarized. |
| Mallio et al, 2024 [83] | ChatGPT in radiology structured reporting: analysis of ChatGPT-3.5 Turbo and GPT-4 in reducing word count and recalling findings. (2024; Quantitative imaging in medicine and surgery) | Retrospective/technical validation; Mixed modality; Report drafting/structuring; Editing/structuring support | High risk; custom AI validation RoB domains + CLAIM adjunct | Evidence-base inclusion; RoB summarized. |
| Stephan et al, 2024 [84] | AI in Dental Radiology-Improving the Efficiency of Reporting With ChatGPT: Comparative Study. (2024; Journal of medical Internet research) | Nonrandomized reader/workflow study; Other x-ray or radiography; Report drafting/structuring; Human-supervised | High risk; ROBINS-I + CLAIM/custom AI reporting adjunct | Evidence-base inclusion; RoB summarized. |
| Kim et al, 2025 [85] | Utility of Multimodal Large Language Models in Analyzing Chest X-Rays with Incomplete Contextual Information. (2025; Healthcare informatics research) | Nonrandomized reader/workflow study; Chest x-ray; Impression/conclusion generation; Autonomous | High risk; ROBINS-I + CLAIM/custom AI reporting adjunct | Evidence-base inclusion; RoB summarized. |
| Di et al, 2025 [86] | Structured Transformation of Unstructured Prostate MRI Reports Using Large Language Models. (2025; Tomography (Ann Arbor, Mich.)) | Retrospective/technical validation; MRI; Report drafting/structuring; Editing/structuring support | High risk; custom AI validation RoB domains + CLAIM adjunct | Evidence-base inclusion; RoB summarized. |
| Xie et al, 2025 [87] | Large language models for efficient whole-organ MRI score-based reports and categorization in knee osteoarthritis. (2025; Insights into imaging) | Nonrandomized reader/workflow study; MRI; Report drafting/structuring; Editing/structuring support | High risk; ROBINS-I + CLAIM/custom AI reporting adjunct | Evidence-base inclusion; RoB summarized. |
| Shao et al, 2025 [88] | Generative artificial intelligence for fundus fluorescein angiography interpretation and human expert evaluation (2025; npj Digital Medicine) | Nonrandomized reader/workflow study; Ophthalmic imaging; Report generation from images; Autonomous | Moderate risk; ROBINS-I + CLAIM/custom AI reporting adjunct | Evidence-base inclusion; RoB summarized. |
| Gupta et al, 2025 [89] | Enhancing Radiological Reporting in Head and Neck Cancer: Converting Free-Text CT Scan Reports to Structured Reports Using Large Language Models. (2025; The Indian journal of radiology & imaging) | Retrospective/technical validation; CT/CTA/PET-CT; Report drafting/structuring; Editing/structuring support | High risk; custom AI validation RoB domains + CLAIM adjunct | Evidence-base inclusion; RoB summarized. |
| Lee et al, 2025 [90] | CXR-LLaVA: a multimodal large language model for interpreting chest X-ray images. (2025; European radiology) | Retrospective/technical validation; Chest x-ray; Report generation from images; Autonomous | High risk; custom AI validation RoB domains + CLAIM adjunct | Evidence-base inclusion; RoB summarized. |
| Pesapane et al, 2025 [91] | A preliminary investigation into the potential, pitfalls, and limitations of large language models for mammography interpretation (2025; Discover Oncology) | Retrospective/technical validation; Other x-ray or radiography; Report generation from images; Autonomous | High risk; custom AI validation RoB domains + CLAIM adjunct | Evidence-base inclusion; RoB summarized. |
| Hartsock et al, 2026 [92] | Improving Radiology Report Conciseness and Structure via Local Large Language Models. (2026; Journal of imaging informatics in medicine) | Retrospective/technical validation; Mixed modality; Report drafting/structuring; Editing/structuring support | High risk; custom AI validation RoB domains + CLAIM adjunct | Evidence-base inclusion; RoB summarized. |
| Silbergleit et al, 2025 [93] | ChatGPT vs Gemini: Comparative Accuracy and Efficiency in CAD-RADS Score Assignment from Radiology Reports. (2025; Journal of imaging informatics in medicine) | Nonrandomized reader/workflow study; CT/CTA/PET-CT; Impression/conclusion generation; Editing/structuring support | High risk; ROBINS-I + CLAIM/custom AI reporting adjunct | CAD-RADS score assignment; structured conclusion scope. |
| Adams et al, 2023 [94] | Leveraging GPT-4 for Post Hoc Transformation of Free-text Radiology Reports into Structured Reporting: A Multilingual Feasibility Study. (2023; Radiology) | Retrospective/technical validation; Mixed modality; Report drafting/structuring; Editing/structuring support | Serious risk; custom AI validation RoB domains + CLAIM adjunct | Evidence-base inclusion; RoB summarized. |
| Jiang et al, 2024 [95] | Transforming free-text radiology reports into structured reports using ChatGPT: A study on thyroid ultrasonography. (2024; European journal of radiology) | Retrospective/technical validation; Ultrasound; Report drafting/structuring; Editing/structuring support | High risk; custom AI validation RoB domains + CLAIM adjunct | Evidence-base inclusion; RoB summarized. |
| Hasani et al, 2024 [96] | Evaluating the performance of Generative Pre-trained Transformer-4 (GPT-4) in standardizing radiology reports. (2024; European radiology) | Nonrandomized reader/workflow study; Mixed modality; Report drafting/structuring; Editing/structuring support | High risk; ROBINS-I + CLAIM/custom AI reporting adjunct | Evidence-base inclusion; RoB summarized. |
| Tsaniya et al, 2025 [97] | Medical Report Generation With Knowledge Distillation and Multi-Stage Hierarchical Attention in Vision Transformer Encoder and GPT-2 Decoder (2025; IEEE Access) | Retrospective/technical validation; Chest x-ray; Report generation from images; Autonomous | High risk; custom AI validation RoB domains + CLAIM adjunct | Evidence-base inclusion; RoB summarized. |
| Yan et al, 2025 [98] | Automated Ultrasound Diagnosis via CLIP-GPT Synergy: A Multimodal Framework for Image Classification and Report Generation (2025; IEEE Access) | Retrospective/technical validation; Ultrasound; Report generation from images; Autonomous | High risk; custom AI validation RoB domains + CLAIM adjunct | Evidence-base inclusion; RoB summarized. |
| López-Úbeda et al, 2025 [99] | Automated MRI pituitary structured reporting from free-text using a fine-tuned Llama model: a feasibility study (2025; Japanese Journal of Radiology) | Retrospective/technical validation; MRI; Report drafting/structuring; Editing/structuring support | High risk; custom AI validation RoB domains + CLAIM adjunct | Evidence-base inclusion; RoB summarized. |
| Tian et al, 2025 [100] | A Medical Multimodal Large Language Model for Pediatric Pneumonia. (2025; IEEE journal of biomedical and health informatics) | Retrospective/technical validation; Mixed modality; Report drafting/structuring; Autonomous | High risk; custom AI validation RoB domains + CLAIM adjunct | Scope restricted: report-generation components only. |
| Li et al, 2026 [101] | Towards generalizable pathology reports via a multimodal LLM with the multicenter in-context learning. (2026; Medical image analysis) | Retrospective/technical validation; Pathology whole slide imaging; Report generation from images; Autonomous | Moderate risk; custom AI validation RoB domains + CLAIM adjunct | Evidence-base inclusion; RoB summarized. |
| Hong et al, 2026 [102] | Temperature Setting of a Multimodal Generative Artificial Intelligence Model: Association With Accuracy and Quality of Artificial Intelligence-Generated Chest Radiograph Reports. (2026; AJR. American journal of roentgenology) | Nonrandomized reader/workflow study; Chest x-ray; Report generation from images; Autonomous | High risk; ROBINS-I + CLAIM/custom AI reporting adjunct | Evidence-base inclusion; RoB summarized. |
| de et al, 2026 [103] | Reporting efficiency in diagnostic imaging: Can plug-and-play general-purpose large language models outperform conventional speech recognition? (2026; European radiology) | Prospective/real-world workflow; Mixed modality; Report drafting/structuring; Human-supervised | High risk; ROBINS-I + CLAIM/custom AI reporting adjunct | Evidence-base inclusion; RoB summarized. |
| García et al, 2026 [104] | Large language model-assisted structured reporting in Radiology residents: an implementation pilot study in Emergency Radiology. (2026; SPANISH JOURNAL OF MEDICAL EDUCATION) | Prospective/real-world workflow; Mixed modality; Report drafting/structuring; Human-supervised | Serious risk; ROBINS-I + CLAIM/custom AI reporting adjunct | Evidence-base inclusion; RoB summarized. |
| Tasneem et al, 2026 [105] | From radiology findings to artificial intelligence-powered impressions: A retrospective study on the comparative performance of recent large language models (2026; Intelligent Medicine) | Nonrandomized reader/workflow study; Mixed modality; Impression/conclusion generation; Autonomous | High risk; ROBINS-I + CLAIM/custom AI reporting adjunct | Evidence-base inclusion; RoB summarized. |
| Pellegrini et al, 2026 [106] | Enhancing radiology workflows through collaborative AI-assisted chest X-ray reporting using large vision-language models: a proof-of-concept study. (2026; Insights into imaging) | Nonrandomized reader/workflow study; Chest x-ray; Report generation from images; Human-supervised | Serious risk; ROBINS-I + CLAIM/custom AI reporting adjunct | Evidence-base inclusion; RoB summarized. |
| Jiang et al, 2026 [107] | Domain specific multimodal large language model for automated Endoscopy reporting with multicenter prospective validation. (2026; NPJ digital medicine) | Prospective/real-world workflow; Endoscopy; Report generation from images; Autonomous | Moderate risk; ROBINS-I + CLAIM/custom AI reporting adjunct | Evidence-base inclusion; RoB summarized. |
| Sheng et al, 2026 [108] | BI-RADS-compliant structured mammography reporting using locally deployed large language models under privacy constraints. (2026; European radiology) | Retrospective/technical validation; Other x-ray or radiography; Report drafting/structuring; Editing/structuring support | High risk; custom AI validation RoB domains + CLAIM adjunct | Evidence-base inclusion; RoB summarized. |
| Liu et al, 2026 [109] | Teaching multimodal LLMs to comprehend 12-lead electrocardiographic images (2026; NPJ DIGITAL MEDICINE) | Retrospective/technical validation; ECG; Report generation from images; Autonomous | High risk; custom AI validation RoB domains + CLAIM adjunct | Evidence-base inclusion; RoB summarized. |
| Lee et al, 2026 [110] | Read like a radiologist: Efficient vision-language model for 3D medical imaging interpretation. (2026; Medical image analysis) | Retrospective/technical validation; CT/CTA/PET-CT; Report generation from images; Autonomous | High risk; custom AI validation RoB domains + CLAIM adjunct | Evidence-base inclusion; RoB summarized. |
| Alkhaldi A et al, 2026 [99] | MiniGPT-Med: A Unified Vision-Language Model for Radology Image Understanding (2026; Transactions on Machine Learning Research) | Retrospective/technical validation; Mixed modality; Report generation from images; Autonomous | High risk; custom AI validation RoB domains + CLAIM adjunct | Peer-reviewed venue; benchmark-dominant evidence. |
| Abdaoui et al, 2026 [112] | MedFusionT5: Cross-Modal Attention Boosts Semantic Quality and Reduces Hallucinations in Dental AI. (2026; International dental journal) | Retrospective/technical validation; Other x-ray or radiography; Report generation from images; Autonomous | High risk; custom AI validation RoB domains + CLAIM adjunct | Evidence-base inclusion; RoB summarized. |
| Mao et al, 2026 [113] | CT-Agent: a multimodal-LLM agent for 3D CT radiology question answering (2026; SCIENCE CHINA-INFORMATION SCIENCES) | Retrospective/technical validation; CT/CTA/PET-CT; Report generation from images; Autonomous | High risk; custom AI validation RoB domains + CLAIM adjunct | Scope restricted: report-generation mode only. |
| Woźnicki et al, 2025 [114] | Automatic structuring of radiology reports with on-premise open-source large language models. (2025; European radiology) | Retrospective/technical validation; Chest x-ray; Report drafting/structuring; Editing/structuring support | High risk; custom AI validation RoB domains + CLAIM adjunct | Evidence-base inclusion; RoB summarized. |
| Dong et al, 2025 [115] | Keyword-based AI assistance in the generation of radiology reports: A pilot study (2025; npj Digital Medicine) | Nonrandomized reader/workflow study; MRI; Report drafting/structuring; Human-supervised | High risk; ROBINS-I + CLAIM/custom AI reporting adjunct | Evidence-base inclusion; RoB summarized. |
| Tekdemir et al, 2026 [116] | Evaluating Large Language Models for Turkish Emergency CT Impression Drafting: Quality, Critical Omissions, and Readability. (2026; Journal of imaging informatics in medicine) | Nonrandomized reader/workflow study; CT/CTA/PET-CT; Impression/conclusion generation; Autonomous | High risk; ROBINS-I + CLAIM/custom AI reporting adjunct | Evidence-base inclusion; RoB summarized. |
| Rouhizadeh et al, 2026 [117] | The detectability paradox: bilingual medical report generation with open-weight models and the limits of human oversight. (2026; ) | Nonrandomized reader/workflow study; Mixed modality; Report drafting/structuring; Autonomous | High risk; ROBINS-I + CLAIM/custom AI reporting adjunct | Human detectability/report-generation evidence; nonrandomized. |
| Bosbach et al, 2024 [119] | Ability of ChatGPT to generate competent radiology reports for distal radius fracture by use of RSNA template items and integrated AO classifier. (; ) | Retrospective/technical validation; Mixed modality; Report drafting/structuring; Post-editing/structuring | High risk; custom AI validation RoB domains + CLAIM adjunct | Reporting time |
| Fang et al, 2025 [120] | Automated generation of chest X-ray imaging diagnostic reports by multimodal and multi granularity features fusion. (; ) | Retrospective/technical validation; Chest X-ray; Image-to-report; Autonomous | High risk; custom AI validation RoB domains + CLAIM adjunct | BLEU; ROUGE |
| Edirisinghe et al, 2025 [121] | Chest X-Ray Report Generation Using Abnormality Guided Vision Language Model. (2025; IEEE ACCESS) | Retrospective/technical validation; Chest X-ray; Image-to-report; Autonomous | High risk; custom AI validation RoB domains + CLAIM adjunct | BERTScore |
| Li et al, 2025 [122] | Context-enhanced framework for medical image report generation using multimodal contexts. (2025; KNOWLEDGE-BASED SYSTEMS) | Retrospective/technical validation; Chest X-ray; Image-to-report; Autonomous | High risk; custom AI validation RoB domains + CLAIM adjunct | No extractable metric specified. |
| Lin et al, 2026 [123] | DC-RRG: Diagnosis-centered cascaded radiology report generation. (2026; EXPERT SYSTEMS WITH APPLICATIONS) | Retrospective/technical validation; Chest X-ray; Image-to-report; Autonomous | High risk; custom AI validation RoB domains + CLAIM adjunct | No extractable metric specified. |
| Izhar et al, 2025 [124] | Engaging Preference Optimization Alignment in Large Language Model for Continual Radiology Report Generation: A Hybrid Approach. (2025; COGNITIVE COMPUTATION) | Retrospective/technical validation; Chest X-ray; Image-to-report; Autonomous | High risk; custom AI validation RoB domains + CLAIM adjunct | Commission error rate; BLEU; ROUGE |
| Leonardi et al, 2024 [125] | Enhancing radiology report generation through pre-trained language models. (2024; PROGRESS IN ARTIFICIAL INTELLIGENCE) | Retrospective/technical validation; Chest X-ray; Report drafting/structuring; Post-editing/structuring | High risk; custom AI validation RoB domains + CLAIM adjunct | BLEU; ROUGE |
| Aslam et al, 2025 [126] | Generative Artificial Intelligence for the Automated Generation of Medical Reports: A Modified Poor and Rich Optimization (MPRO)-Based Approach. (2025; TRAITEMENT DU SIGNAL) | Retrospective/technical validation; Chest X-ray; Image-to-report; Autonomous | High risk; custom AI validation RoB domains + CLAIM adjunct | BLEU; ROUGE; CIDEr |
| Deng et al, 2026 [127] | Grounded knowledge-enhanced medical vision-language pre-training for chest X-ray. (2026; BIOMEDICAL SIGNAL PROCESSING AND CONTROL) | Retrospective/technical validation; Chest X-ray; Image-to-report; Autonomous | High risk; custom AI validation RoB domains + CLAIM adjunct | No extractable metric specified. |
| Zhang et al, 2024 [118] | Potential of multimodal large language models for data mining of medical images and free-text reports. (2024; META-RADIOLOGY) | Retrospective/technical validation; Endoscopy; Report drafting/structuring; Post-editing/structuring | High risk; custom AI validation RoB domains + CLAIM adjunct | Narrative multitask report-generation performance |
| Li et al, 2025 [128] | S-RRG-Bench: Structured radiology report generation with fine-grained evaluation framework. (2025; META-RADIOLOGY) | Nonrandomized reader/workflow study; Chest X-ray; Report drafting/structuring; Autonomous | Serious risk; ROBINS-I + CLAIM adjunct | Custom structured-report score |
| Ucan et al, 2025 [129] | Turkish Chest X-Ray Report Generation Model Using the Swin Enhanced Yield Transformer (Model-SEY) Framework. (; ) | Retrospective/technical validation; Chest X-ray; Image-to-report; Autonomous | High risk; custom AI validation RoB domains + CLAIM adjunct | BLEU; ROUGE |
| Islam et al, 2025 [130] | Vision-Language Models for Automated Chest X-ray Interpretation: Leveraging ViT and GPT-2. (2025; ENGINEERING REPORTS) | Retrospective/technical validation; Chest X-ray; Image-to-report; Autonomous | High risk; custom AI validation RoB domains + CLAIM adjunct | BLEU; ROUGE; BERTScore |

**Supplementary Table S2. Full-Text Exclusions With Reasons**

| **Article assessed** | **Year and journal/source** | **Dominant exclusion reason** |
| --- | --- | --- |
| AEHRC at BioLaySumm 2025: Leveraging T5 for Lay Summarisation of Radiology Reports | 2025; Proceedings of the 24th Workshop on Biomedical Language Processing, Shared Tasks | Conference proceeding, workshop paper, or other non-journal publication |
| AI-generated neurology consultation summaries improve efficiency and reduce documentation burden in the emergency department. | 2025; Scientific Reports | Wrong report type or clinical documentation scope for the review question |
| Artificial Intelligence in Colposcopy: Evaluation of ChatGPT as a Diagnostic and Predictive Support Tool | 2025; medRxiv preprint | Preprint or other non-peer-reviewed full text |
| Artificial Intelligence in Gastrointestinal Endoscopy | 2024; Gastroenterology Clinics of North America | Review, commentary, or no eligible original data |
| Automated Structured Radiology Report Generation | 2025; Proceedings of the 63rd Annual Meeting of the Association for Computational Linguistics, Vol 1: Long Papers | Conference proceeding, workshop paper, or other non-journal publication |
| Balancing Privacy and Performance: The Impact of Facial Defacing on AI in Medical Imaging | 2025; SSRN preprint | Preprint or other non-peer-reviewed full text |
| BioVLF-T: A Temporal Framework for Radiology Report Generation using Pre-Trained Vision Language Foundational Models | 2025; Medical Imaging 2025: Clinical and Biomedical Imaging | Conference proceeding, workshop paper, or other non-journal publication |
| Blix-Ray: Chest P with Vision-Language Models and Reinforcement Learning | 2025; SSRN preprint | Unpublished or non-journal manuscript with no eligible journal publication identified |
| Bootstrapping Large Language Models for Radiology Report Generation | 2024; Thirty-Eighth AAAI Conference on Artificial Intelligence, Vol 38 No 17 | Conference proceeding, workshop paper, or other non-journal publication |
| C2RG: Parameter-efficient Adaptation of 3D Vision and Language Foundation Model for Coronary CTA Report Generation | 2024; 2024 IEEE International Conference on Bioinformatics and Biomedicine, BIBM | Conference proceeding, workshop paper, or other non-journal publication |
| Can ChatGPT write radiology reports | 2024; Chinese Journal of Academic Radiology | Review, commentary, or no eligible original data |
| CARS 2025-Computer Assisted Radiology and Surgery Proceedings of the 39th International Congress and Exhibition | 2025; International Journal of Computer Assisted Radiology and Surgery, CARS 2025 proceedings | Conference proceeding, workshop paper, or other non-journal publication |
| CBM-RAG: Demonstrating Enhanced Interpretability in Radiology Report Generation with Multi-Agent RAG and Concept Bottleneck Models | 2025; Companion of the 2025 ACM Sigchi Symposium on Engineering Interactive Computing Systems, EICS 2025 Companion | Conference proceeding, workshop paper, or other non-journal publication |
| Cervical-RG: Automated Cervical Cancer Report Generation from 3D Multi-sequence MRI via CoT-Guided Hierarchical Experts | 2026; Medical Image Computing and Computer Assisted Intervention - MICCAI 2025, Pt V | Conference proceeding, workshop paper, or other non-journal publication |
| CheXalign: Preference fine-tuning in chest X-ray interpretation models without human feedback | 2025; Proceedings of the 63rd Annual Meeting of the Association for Computational Linguistics, Vol 1: Long Papers | Conference proceeding, workshop paper, or other non-journal publication |
| Clinical-Grade Multi-organ Pathology Report Generation for Multi-scale Whole Slide Images via a Semantically Guided Medical Text Foundation Model | 2024; Medical Image Computing and Computer Assisted Intervention - MICCAI 2024, Pt Iv | Conference proceeding, workshop paper, or other non-journal publication |
| A Clinically-Informed Framework for Evaluating Vision-Language Models in Radiology Report Generation: Taxonomy of Errors and Risk-Aware Metric. | 2025; medRxiv [Preprint] | Preprint or other non-peer-reviewed full text |
| CoCa-CXR: Contrastive Captioners Learn Strong Temporal Structures for Chest X-Ray Vision-Language Understanding | 2026; Medical Image Computing and Computer Assisted Intervention - MICCAI 2025, Pt Vi | Conference proceeding, workshop paper, or other non-journal publication |
| ColonEye: Towards Colonoscopy Automation Through Wireless Robotic Control and Automated Reporting Using Vision Language Models | 2025; 2025 International Conference on Smart Applications, Communications and Networking, Smartnets | Conference proceeding, workshop paper, or other non-journal publication |
| Comparison on Qwen2.5 and GPT-4o models for generating structured thyroid ultrasound reports | 2025; Chinese Journal of Medical Imaging Technology | Non-English full text |
| Continually Tuning a Large Language Model for Multi-domain Radiology Report Generation | 2024; Medical Image Computing and Computer Assisted Intervention - MICCAI 2024, Pt V | Conference proceeding, workshop paper, or other non-journal publication |
| Contrastive Knowledge-Guided Large Language Models for Medical Report Generation | 2026; Medical Image Computing and Computer Assisted Intervention - MICCAI 2025, Pt Vi | Conference proceeding, workshop paper, or other non-journal publication |
| Contrastive Learning with Counterfactual Explanations for Radiology Report Generation | 2025; Computer Vision-ECCV 2024, Pt Xliii | Conference proceeding, workshop paper, or other non-journal publication |
| Contrastive Learning with Feature Space Interpolation for Retrieval-Based Chest X-Ray Report Generation | 2026; Applied Sciences-Basel | Not an LLM/VLM/foundation-model-based report-generation intervention |
| Contrastive Learning with Feature-Space Interpolation for Retrieval-Based Radiology Report Generation | 2026; Applied Sciences | Duplicate or overlapping version of another included/excluded study |
| CXPMRG-Bench: Pre-training and Benchmarking for X-ray Medical Report Generation on CheXpert Plus Dataset | 2025; 2025 IEEE/Cvf Conference on Computer Vision and Pattern Recognition, CVPR | Conference proceeding, workshop paper, or other non-journal publication |
| CXR-IRGen: An Integrated Vision and Language Model for the Generation of Clinically Accurate Chest X-Ray Image-Report Pairs | 2024; 2024 IEEE/Cvf Winter Conference on Applications of Computer Vision, Wacv 2024 | Conference proceeding, workshop paper, or other non-journal publication |
| A DeepSeek-Powered AI System for Automated Chest Radiograph Interpretation in Clinical Practice | 2025; arXiv preprint | Preprint or other non-peer-reviewed full text |
| Dia-LLaMA: Towards Large Language Model-Driven CT Report Generation | 2026; Medical Image Computing and Computer Assisted Intervention - MICCAI 2025, Pt Vii | Conference proceeding, workshop paper, or other non-journal publication |
| Diff-RRG: Longitudinal Disease-Wise Patch Difference as Guidance for LLM-Based Radiology Report Generation | 2026; Medical Image Computing and Computer Assisted Intervention - MICCAI 2025, Pt Vii | Conference proceeding, workshop paper, or other non-journal publication |
| Direct Preference Optimization for Suppressing Hallucinated Prior Exams in Radiology Report Generation | 2024; Machine Learning for Healthcare Conference | Conference proceeding, workshop paper, or other non-journal publication |
| Diversity-Augmented Diffusion Network with LLM Assistance for Radiology Report Generation | 2025; Companion Proceedings of the ACM Web Conference 2025, Www Companion 2025 | Conference proceeding, workshop paper, or other non-journal publication |
| Drafting the Future: The Dawn of AI Report Generation in Radiology | 2025; Radiology | Review, commentary, or no eligible original data |
| Empowering PET Imaging Reporting with Retrieval-Augmented Large Language Models and Reading Reports Database: A Pilot Single Center Study | 2024; medRxiv preprint | Preprint or other non-peer-reviewed full text |
| Enhancing clinical reasoning in quantized vision–language models for chest X-ray report generation through chain-of-thought teacher–student distillation | 2025; medRxiv preprint | Preprint or other non-peer-reviewed full text |
| Enhancing ECG Report Generation With Domain-Specific Tokenization for Improved Medical NLP Accuracy | 2025; IEEE Access | Wrong task or domain for the review question |
| Evaluating Consistency and Accuracy of GPT-4 Omni to Analyze Thyroid Ultrasound Features and ACR TR Categories to Aid Report Generation. | 2026; Current medical imaging | No eligible medical report, report draft, or diagnostic impression generation output |
| Evaluating Generative Artificial Intelligence as an Educational Tool for Radiology Resident Report Drafting. | 2025; J Am Coll Radiol | Wrong task or domain for the review question |
| Evaluating the Feasibility of Using Smaller Large Language Models for Generating Impressions from Findings in Radiology Reports | 2026; Artificial Intelligence in Healthcare, Aiih 2025, Pt I | Conference proceeding, workshop paper, or other non-journal publication |
| Evaluation of ChatGPT's Capabilities in Medical Report Generation. | 2023; Cureus | No eligible original dataset or denominator-transparent evaluation for the review question |
| Evaluation of GPT-4's Chest X-Ray Impression Generation: A Reader Study on Performance and Perception | 2023; arXiv preprint | Unpublished or non-journal manuscript with no eligible journal publication identified |
| Exploring the Design Space of 3D MLLMs for CT Report Generation | 2026; Medical Image Computing and Computer Assisted Intervention - MICCAI 2025, Pt Vi | Conference proceeding, workshop paper, or other non-journal publication |
| EyeGraphGPT: Knowledge Graph Enhanced Multimodal Large Language Model for Ophthalmic Report Generation | 2024; 2024 IEEE International Conference on Bioinformatics and Biomedicine, BIBM | Conference proceeding, workshop paper, or other non-journal publication |
| Fact-Aware Multimodal Retrieval Augmentation for Accurate Medical Radiology Report Generation | 2025; Proceedings of the 2025 Conference of the Nations of the Americas Chapter of the Association for Computational Linguistics: Human Language Technologies, Vol 1: Long Papers | Conference proceeding, workshop paper, or other non-journal publication |
| Fact-Checking of AI-Generated Reports | 2024; Machine Learning in Medical Imaging, Mlmi 2023, Pt Ii | Conference proceeding, workshop paper, or other non-journal publication |
| FactCheXcker: Mitigating Measurement Hallucinations in Chest X-ray Report Generation Models | 2025; 2025 IEEE/Cvf Conference on Computer Vision and Pattern Recognition (CVPR) | Conference proceeding, workshop paper, or other non-journal publication |
| Fine-Grained Image-Text Alignment in Medical Imaging Enables Explainable Cyclic Image-Report Generation | 2024; Proceedings of the 62nd Annual Meeting of the Association for Computational Linguistics, Vol 1: Long Papers | Conference proceeding, workshop paper, or other non-journal publication |
| Foundation Model for Endoscopy Video Analysis via Large-Scale Self-supervised Pre-train | 2023; Medical Image Computing and Computer Assisted Intervention, MICCAI 2023, Pt Ix | Conference proceeding, workshop paper, or other non-journal publication |
| Generating Clinically Relevant Reports from Chest X-Rays for Cardiomegaly Diagnosis | 2025; 2025 International Conference on Digital Image Computing: Techniques and Applications, Dicta | Conference proceeding, workshop paper, or other non-journal publication |
| Generative AI and foundation models in medical image | 2025; Radiological Physics and Technology | Review, commentary, or no eligible original data |
| Glaucoma Detection and Structured OCT Report Generation via a Fine-tuned Multimodal Large Language Model. | 2025; ArXiv [Preprint] | Preprint or other non-peer-reviewed full text |
| Glaucoma Detection Using Deep Learning and Prompt-Based Explainable Report Generation | 2025; medRxiv preprint | Unpublished or non-journal manuscript with no eligible journal publication identified |
| Harnessing the Power of Pre-trained Vision-Language Models for Efficient Medical Report Generation | 2023; Proceedings of the 32nd ACM International Conference on Information and Knowledge Management, Cikm 2023 | Conference proceeding, workshop paper, or other non-journal publication |
| HC-LLM: Historical-Constrained Large Language Models for Radiology Report Generation | 2025; Thirty-Ninth AAAI Conference on Artificial Intelligence, AAAI-25, Vol 39 No 6 | Conference proceeding, workshop paper, or other non-journal publication |
| HISTOPATHOLOGICAL IMAGE ANALYSIS, DEEP LEARNING AND THE INTEGRATION OF LARGE LANGUAGE MODELS FOR BIOMEDICAL KNOWLEDGE REASONING, DIAGNOSTIC REPORTING AND EXPLAINABILITY | 2025; Biochemistry and Cellular Archives | Review, commentary, or no eligible original data |
| Improving Radiology Report Generation Systems by Removing Hallucinated References to Non-existent Priors | 2022; Machine Learning for Health, Vol 193 | Conference proceeding, workshop paper, or other non-journal publication |
| KARGEN: Knowledge-Enhanced Automated Radiology Report Generation Using Large Language Models | 2024; Medical Image Computing and Computer Assisted Intervention - MICCAI 2024, Pt V | Conference proceeding, workshop paper, or other non-journal publication |
| KHU_LDI at BioLaySumm2025: Fine-tuning and Refinement for Lay Radiology Report Generation | 2025; Proceedings of the 24th Workshop on Biomedical Language Processing, Shared Tasks | Conference proceeding, workshop paper, or other non-journal publication |
| Knowledge Distillation and Transformer-Based Framework for Automatic Spine CT Report Generation | 2025; IEEE Access | Not an LLM/VLM/foundation-model-based report-generation intervention |
| Large language model-enabled automation of diagnostic impressions generation in breast MRI reports | 2025; Chinese Journal of Clinical Oncology | Non-English full text |
| Large language models for structured reporting in radiology: performance of GPT-4, ChatGPT-3.5, Perplexity and Bing. | 2023; La Radiologia medica | No eligible medical report, report draft, or diagnostic impression generation output |
| Leveraging Prompt Engineering with Lightweight Large Language Models to Label and Extract Clinical Information from Radiology Reports | 2025; Companion Proceedings of the ACM Web Conference 2025, Www Companion 2025 | Conference proceeding, workshop paper, or other non-journal publication |
| LLM-Driven Chest X-Ray Report Generation With a Modular, Reduced-Size Architecture | 2025; Intelligent Systems, Bracis 2024, Pt Ii | Conference proceeding, workshop paper, or other non-journal publication |
| LLM-RG4: Flexible and Factual Radiology Report Generation Across Diverse Input Contexts | 2025; Thirty-Ninth AAAI Conference on Artificial Intelligence, AAAI-25, Vol 39 No 8 | Conference proceeding, workshop paper, or other non-journal publication |
| Longitudinal data and a semantic similarity reward for chest X-ray report generation | 2024; arXiv preprint | Preprint or other non-peer-reviewed full text |
| LoRA-MedSim: An Enhanced Multimodal Framework for Clinical Reasoning and Realistic Patient-Doctor Interaction | 2025; Health Psychology Research | Wrong task or domain for the review question |
| MAIRA-Seg: Enhancing Radiology Report Generation with Segmentation-Aware Multimodal Large Language Models | 2024; Machine Learning for Health, ML4H | Conference proceeding, workshop paper, or other non-journal publication |
| MCA-RG: Enhancing LLMs with Medical Concept Alignment for Radiology Report Generation | 2026; Medical Image Computing and Computer Assisted Intervention - MICCAI 2025, Pt V | Conference proceeding, workshop paper, or other non-journal publication |
| Medical Multimodal Model Stealing Attacks via Adversarial Domain Alignment | 2025; Thirty-Ninth AAAI Conference on Artificial Intelligence, AAAI-25, Vol 39 No 7 | Conference proceeding, workshop paper, or other non-journal publication |
| MEPNet: Medical Entity-Balanced Prompting Network for Brain CT Report Generation | 2025; Thirty-Ninth AAAI Conference on Artificial Intelligence, AAAI-25, Vol 39 No 24 | Conference proceeding, workshop paper, or other non-journal publication |
| MIRA: A Novel Framework for Fusing Modalities in Medical RAG | 2025; Proceedings of the 33rd ACM International Conference on Multimedia, Mm 2025 | Conference proceeding, workshop paper, or other non-journal publication |
| MRGAgents: A Multi-Agent Framework for Improved Medical Report Generation with Med-LVLMs | 2025; 2025 International Conference on Digital Image Computing: Techniques and Applications, Dicta | Conference proceeding, workshop paper, or other non-journal publication |
| A Multimodal Vision-text AI Copilot for Brain Disease Diagnosis and Medical Imaging | 2025; medRxiv preprint | Preprint or other non-peer-reviewed full text |
| A Novel Dataset for Polyp Segmentation and Detection Using a Vision-Language Model | 2025; IEEE Access | Wrong task or domain for the review question |
| OphthUS-GPT: Multimodal AI for Automated Reporting in Ophthalmic B-Scan Ultrasound | 2025; medRxiv preprint | Preprint or other non-peer-reviewed full text |
| Optimizing Large Language Models in Radiology and Mitigating Pitfalls: Prompt Engineering and Fine-tuning | 2025; Radiographics | Review, commentary, or no eligible original data |
| PathAlign: A vision-language model for whole slide images in histopathology | 2024; Proceedings of the MICCAI Workshop on Computational Pathology | Conference proceeding, workshop paper, or other non-journal publication |
| PatHME: Hierarchical Multi-Expert Knowledge Distillation for Whole Slide Image Analysis | 2025; IEEE Access | Wrong task or domain for the review question |
| Pathology Report Generation and Multimodal Representation Learning for Cutaneous Melanocytic Lesions | 2026; Medical Image Computing and Computer Assisted Intervention - MICCAI 2025, Pt Vi | Conference proceeding, workshop paper, or other non-journal publication |
| Performance of a commercially available Generative Pre-trained Transformer (GPT) in describing radiolucent lesions in panoramic radiographs and establishing differential diagnoses | 2024; Clinical Oral Investigations | Wrong task or domain for the review question |
| Preprocessing Pathology Reports for Vision-Language Model Development | 2024; Proceedings of the MICCAI Workshop on Computational Pathology | Conference proceeding, workshop paper, or other non-journal publication |
| Prompt-Guided Generation of Structured Chest X-Ray Report Using a Pre-trained LLM | 2024; 2024 IEEE International Conference on Multimedia and Expo, Icme 2024 | Conference proceeding, workshop paper, or other non-journal publication |
| RadAlign: Advancing Radiology Report Generation with Vision-Language Concept Alignment | 2026; Medical Image Computing and Computer Assisted Intervention - MICCAI 2025, Pt Vii | Conference proceeding, workshop paper, or other non-journal publication |
| RADAR: Enhancing Radiology Report Generation with Supplementary Knowledge Injection | 2025; Proceedings of the 63rd Annual Meeting of the Association for Computational Linguistics, Vol 1: Long Papers | Conference proceeding, workshop paper, or other non-journal publication |
| Radiological Report Generation from Chest X-ray Images Using Pre-trained Word Embeddings | 2023; Wireless Personal Communications | Not an LLM/VLM/foundation-model-based report-generation intervention |
| Random Direct Preference Optimization for Radiography Report Generation | 2026; Foundation Models for General Medical AI, Medagi 2025 | Conference proceeding, workshop paper, or other non-journal publication |
| Report Generation from X-Ray imaging by Retrieval-Augmented Generation and improved Image-Text Matching | 2024; 2024 International Joint Conference on Neural Networks, Ijcnn 2024 | Conference proceeding, workshop paper, or other non-journal publication |
| Retrieval Augmented Chest X-Ray Report Generation using OpenAI GPT models | 2023; Machine Learning for Healthcare Conference | Conference proceeding, workshop paper, or other non-journal publication |
| RRG-DPO: Direct Preference Optimization for Clinically Accurate Radiology Report Generation | 2026; Medical Image Computing and Computer Assisted Intervention - MICCAI 2025, Pt V | Conference proceeding, workshop paper, or other non-journal publication |
| RRGMambaFormer: A hybrid Transformer-Mamba architecture for radiology report generation | 2025; Expert Systems with Applications | Not an LLM/VLM/foundation-model-based report-generation intervention |
| Self-Supervised Anatomical Consistency Learning for Vision-Grounded Medical Report Generation | 2025; Proceedings of the 33rd ACM International Conference on Multimedia, Mm 2025 | Conference proceeding, workshop paper, or other non-journal publication |
| A Semantic Evaluation Framework for Medical Report Generation Using Large Language Models | 2025; Cmc-Computers Materials & Continua | Wrong task or domain for the review question |
| Semantic fine-tuning and cross-modal retrieval-augmented Chinese medical report generation | 2025; Journal of Image and Graphics | Non-English full text |
| SERPENT-VLM : Self-Refining Radiology Report Generation Using Vision Language Models | 2024; Proceedings of the 6th Clinical Natural Language Processing Workshop | Conference proceeding, workshop paper, or other non-journal publication |
| SPEC-CXR: Advancing Clinical Safety Through Entity-Level Performance Evaluation of Chest X-ray Report Generation | 2026; Medical Image Computing and Computer Assisted Intervention - MICCAI 2025, Pt Vii | Conference proceeding, workshop paper, or other non-journal publication |
| spEMO: Leveraging Multi-Modal Foundation Models for Analyzing Spatial Multi-Omic and Histopathology Data | 2025; bioRxiv preprint | Wrong task or domain for the review question |
| Stress-Testing of Multimodal Models in Medical Image-Based Report Generation | 2025; Thirty-Ninth AAAI Conference on Artificial Intelligence, AAAI-25, Vol 39 No 28 | Conference proceeding, workshop paper, or other non-journal publication |
| Style-Aware Radiology Report Generation with RadGraph and Few-Shot Prompting | 2023; Findings of the Association for Computational Linguistics (EMNLP 2023) | Conference proceeding, workshop paper, or other non-journal publication |
| TiBiX: Leveraging Temporal Information for Bidirectional X-Ray and Report Generation | 2025; Deep Generative Models, DGM4MICCAI 2024 | Conference proceeding, workshop paper, or other non-journal publication |
| Transform(AI)ng Radiology with CheXSBT: Integrating Dual-Attention Swin Transformer with BERT for Seamless Chest X-Ray Report Generation | 2026; Medical Image Understanding and Analysis, Miua 2025, Pt I | Conference proceeding, workshop paper, or other non-journal publication |
| TriDx: a unified GAN-CNN-GenAI framework for accurate and accessible spinal metastases diagnosis | 2025; Engineering Research Express | Wrong task or domain for the review question |
| TriMedLM: Advancing Three-Dimensional Medical Image Analysis with Multi-Modal LLM | 2024; 2024 IEEE International Conference on Bioinformatics and Biomedicine, BIBM | Conference proceeding, workshop paper, or other non-journal publication |
| TRRG: Towards Truthful Radiology Report Generation With Cross-Modal Disease Clue Enhanced Large Language Models | 2026; Medical Image Computing and Computer Assisted Intervention - MICCAI 2025, Pt Vii | Conference proceeding, workshop paper, or other non-journal publication |
| Uncertainty Estimation in Large Vision Language Models for Automated Radiology Report Generation | 2024; Machine Learning for Health, ML4H | Conference proceeding, workshop paper, or other non-journal publication |
| UniCrossAdapter: Multimodal Adaptation of CLIP for Radiology Report Generation | 2025; Foundation Models for General Medical AI, Medagi 2024 | Conference proceeding, workshop paper, or other non-journal publication |
| A UNIFIED PLATFORM FOR RADIOLOGY REPORT GENERATION AND CLINICIAN-CENTERED AI EVALUATION | 2025; medRxiv preprint | Preprint or other non-peer-reviewed full text |
| Unmasking and quantifying racial bias of large language models in medical report generation | 2024; Communications Medicine | Wrong task or domain for the review question |
| A vision-language model with multi-granular knowledge fusion in medical imaging | 2024; World Wide Web-Internet and Web Information Systems | Preprint or other non-peer-reviewed full text |
| Visual-Textual Cross-Modal Interaction Network for Radiology Report Generation | 2024; IEEE Signal Processing Letters | Not an LLM/VLM/foundation-model-based report-generation intervention |
| X-Gen: Enhancing Radiology Report Generation via LLM-Driven Data Augmentation and Decoupled Training | 2025; 2025 International Conference on Digital Image Computing: Techniques and Applications, Dicta | Conference proceeding, workshop paper, or other non-journal publication |
| Zeus: zero-shot LLM instruction for union segmentation in multimodal medical imaging | 2025; International Journal of Machine Learning and Cybernetics | Wrong task or domain for the review question |
| μ2 Tokenizer: Differentiable Multi-Scale Multi-Modal Tokenizer for Radiology Report Generation | 2026; Medical Image Computing and Computer Assisted Intervention - MICCAI 2025, Pt V | Conference proceeding, workshop paper, or other non-journal publication |

**Supplementary Table S3A. Risk of Bias Framework Domains**

| **Framework component** | **Operational interpretation in this review** |
| --- | --- |
| ROBINS-I logic | Confounding; selection; exposure; missing data; outcome measurement; selective reporting. |
| Reporting appraisal informed by CLAIM | AI reporting: model/prompt; validation setting; failure handling; outcome reporting. |
| Custom AI validation domain: case selection | Case mix; clinical setting; benchmark-only risk. |
| Custom AI validation domain: reference standard | Expert reference quality; clinical grounding; eligible output target. |
| Custom AI validation domain: blinding | Blinding for quality, safety, preference, and acceptance ratings. |
| Custom AI validation domain: validation independence/leakage | Training/tuning/validation/test separation; repeated benchmark risk. |
| Custom AI validation domain: model/prompt transparency | Model identity; prompt strategy; workflow reproducibility. |
| Custom AI validation domain: failed or missing generations | Failed, empty, unusable, or excluded outputs counted. |
| Custom AI validation domain: outcome definition/selective reporting | Error definitions; denominators; workflow outcomes; prespecification. |

**Supplementary Table S3B. Overall Risk of Bias Judgments at the Study Level**

| **Study citation** | **Article title** | **Overall risk-of-bias judgment and framework** | **Main high/serious domains** |
| --- | --- | --- | --- |
| Zhang et al, 2026 [9] | A Fine-tuning Multimodal Large Language Model for Endoscopic Report Generation | High risk; Custom AI validation RoB domains + CLAIM adjunct | Case selection: High; Blinding: High; Validation/leakage: High; Model/prompt transparency: High; Failure handling: High |
| Joyson et al, 2026 [10] | A multi-modal fusion-based deep learning with finetuned LLaMA 3 for lung disease diagnosis using PACS radiology reports | High risk; Custom AI validation RoB domains + CLAIM adjunct | Case selection: High; Blinding: High; Validation/leakage: High; Model/prompt transparency: High; Failure handling: High |
| Liu et al, 2024 [16] | A systematic evaluation of GPT-4V's multimodal capability for chest X-ray image analysis | Serious risk; ROBINS-I + CLAIM adjunct | Confounding: Serious; Participant selection: Serious; Outcome measurement: Serious |
| Bosbach et al, 2024 [119] | Ability of ChatGPT to generate competent radiology reports for distal radius fracture by use of RSNA template items and integrated AO classifier | High risk; Custom AI validation RoB domains + CLAIM adjunct | Case selection: High; Reference standard: High; Blinding: High; Validation/leakage: High; Failure handling: High; Outcome definition/reporting: High |
| Wang et al, 2026 [46] | Activating Associative Disease-Aware Vision Token Memory for LLM-Based X-Ray Report Generation. | Moderate risk; Custom AI validation RoB domains + CLAIM adjunct | Reference standard: High; Blinding: High; Failure handling: High; Outcome definition/reporting: High |
| Serapio et al, 2024 [58] | An open-source fine-tuned large language model for radiological impression generation: a multi-reader performance study. | Serious risk; ROBINS-I + CLAIM adjunct | Confounding: Serious |
| Yeasin et al, 2024 [5] | Auto-Rad: End-to-End Report Generation from Lumber Spine MRI Using Vision-Language Model. | Moderate risk; Custom AI validation RoB domains + CLAIM adjunct | Reference standard: High; Blinding: High; Failure handling: High; Outcome definition/reporting: High |
| Fang et al, 2025 [120] | Automated generation of chest X-ray imaging diagnostic reports by multimodal and multi granularity features fusion | High risk; Custom AI validation RoB domains + CLAIM adjunct | Case selection: High; Blinding: High; Validation/leakage: High; Model/prompt transparency: High; Failure handling: High |
| Zhong et al, 2026 [4] | ChatRadio-Valuer: A Chat Large Language Model for Generalizable Radiology Impression Generation on Multi-Institution and Multi-System Data. | Moderate risk; Custom AI validation RoB domains + CLAIM adjunct | Reference standard: High; Blinding: High; Model/prompt transparency: High; Failure handling: High; Outcome definition/reporting: High |
| Edirisinghe et al, 2025 [121] | Chest X-Ray Report Generation Using Abnormality Guided Vision Language Model | High risk; Custom AI validation RoB domains + CLAIM adjunct | Case selection: High; Blinding: High; Validation/leakage: High; Model/prompt transparency: High; Failure handling: High |
| Tanno et al, 2025 [52] | Collaboration between clinicians and vision-language models in radiology report generation. | Moderate risk; Custom AI validation RoB domains + CLAIM adjunct | Validation/leakage: High; Failure handling: High; Outcome definition/reporting: High |
| Lee et al, 2024 [6] | Comparative Analysis of M4CXR, an LLM-Based Chest X-Ray Report Generation Model, and ChatGPT in Radiological Interpretation. | Serious risk; ROBINS-I + CLAIM adjunct | Confounding: Serious; Participant selection: Serious; Outcome measurement: Serious |
| Li et al, 2025 [122] | Context-enhanced framework for medical image report generation using multimodal contexts | High risk; Custom AI validation RoB domains + CLAIM adjunct | Case selection: High; Reference standard: High; Blinding: High; Validation/leakage: High; Model/prompt transparency: High; Failure handling: High; Outcome definition/reporting: High |
| Lin et al, 2026 [123] | DC-RRG: Diagnosis-centered cascaded radiology report generation | High risk; Custom AI validation RoB domains + CLAIM adjunct | Case selection: High; Reference standard: High; Blinding: High; Validation/leakage: High; Model/prompt transparency: High; Failure handling: High; Outcome definition/reporting: High |
| Hong et al, 2025 [51] | Diagnostic Accuracy and Clinical Value of a Domain-specific Multimodal Generative AI Model for Chest Radiograph Report Generation. | Moderate risk; Custom AI validation RoB domains + CLAIM adjunct | Validation/leakage: High; Model/prompt transparency: High; Failure handling: High; Outcome definition/reporting: High |
| Han et al, 2026 [48] | Dynamic feature fusion guiding and multimodal large language model refining for medical image report generation | High risk; Custom AI validation RoB domains + CLAIM adjunct | Case selection: High; Blinding: High; Validation/leakage: High; Model/prompt transparency: High; Failure handling: High |
| Izhar et al, 2025 [124] | Engaging Preference Optimization Alignment in Large Language Model for Continual Radiology Report Generation: A Hybrid Approach | High risk; Custom AI validation RoB domains + CLAIM adjunct | Case selection: High; Blinding: High; Validation/leakage: High; Model/prompt transparency: High; Failure handling: High |
| Leonardi et al, 2024 [125] | Enhancing radiology report generation through pre-trained language models | High risk; Custom AI validation RoB domains + CLAIM adjunct | Case selection: High; Blinding: High; Validation/leakage: High; Model/prompt transparency: High; Failure handling: High |
| Kim et al, 2025 [17] | Enhancing Structured Pathology Report Generation With Foundation Model and Modular Design | Moderate risk; Custom AI validation RoB domains + CLAIM adjunct | Blinding: High; Model/prompt transparency: High; Failure handling: High |
| Wang et al, 2026 [57] | Evaluation of large language models for diagnostic impression generation from brain MRI report findings: a multicenter benchmark and reader study. | Serious risk; ROBINS-I + CLAIM adjunct | Confounding: Serious |
| Aslam et al, 2025 [126] | Generative Artificial Intelligence for the Automated Generation of Medical Reports: A Modified Poor and Rich Optimization (MPRO)-Based Approach | High risk; Custom AI validation RoB domains + CLAIM adjunct | Case selection: High; Blinding: High; Validation/leakage: High; Model/prompt transparency: High; Failure handling: High |
| Voinea et al, 2024 [56] | GPT-Driven Radiology Report Generation with Fine-Tuned Llama 3. | High risk; Custom AI validation RoB domains + CLAIM adjunct | Case selection: High; Reference standard: High; Blinding: High; Validation/leakage: High; Failure handling: High; Outcome definition/reporting: High |
| Deng et al, 2026 [127] | Grounded knowledge-enhanced medical vision-language pre-training for chest X-ray | High risk; Custom AI validation RoB domains + CLAIM adjunct | Case selection: High; Reference standard: High; Blinding: High; Validation/leakage: High; Failure handling: High; Outcome definition/reporting: High |
| Noh et al, 2026 [47] | Hybrid framework for lesion-aware, clinically coherent chest X-ray report generation using contrastive learning and large language models. | High risk; Custom AI validation RoB domains + CLAIM adjunct | Case selection: High; Blinding: High; Validation/leakage: High; Model/prompt transparency: High; Failure handling: High |
| Chen et al, 2025 [7] | Large Language Model With Region-Guided Referring and Grounding for CT Report Generation. | Moderate risk; Custom AI validation RoB domains + CLAIM adjunct | Reference standard: High; Blinding: High; Model/prompt transparency: High; Failure handling: High; Outcome definition/reporting: High |
| Massimi et al, 2026 [8] | Large Language Model-Driven Analysis and Report Generation of Endoscopy Videos-A Pilot Study. | High risk; Custom AI validation RoB domains + CLAIM adjunct | Case selection: High; Reference standard: High; Blinding: High; Validation/leakage: High; Model/prompt transparency: High; Failure handling: High; Outcome definition/reporting: High |
| Ayaz et al, 2025 [55] | MedVLM: Medical Vision-Language Model for Consumer Devices | High risk; Custom AI validation RoB domains + CLAIM adjunct | Case selection: High; Blinding: High; Validation/leakage: High; Failure handling: High |
| Liu et al, 2025 [11] | MetaGP: A Generative Foundation Model Integrating Electronic Health Records and Multimodal Imaging for Addressing Unmet Clinical Needs | High risk; Custom AI validation RoB domains + CLAIM adjunct | Case selection: High; Reference standard: High; Blinding: High; Validation/leakage: High; Model/prompt transparency: High; Failure handling: High; Outcome definition/reporting: High |
| Dasanayaka et al, 2025 [12] | Multimodal AI and Large Language Models for Orthopantomography Radiology Report Generation and Q&A | High risk; Custom AI validation RoB domains + CLAIM adjunct | Case selection: High; Reference standard: High; Blinding: High; Validation/leakage: High; Model/prompt transparency: High; Failure handling: High; Outcome definition/reporting: High |
| Yan et al, 2025 [18] | Multimodal Medical Image Analysis: Integrating LLM and RAG Deep Learning Strategies | High risk; Custom AI validation RoB domains + CLAIM adjunct | Case selection: High; Reference standard: High; Blinding: High; Validation/leakage: High; Failure handling: High; Outcome definition/reporting: High |
| Zhang et al, 2024 [118] | Potential of multimodal large language models for data mining of medical images and free-text reports | High risk; Custom AI validation RoB domains + CLAIM adjunct | Case selection: High; Reference standard: High; Blinding: High; Validation/leakage: High; Model/prompt transparency: High; Failure handling: High; Outcome definition/reporting: High |
| Soleimani et al, 2024 [60] | Practical Evaluation of ChatGPT Performance for Radiology Report Generation. | Serious risk; ROBINS-I + CLAIM adjunct | Confounding: Serious; Participant selection: Serious |
| Park et al, 2026 [59] | Prospective evaluation of artificial intelligence (AI) in lumbar spine magnetic resonance imaging (MRI) workflow: from deep learning (DL)-enhanced accelerated acquisition to simultaneous vision-language model (VLM)-based automated report generation. | Serious risk; ROBINS-I + CLAIM adjunct | Confounding: Serious; Participant selection: Serious |
| Wang et al, 2026 [50] | R2GenCSR: Mining Contextual and Residual Information for LLMs-based Radiology Report Generation. | High risk; Custom AI validation RoB domains + CLAIM adjunct | Case selection: High; Reference standard: High; Blinding: High; Validation/leakage: High; Failure handling: High; Outcome definition/reporting: High |
| Wang et al, 2023 [15] | R2GenGPT: Radiology Report Generation with frozen LLMs | High risk; Custom AI validation RoB domains + CLAIM adjunct | Case selection: High; Reference standard: High; Blinding: High; Validation/leakage: High; Failure handling: High; Outcome definition/reporting: High |
| Li et al, 2025 [128] | S-RRG-Bench: Structured radiology report generation with fine-grained evaluation framework | Serious risk; ROBINS-I + CLAIM adjunct | Confounding: Serious; Participant selection: Serious; Outcome measurement: Serious |
| Jia et al, 2026 [49] | Semantic Feedback-Based RAG for Radiology Report Generation | High risk; Custom AI validation RoB domains + CLAIM adjunct | Case selection: High; Blinding: High; Validation/leakage: High; Model/prompt transparency: High; Failure handling: High |
| Azhar et al, 2026 [61] | Semiautomated breast ultrasound report generation using multimodal large language models and deep learning. | High risk; Custom AI validation RoB domains + CLAIM adjunct | Case selection: High; Reference standard: High; Blinding: High; Validation/leakage: High; Model/prompt transparency: High; Failure handling: High; Outcome definition/reporting: High |
| Helmy et al, 2026 [13] | SPINE: Segmentation-guided Processing and Integration of Multimodal Spinal MRI for Natural-Language Enhanced Report Generation | High risk; Custom AI validation RoB domains + CLAIM adjunct | Case selection: High; Reference standard: High; Blinding: High; Validation/leakage: High; Model/prompt transparency: High; Failure handling: High; Outcome definition/reporting: High |
| Chen et al, 2025 [62] | The Potential of Gemini and GPTs for Structured Report Generation based on Free-Text (18)F-FDG PET/CT Breast Cancer Reports. | High risk; Custom AI validation RoB domains + CLAIM adjunct | Case selection: High; Reference standard: High; Blinding: High; Validation/leakage: High; Failure handling: High; Outcome definition/reporting: High |
| Li et al, 2025 [53] | Towards a holistic framework for multimodal LLM in 3D brain CT radiology report generation. | Moderate risk; Custom AI validation RoB domains + CLAIM adjunct | Failure handling: High |
| Luo et al, 2026 [54] | Towards Automated Reporting: A Bronchoscopy Report Dataset for Enhancing Multimodality Large Language Models. | Moderate risk; Custom AI validation RoB domains + CLAIM adjunct | Case selection: High; Validation/leakage: High; Model/prompt transparency: High; Failure handling: High; Outcome definition/reporting: High |
| Gholipour et al, 2026 [39] | Trust but verify: Image-aware evaluation of radiology report generators | Serious risk; ROBINS-I + CLAIM adjunct | Confounding: Serious; Participant selection: Serious; Outcome measurement: Serious |
| Ucan et al, 2025 [129] | Turkish Chest X-Ray Report Generation Model Using the Swin Enhanced Yield Transformer (Model-SEY) Framework | High risk; Custom AI validation RoB domains + CLAIM adjunct | Case selection: High; Blinding: High; Validation/leakage: High; Model/prompt transparency: High; Failure handling: High |
| Li et al, 2025 [14] | Ultrasound report generation with fuzzy knowledge and multi-modal large language model | High risk; Custom AI validation RoB domains + CLAIM adjunct | Case selection: High; Reference standard: High; Blinding: High; Validation/leakage: High; Model/prompt transparency: High; Failure handling: High; Outcome definition/reporting: High |
| Islam et al, 2025 [130] | Vision-Language Models for Automated Chest X-ray Interpretation: Leveraging ViT and GPT-2 | High risk; Custom AI validation RoB domains + CLAIM adjunct | Case selection: High; Blinding: High; Validation/leakage: High; Failure handling: High |
| Bosbach et al, 2026 [63] | Conceptual proposal for LLM-generated FDG PET/CT follow-up reports in melanoma: a pilot study on model stability and blinded expert evaluation. | Serious risk; ROBINS-I + CLAIM adjunct | Confounding: Serious; selection of participants or case mix: Serious; Outcome measurement: Serious |
| Lorusso et al, 2026 [64] | Comparative evaluation of large language models for generating CAD-RADS 2.0-compliant diagnostic conclusions in cardiac CT reports. | High risk; ROBINS-I + CLAIM adjunct | Confounding: High |
| Zanardo et al, 2026 [65] | Can AI write reports like a radiologist? A blinded evaluation of large language model-generated lumbar spine MRI reports. | High risk; ROBINS-I + CLAIM adjunct | Confounding: High |
| Tran et al, 2025 [66] | Generating dermatopathology reports from gigapixel whole slide images with HistoGPT. | Moderate risk; Custom AI validation RoB domains + CLAIM adjunct | failed or missing generation handling: High |
| Raminedi et al, 2024 [67] | Multi-modal transformer architecture for medical image analysis and automated report generation. | High risk; Custom AI validation RoB domains + CLAIM adjunct | Case selection: High; Blinding: High; validation independence and data leakage: High; failed or missing generation handling: High; Outcome definition/reporting: High |
| Liu et al, 2026 [68] | SpineVLM: A Markdown-Guided Structured Fine-Tuning Framework for Spine X-ray Report Generation. | High risk; ROBINS-I + CLAIM adjunct | Confounding: High |
| Ye et al, 2026 [69] | Report Generation System for Slit-Lamp Image Interpretation Using Vision-Language Models | High risk; Custom AI validation RoB domains + CLAIM adjunct | Case selection: High; Blinding: High; validation independence and data leakage: High; failed or missing generation handling: High; Outcome definition/reporting: High |
| Tan et al, 2026 [70] | Large language model-assisted radiology reporting in a single-radiologist implementation: a retrospective cohort study interpreted through a UTAUT lens. | Serious risk; ROBINS-I + CLAIM adjunct | Confounding: Serious; selection of participants or case mix: Serious; Outcome measurement: Serious |
| Li et al, 2026 [71] | Fine-Tuned Large Language Model for Automated Radiology Impression Generation: A Multicenter Evaluation. | Moderate risk; ROBINS-I + CLAIM adjunct | None high/serious |
| Barzegar et al, 2026 [72] | Enhancing chest X-ray report generation with pathology-guided prompts and vision-language shortcut bias mitigation | High risk; Custom AI validation RoB domains + CLAIM adjunct | Case selection: High; Blinding: High; validation independence and data leakage: High; failed or missing generation handling: High; Outcome definition/reporting: High |
| Allaberdiev et al, 2026 [73] | Chestxgen: Dynamic Memory-Augmented Vision-Language Transformer with Context-Aware Gating for Radiology Report Generation | High risk; Custom AI validation RoB domains + CLAIM adjunct | Case selection: High; Blinding: High; validation independence and data leakage: High; failed or missing generation handling: High; Outcome definition/reporting: High |
| Lin et al, 2026 [74] | Automatic Speech Recognition and Large Language Models for Multilingual Pathology Report Generation: Proof-of-Concept Study. | Serious risk; Custom AI validation RoB domains + CLAIM adjunct | Case selection: Serious; Reference standard: High; Blinding: High; validation independence and data leakage: Serious; model or prompt transparency: High; failed or missing generation handling: Serious; Outcome definition/reporting: High |
| Khatoon et al, 2026 [75] | Automated Radiological Report Generation from Breast Ultrasound Images Using Vision and Language Transformers. | High risk; Custom AI validation RoB domains + CLAIM adjunct | Case selection: High; Blinding: High; validation independence and data leakage: High; failed or missing generation handling: High; Outcome definition/reporting: High |
| Park et al, 2026 [76] | A robust vision language model for molecular status prediction and radiology report generation in adult-type diffuse gliomas. | Moderate risk; Custom AI validation RoB domains + CLAIM adjunct | failed or missing generation handling: High |
| Hossain et al, 2025 [77] | Privacy-Preserving Chest X-Ray Report Generation via Multimodal Federated Learning with ViT and GPT2 | High risk; Custom AI validation RoB domains + CLAIM adjunct | Case selection: High; Blinding: High; validation independence and data leakage: High; failed or missing generation handling: High; Outcome definition/reporting: High |
| Hosseini et al, 2025 [78] | M3: multimodal artificial intelligence for medical report generation and visual question answering from 3D abdominal CT scans. | High risk; Custom AI validation RoB domains + CLAIM adjunct | Case selection: High; Blinding: High; validation independence and data leakage: High; failed or missing generation handling: High; Outcome definition/reporting: High |
| Wang et al, 2024 [79] | Enhancing Diagnostic Accuracy and Efficiency with GPT-4-Generated Structured Reports: A Comprehensive Study | High risk; ROBINS-I + CLAIM adjunct | Confounding: High |
| Zhang et al, 2024 [80] | Constructing a Large Language Model to Generate Impressions from Findings in Radiology Reports. | High risk; ROBINS-I + CLAIM adjunct | Confounding: High |
| Sun et al, 2023 [81] | Evaluating GPT4 on Impressions Generation in Radiology Reports. | High risk; ROBINS-I + CLAIM adjunct | Confounding: High |
| Huang et al, 2023 [82] | Generative Artificial Intelligence for Chest Radiograph Interpretation in the Emergency Department. | High risk; ROBINS-I + CLAIM adjunct | Confounding: High |
| Mallio et al, 2024 [83] | ChatGPT in radiology structured reporting: analysis of ChatGPT-3.5 Turbo and GPT-4 in reducing word count and recalling findings. | High risk; Custom AI validation RoB domains + CLAIM adjunct | Case selection: High; Blinding: High; validation independence and data leakage: High; failed or missing generation handling: High; Outcome definition/reporting: High |
| Stephan et al, 2024 [84] | AI in Dental Radiology-Improving the Efficiency of Reporting With ChatGPT: Comparative Study. | High risk; ROBINS-I + CLAIM adjunct | Confounding: High |
| Kim et al, 2025 [85] | Utility of Multimodal Large Language Models in Analyzing Chest X-Rays with Incomplete Contextual Information. | High risk; ROBINS-I + CLAIM adjunct | Confounding: High |
| Di et al, 2025 [86] | Structured Transformation of Unstructured Prostate MRI Reports Using Large Language Models. | High risk; Custom AI validation RoB domains + CLAIM adjunct | Case selection: High; Blinding: High; validation independence and data leakage: High; failed or missing generation handling: High; Outcome definition/reporting: High |
| Xie et al, 2025 [87] | Large language models for efficient whole-organ MRI score-based reports and categorization in knee osteoarthritis. | High risk; ROBINS-I + CLAIM adjunct | Confounding: High |
| Shao et al, 2025 [88] | Generative artificial intelligence for fundus fluorescein angiography interpretation and human expert evaluation | Moderate risk; ROBINS-I + CLAIM adjunct | None high/serious |
| Gupta et al, 2025 [89] | Enhancing Radiological Reporting in Head and Neck Cancer: Converting Free-Text CT Scan Reports to Structured Reports Using Large Language Models. | High risk; Custom AI validation RoB domains + CLAIM adjunct | Case selection: High; Blinding: High; validation independence and data leakage: High; failed or missing generation handling: High; Outcome definition/reporting: High |
| Lee et al, 2025 [90] | CXR-LLaVA: a multimodal large language model for interpreting chest X-ray images. | High risk; Custom AI validation RoB domains + CLAIM adjunct | Case selection: High; Blinding: High; validation independence and data leakage: High; failed or missing generation handling: High; Outcome definition/reporting: High |
| Pesapane et al, 2025 [91] | A preliminary investigation into the potential, pitfalls, and limitations of large language models for mammography interpretation | High risk; Custom AI validation RoB domains + CLAIM adjunct | Case selection: High; Blinding: High; validation independence and data leakage: High; failed or missing generation handling: High; Outcome definition/reporting: High |
| Hartsock et al, 2026 [92] | Improving Radiology Report Conciseness and Structure via Local Large Language Models. | High risk; Custom AI validation RoB domains + CLAIM adjunct | Case selection: High; Blinding: High; validation independence and data leakage: High; failed or missing generation handling: High; Outcome definition/reporting: High |
| Silbergleit et al, 2025 [93] | ChatGPT vs Gemini: Comparative Accuracy and Efficiency in CAD-RADS Score Assignment from Radiology Reports. | High risk; ROBINS-I + CLAIM adjunct | Confounding: High |
| Adams et al, 2023 [94] | Leveraging GPT-4 for Post Hoc Transformation of Free-text Radiology Reports into Structured Reporting: A Multilingual Feasibility Study. | Serious risk; Custom AI validation RoB domains + CLAIM adjunct | Case selection: Serious; Reference standard: High; Blinding: High; validation independence and data leakage: Serious; model or prompt transparency: High; failed or missing generation handling: Serious; Outcome definition/reporting: High |
| Jiang et al, 2024 [95] | Transforming free-text radiology reports into structured reports using ChatGPT: A study on thyroid ultrasonography. | High risk; Custom AI validation RoB domains + CLAIM adjunct | Case selection: High; Blinding: High; validation independence and data leakage: High; failed or missing generation handling: High; Outcome definition/reporting: High |
| Hasani et al, 2024 [96] | Evaluating the performance of Generative Pre-trained Transformer-4 (GPT-4) in standardizing radiology reports. | High risk; ROBINS-I + CLAIM adjunct | Confounding: High |
| Tsaniya et al, 2025 [97] | Medical Report Generation With Knowledge Distillation and Multi-Stage Hierarchical Attention in Vision Transformer Encoder and GPT-2 Decoder | High risk; Custom AI validation RoB domains + CLAIM adjunct | Case selection: High; Blinding: High; validation independence and data leakage: High; failed or missing generation handling: High; Outcome definition/reporting: High |
| Yan et al, 2025 [98] | Automated Ultrasound Diagnosis via CLIP-GPT Synergy: A Multimodal Framework for Image Classification and Report Generation | High risk; Custom AI validation RoB domains + CLAIM adjunct | Case selection: High; Blinding: High; validation independence and data leakage: High; failed or missing generation handling: High; Outcome definition/reporting: High |
| López-Úbeda et al, 2025 [99] | Automated MRI pituitary structured reporting from free-text using a fine-tuned Llama model: a feasibility study | High risk; Custom AI validation RoB domains + CLAIM adjunct | Case selection: High; Blinding: High; validation independence and data leakage: High; failed or missing generation handling: High; Outcome definition/reporting: High |
| Tian et al, 2025 [100] | A Medical Multimodal Large Language Model for Pediatric Pneumonia. | High risk; Custom AI validation RoB domains + CLAIM adjunct | Case selection: High; Blinding: High; validation independence and data leakage: High; failed or missing generation handling: High; Outcome definition/reporting: High |
| Li et al, 2026 [101] | Towards generalizable pathology reports via a multimodal LLM with the multicenter in-context learning. | Moderate risk; Custom AI validation RoB domains + CLAIM adjunct | failed or missing generation handling: High |
| Hong et al, 2026 [102] | Temperature Setting of a Multimodal Generative Artificial Intelligence Model: Association With Accuracy and Quality of Artificial Intelligence-Generated Chest Radiograph Reports. | High risk; ROBINS-I + CLAIM adjunct | Confounding: High |
| de et al, 2026 [103] | Reporting efficiency in diagnostic imaging: Can plug-and-play general-purpose large language models outperform conventional speech recognition? | High risk; ROBINS-I + CLAIM adjunct | Confounding: High |
| García et al, 2026 [104] | Large language model-assisted structured reporting in Radiology residents: an implementation pilot study in Emergency Radiology. | Serious risk; ROBINS-I + CLAIM adjunct | Confounding: Serious; selection of participants or case mix: Serious; Outcome measurement: Serious |
| Tasneem et al, 2026 [105] | From radiology findings to artificial intelligence-powered impressions: A retrospective study on the comparative performance of recent large language models | High risk; ROBINS-I + CLAIM adjunct | Confounding: High |
| Pellegrini et al, 2026 [106] | Enhancing radiology workflows through collaborative AI-assisted chest X-ray reporting using large vision-language models: a proof-of-concept study. | Serious risk; ROBINS-I + CLAIM adjunct | Confounding: Serious; selection of participants or case mix: Serious; Outcome measurement: Serious |
| Jiang et al, 2026 [107] | Domain specific multimodal large language model for automated endoscopy reporting with multicenter prospective validation. | Moderate risk; ROBINS-I + CLAIM adjunct | None high/serious |
| Sheng et al, 2026 [108] | BI-RADS-compliant structured mammography reporting using locally deployed large language models under privacy constraints. | High risk; Custom AI validation RoB domains + CLAIM adjunct | Case selection: High; Blinding: High; validation independence and data leakage: High; failed or missing generation handling: High; Outcome definition/reporting: High |
| Liu et al, 2026 [109] | Teaching multimodal LLMs to comprehend 12-lead electrocardiographic images | High risk; Custom AI validation RoB domains + CLAIM adjunct | Case selection: High; Blinding: High; validation independence and data leakage: High; failed or missing generation handling: High; Outcome definition/reporting: High |
| Lee et al, 2026 [110] | Read like a radiologist: Efficient vision-language model for 3D medical imaging interpretation. | High risk; Custom AI validation RoB domains + CLAIM adjunct | Case selection: High; Blinding: High; validation independence and data leakage: High; failed or missing generation handling: High; Outcome definition/reporting: High |
| Alkhaldi et al, 2026 [111] | MiniGPT-Med: A Unified Vision-Language Model for Radology Image Understanding | High risk; Custom AI validation RoB domains + CLAIM adjunct | Case selection: High; Blinding: High; validation independence and data leakage: High; failed or missing generation handling: High; Outcome definition/reporting: High |
| Abdaoui et al, 2026 [112] | MedFusionT5: Cross-Modal Attention Boosts Semantic Quality and Reduces Hallucinations in Dental AI. | High risk; Custom AI validation RoB domains + CLAIM adjunct | Case selection: High; Blinding: High; validation independence and data leakage: High; failed or missing generation handling: High; Outcome definition/reporting: High |
| Mao et al, 2026 [113] | CT-Agent: a multimodal-LLM agent for 3D CT radiology question answering | High risk; Custom AI validation RoB domains + CLAIM adjunct | Case selection: High; Blinding: High; validation independence and data leakage: High; failed or missing generation handling: High; Outcome definition/reporting: High |
| Woźnicki et al, 2025 [114] | Automatic structuring of radiology reports with on-premise open-source large language models. | High risk; Custom AI validation RoB domains + CLAIM adjunct | Case selection: High; Blinding: High; validation independence and data leakage: High; failed or missing generation handling: High; Outcome definition/reporting: High |
| Dong et al, 2025 [115] | Keyword-based AI assistance in the generation of radiology reports: A pilot study | High risk; ROBINS-I + CLAIM adjunct | Confounding: High |
| Tekdemir et al, 2026 [116] | Evaluating Large Language Models for Turkish Emergency CT Impression Drafting: Quality, Critical Omissions, and Readability. | High risk; ROBINS-I + CLAIM adjunct | Confounding: High |
| Rouhizadeh et al, 2026 [117] | The detectability paradox: bilingual medical report generation with open-weight models and the limits of human oversight. | High risk; ROBINS-I + CLAIM adjunct | Confounding: High |

**Supplementary Table S4. Outcome-Level Extraction Summary**

| **Study citation** | **Outcome** | **Task / modality / oversight** | **Comparison structure** | **Extracted numeric data** | **Metric and meta-analysis status** | **Outcome definition and pooling note** |
| --- | --- | --- | --- | --- | --- | --- |
| Hong et al, 2025 [51] | Secondary acceptance outcome / Expert acceptance rate (Key secondary) | Image-to-report generation; Chest X-ray; Autonomous | Multiarm comparative evaluation; AI model: Domain-specific AI model; Comparator: Radiologist report | AI n=8580, events=6047; Control n=8580, events=6288 | Original metric: report acceptance rate; planned effect measure: RR; meta-analysis extractable: no | 70.5% vs 73.3%; clustered reader ratings; no adjusted case-level estimate. |
| Hong et al, 2025 [51] | Primary safety outcome / Commission error rate (primary) | Image-to-report generation; Chest X-ray; Autonomous | Multiarm comparative evaluation; AI model: Domain-specific AI model; Comparator: Radiologist report | AI n=8580, events=971; Control n=8580, events=831 | Original metric: false positive findings; planned effect measure: RR; meta-analysis extractable: no | FP 11.3% vs 9.7%; clustered reader ratings; no unique case-level rate. |
| Hong et al, 2025 [51] | Primary safety outcome / Omission error rate (primary) | Image-to-report generation; Chest X-ray; Autonomous | Multiarm comparative evaluation; AI model: Domain-specific AI model; Comparator: Radiologist report | AI n=8580, events=1584; Control n=8580, events=1527 | Original metric: false negative findings; planned effect measure: RR; meta-analysis extractable: no | FN 18.5% vs 17.8%; clustered reader ratings; no unique case-level rate. |
| Serapio et al, 2024 [58] | Secondary workflow outcome / Reporting time (Key secondary) | Findings/report-to-impression generation; CT/CTA/PET-CT; Human-supervised drafting | Two-arm parallel comparison; AI model: Fine-tuned open-source LLM; Comparator: Subspecialist radiologist impression | AI n=40, mean=18.29 (SD 11.50); Control n=20, mean=12.20 (SD 9.13) | Original metric: edit time (seconds); planned effect measure: MD; meta-analysis extractable: yes | 18.29 vs 12.20 s; extractable continuous outcome. |
| Serapio et al, 2024 [58] | Secondary workflow outcome / Edit distance (Key secondary) | Findings/report-to-impression generation; CT/CTA/PET-CT; Human-supervised drafting | Two-arm parallel comparison; AI model: Fine-tuned open-source LLM; Comparator: Subspecialist radiologist impression | AI n=40, mean=12.32 (SD 8.21); Control n=20, mean=5.74 (SD 4.18) | Original metric: edit distance (words changed); planned effect measure: MD; meta-analysis extractable: yes | 12.32 vs 5.74 words; extractable continuous outcome. |
| Serapio et al, 2024 [58] | Exploratory language metric / ROUGE (exploratory) | Findings/report-to-impression generation; CT/CTA/PET-CT; Human-supervised drafting | Two-arm parallel comparison; AI model: Fine-tuned open-source LLM; Comparator: Subspecialist radiologist impression | AI n=40, mean=84 (SD 10.34); Control n=20, mean=89 (SD 8.61) | Original metric: ROUGE-L against edited impression; planned effect measure: MD; meta-analysis extractable: yes | ROUGE-L 84 vs 89; extractable continuous outcome. |
| Wang et al, 2026 [57] | Secondary workflow outcome / Reporting time (Key secondary) | Findings/report-to-impression generation; MRI; Human-supervised drafting | Paired within-case comparison; AI model: Radiologist with DeepSeek-R1 assistance; Comparator: Radiologist without AI | Narrative or not numerically reconstructable | Original metric: reading time (seconds); planned effect measure: SMD; meta-analysis extractable: no | 61 vs 53 s; paired summary only; no mean/SD/covariance. |
| Tanno et al, 2025 [52] | Primary safety outcome / Clinically significant error rate (primary) | Image-to-report generation; Chest X-ray; Human-supervised drafting | Paired within-case comparison; AI model: Flamingo-CXR; Comparator: Clinician report | Narrative or not numerically reconstructable | Original metric: clinically significant errors; planned effect measure: RR; meta-analysis extractable: no | Errors/report: 0.23 vs 0.20; 0.28 vs 0.14; no pooled case-level counts. |
| Tanno et al, 2025 [52] | Secondary acceptance outcome / Blinded expert preference (Key secondary) | Image-to-report generation; Chest X-ray; Human-supervised | Paired within-case comparison; AI model: Flamingo-CXR; Comparator: Clinician report | Narrative or not numerically reconstructable | Original metric: pairwise preference / equivalence by at least half of panel; planned effect measure: RR; meta-analysis extractable: no | Equivalent/preferred: 77.7% and 56.1%; thresholded panel proportion. |
| Giorgio Leonardi et al, 2024 | Exploratory language metric / BLEU (exploratory) | Report drafting or structured report generation; Chest X-ray; Autonomous | Single-arm expert-referenced evaluation; AI model: ViT_GPT2_custom_PE_beam_search; Comparator: Single-arm expert-referenced evaluation | Narrative or not numerically reconstructable | Original metric: BLEU-4 (100); planned effect measure: SMD; meta-analysis extractable: no | BLEU-4 9.5; single-arm benchmark metric. |
| Giorgio Leonardi et al, 2024 | Exploratory language metric / ROUGE (exploratory) | Report drafting or structured report generation; Chest X-ray; Autonomous | Single-arm expert-referenced evaluation; AI model: ViT_GPT2_custom_PE_beam_search; Comparator: Single-arm expert-referenced evaluation | Narrative or not numerically reconstructable | Original metric: FROUGE-L (100); planned effect measure: SMD; meta-analysis extractable: no | FROUGE-L 38; single-arm benchmark metric. |
| Han et al, 2026 [48] | Exploratory language metric / BLEU (exploratory) | Image-to-report generation; Chest X-ray; Autonomous | Single-arm expert-referenced evaluation; AI model: DFFG_MLLMR; Comparator: Single-arm expert-referenced evaluation | Narrative or not numerically reconstructable | Original metric: BLEU-4; planned effect measure: SMD; meta-analysis extractable: no | BLEU-4 0.132; single-arm benchmark metric. |
| Han et al, 2026 [48] | Exploratory language metric / ROUGE (exploratory) | Image-to-report generation; Chest X-ray; Autonomous | Single-arm expert-referenced evaluation; AI model: DFFG_MLLMR; Comparator: Single-arm expert-referenced evaluation | Narrative or not numerically reconstructable | Original metric: ROUGE-L; planned effect measure: SMD; meta-analysis extractable: no | ROUGE-L 0.305; single-arm benchmark metric. |
| Han et al, 2026 [48] | Exploratory language metric / CIDEr (exploratory) | Image-to-report generation; Chest X-ray; Autonomous | Single-arm expert-referenced evaluation; AI model: DFFG_MLLMR; Comparator: Single-arm expert-referenced evaluation | Narrative or not numerically reconstructable | Original metric: CIDEr; planned effect measure: SMD; meta-analysis extractable: no | CIDEr 0.289; single-arm benchmark metric. |
| Jia et al, 2026 [49] | Exploratory language metric / BLEU (exploratory) | Image-to-report generation; Chest X-ray; Autonomous | Single-arm expert-referenced evaluation; AI model: RAGSemRad; Comparator: Single-arm expert-referenced evaluation | Narrative or not numerically reconstructable | Original metric: BLEU-4; planned effect measure: SMD; meta-analysis extractable: no | BLEU-4 0.118; single-arm benchmark metric. |
| Jia et al, 2026 [49] | Exploratory language metric / ROUGE (exploratory) | Image-to-report generation; Chest X-ray; Autonomous | Single-arm expert-referenced evaluation; AI model: RAGSemRad; Comparator: Single-arm expert-referenced evaluation | Narrative or not numerically reconstructable | Original metric: ROUGE-L; planned effect measure: SMD; meta-analysis extractable: no | ROUGE-L 0.303; single-arm benchmark metric. |

**Supplementary Table S5. Targeted Quantitative Results and Pooling Decisions**

| **Study** | **Clinical source and task** | **Reported result** | **Outcome basis** | **Pooling decision** |
| --- | --- | --- | --- | --- |
| Hong et al, 2025 [51] | X-ray / report generation from images; Autonomous | Acceptance 70.5% vs 73.3%; FP 11.3% vs 9.7%; FN 18.5% vs 17.8%. | Safety and expert acceptance | Not pooled: incompatible denominator/effect structure. |
| Tanno et al, 2025 [52] | X-ray / report generation from images; Human-supervised | Equivalent/preferred: 77.7% and 56.1%; clinically significant errors reported. | Expert preference and clinically significant errors | Not pooled: incompatible denominator/effect structure. |
| Wang et al, 2026 [57] | MRI / impression generation from findings; Editing/structuring support | Diagnostic performance; reading time 61 vs 53 s. | Workflow and diagnostic performance | Not pooled: incompatible denominator/effect structure. |
| Serapio et al, 2024 [58] | Mixed CT/US/MRI / impression generation from findings; Human-supervised | Higher edit time and edit distance than radiologist baseline. | Edit burden and workflow | Not pooled: single compatible study. |
| Luo et al, 2026 [54] | Endoscopy / report generation from images; Autonomous | Clinician acceptance assessment; caption accuracy 82.91%. | Expert oriented quality signal | Not pooled: incompatible denominator/effect structure. |
| de Margerie-Mellon et al, 2026 [91] | Mixed modality / Report drafting/structuring; Human-supervised | Generation time; text errors; confabulations; edit distance. | Workflow or time; human or expert evaluation | Not pooled: incompatible denominator/effect structure. |
| Jiang et al, 2026 [107] | Endoscopy / report generation from images; Autonomous | Acceptable report rate; completeness; accuracy; processing time. | Workflow or time; safety or error | Not pooled: incompatible denominator/effect structure. |
| Lorusso et al, 2026 [64] | CT/CTA/PET-CT / Impression/conclusion generation; Autonomous | Reporting time; quality score; diagnostic accuracy; keyword-order robustness. | Workflow or time; human or expert evaluation; safety or error | Not pooled: incompatible denominator/effect structure. |
| Dong et al, 2025 [115] | MRI / Report drafting/structuring; Human-supervised | Reporting time; quality score; diagnostic accuracy; robustness. | Workflow or time; human or expert evaluation; safety or error | Not pooled: incompatible denominator/effect structure. |
| Pellegrini et al, 2026 [106] | Chest x-ray / report generation from images; Human-supervised | Writing time; suggestion acceptance; quality; usability; satisfaction. | Workflow or time; human or expert evaluation; safety or error; preference or usability | Not pooled: incompatible denominator/effect structure. |
| Tan et al, 2026 [70] | Mixed modality / Report drafting/structuring; Human-supervised | Inter-study interval; training burden; satisfaction; UTAUT workflow outcomes. | Workflow or time; human or expert evaluation; preference or usability | Not pooled: incompatible denominator/effect structure. |
| Wang et al, 2024 [79] | Ultrasound / Report drafting/structuring; Editing/structuring support | Diagnostic time; diagnostic accuracy; clinician feedback. | Workflow or time; human or expert evaluation; safety or error | Not pooled: incompatible denominator/effect structure. |
| Xie et al, 2025 [87] | MRI / Report drafting/structuring; Editing/structuring support | WORMS performance; OA severity; surgeon preference; reading time. | Workflow or time; human or expert evaluation; preference or usability | Not pooled: incompatible denominator/effect structure. |

**Supplementary Table S6. Reasons Clinically Relevant Outcomes Could Not Be Pooled**

| **Outcome domain** | **Main reporting patterns** | **Main reason pooling was not performed** | **Manuscript interpretation** |
| --- | --- | --- | --- |
| Clinically significant errors | Clustered ratings; variable discrepancy thresholds; limited case-level denominators. | Units of analysis and error definitions were not compatible across studies; clustering could not be reconstructed | Narrative safety summary only. |
| Omission errors | False-negative/missed findings; clustered or model-specific summaries. | Denominators, case mix, reader structure, and omission definitions differed substantially | Safety signal; no cross-study rate estimate. |
| Commission errors / hallucinations | False-positive or unsupported text; inconsistent definitions. | Outcome labels mapped to different clinical constructs and reporting units | Report omission and commission separately. |
| Expert acceptance or preference | Acceptance rates, equivalence/preference thresholds, indistinguishability ratings, Likert scores, or panel level preferences | Thresholded and clustered human evaluation structures were incompatible | Summarize as human evaluation signals specific to each task |
| Reporting time / diagnostic time | Reader or workflow comparisons from single settings; some report times by study arm, others report implementation or generation time metrics | Comparator workflows, task difficulty, and available dispersion statistics were inconsistent | Use single study quantitative summaries only when reconstructable |
| Edit burden / edit distance | Edit distance, word changes, correction counts, or qualitative editing burden | Metrics were not standardized and rarely shared compatible mean/SD or paired data | Report direction and context, not pooled effect |
| Diagnostic performance | Accuracy, agreement, F1, sensitivity/specificity, or metrics specific to disease targets | Clinical targets and reference standards differed by modality and task | Use for context within modality/task groups only |
| Automated language or semantic metrics | BLEU, ROUGE, CIDEr, BERTScore, RadGraph/RadCliQ, GREEN-like metrics, or scores specific to individual models | Metrics do not directly measure patient safety, workflow impact, or clinical utility and were not clinically interchangeable | Use as technical context, not as primary evidence of clinical readiness |
