# Supplementary material for: Effectiveness, Safety, and Workflow Burden of Large Language Model–Based Medical Report Generation: Systematic Review
Source: J Med Internet Res. 2026 Sep 9;28:e97007. doi: 10.2196/97007 (PMC13557449; doi:10.2196/97007)
Supplement: Supplementary file 3 [file jmir-v28-e97007-s003.docx]

**Reporting Guideline Checklists**

**Contents**

| **Section** | **Navigation note** |
| --- | --- |
| PRISMA 2020 checklist | Main systematic-review reporting checklist |
| PRISMA 2020 for Abstracts | Structured abstract reporting checklist |
| PRISMA-S checklist | Search-reporting checklist updated for the May 2026 search |
| Synthesis Without Meta-Analysis (SWiM) checklist | Narrative-synthesis reporting checklist explaining why pooled meta-analysis was not performed |

**PRISMA 2020 Checklist for Systematic Review**

| **Item** | **PRISMA 2020 reporting item** | **Current manuscript location** |
| --- | --- | --- |
| 1 | Title identifies the report as a systematic review | Title |
| 2 | Abstract structured according to systematic-review reporting items | Abstract; PRISMA for Abstracts checklist |
| 3 | Rationale for the review | Introduction |
| 4 | Objectives / review question | Introduction final paragraph; Abstract Objective |
| 5 | Eligibility criteria | Methods: Eligibility Criteria |
| 6 | Information sources | Methods: Information Sources; Supplementary exact search strategies |
| 7 | Search strategy | Methods: Search Strategy; Supplementary exact search strategies |
| 8 | Selection process | Methods: Study Selection |
| 9 | Data collection process | Methods: Data Extraction |
| 10a | Data items: outcomes | Methods: Eligibility Criteria; Methods: Data Extraction |
| 10b | Data items: other variables | Methods: Data Extraction; Table 1; Supplementary extraction files |
| 11 | Risk of bias assessment | Methods: Risk of Bias Assessment; Supplementary Table S3B |
| 12 | Effect measures | Methods: Data Synthesis |
| 13a | Synthesis eligibility process | Methods: Data Synthesis; Results: Feasibility of Meta-analysis and Narrative Synthesis; Supplementary Table S7 |
| 13b | Data preparation for synthesis | Methods: Data Synthesis; Supplementary Tables S4 and S5; SWiM checklist |
| 13c | Tabulation and visual display methods | Methods: Data Synthesis; Tables 1-2; Supplementary Tables S4 and S5; Figures 2-3 |
| 13d | Synthesis methods | Methods: Data Synthesis; SWiM checklist |
| 13e | Exploration of heterogeneity | Methods: Data Synthesis; Results; Discussion: Limitations |
| 13f | Sensitivity analyses | Methods: Data Synthesis; Results: Feasibility of Meta-analysis and Narrative Synthesis |
| 14 | Reporting-bias assessment | Methods: Data Synthesis |
| 15 | Certainty assessment | Methods: Risk of Bias Assessment; Results: Risk of bias; Supplementary Table S6 |
| 16a | Study selection results | Results: Study Selection; Figure 1 |
| 16b | Excluded studies and reasons | Results: Study Selection; Supplementary full text exclusion log |
| 17 | Study characteristics | Results: Study Characteristics; Table 1 |
| 18 | Risk of bias in studies | Results: Risk of bias; Table 1; Supplementary Table S3B |
| 19 | Results of individual studies | Results: Clinically Relevant Safety and Workflow Outcomes; Table 2; Supplementary extraction files |
| 20a | Results of syntheses | Results: Clinically Relevant Safety and Workflow Outcomes; Results: Feasibility of Meta-analysis and Narrative Synthesis |
| 20b | Results of statistical syntheses | Results: Feasibility of Meta-analysis and Narrative Synthesis |
| 20c | Exploration of heterogeneity results | Results; Discussion: Limitations; Supplementary Table S7 |
| 20d | Sensitivity analysis results | Results: Feasibility of Meta-analysis and Narrative Synthesis; Supplementary Table S5 |
| 21 | Reporting biases | Methods: Data Synthesis; Discussion: Limitations |
| 22 | Certainty of evidence | Results: Risk of bias; Supplementary Table S6; Discussion |
| 23a | General interpretation | Discussion: Principal Findings; Conclusion |
| 23b | Limitations of evidence | Discussion: Limitations |
| 23c | Limitations of review process | Discussion: Limitations |
| 23d | Implications | Discussion: Clinical and Practical Implications; Conclusions and Future Directions |
| 24a | Registration information | Abstract; Methods: Protocol and Registration |
| 24b | Protocol availability | Methods: Protocol and Registration |
| 24c | Protocol amendments | Methods: Deviations From Protocol; Protocol-deviation note |
| 25 | Support and funding | Funding |
| 26 | Competing interests | Conflicts of Interest |
| 27 | Availability of data, code, and materials | Data Availability; supplementary files |

**PRISMA 2020 for Abstracts Checklist**

| **Item** | **Abstract reporting item** | **Current abstract location** |
| --- | --- | --- |
| Title | Identifies the report as a systematic review | Title |
| Objectives | States the review objective | Objective |
| Eligibility criteria | Summarizes eligible study types/interventions/tasks | Methods |
| Information sources | Names databases and search dates | Methods |
| Risk of bias | States that risk of bias was assessed and reports distribution | Methods; Results |
| Included studies | Reports number of included studies | Results |
| Synthesis of results | States that no meta-analysis was performed and explains why | Methods; Results |
| Description of effect | Reports selected clinically interpretable single study findings | Results |
| Strengths and limitations of evidence | Notes heterogeneity, bias risk, sparse safety endpoints, and lack of poolability | Results; Conclusions |
| Interpretation | Provides restrained conclusion about assistive value and lack of autonomous-readiness evidence | Conclusions |
| Funding | Funding source is in manuscript funding section rather than abstract | Funding section |
| Registration | PROSPERO 2026 CRD420261302844 | Review Registration |

**PRISMA-S Search Reporting Checklist**

| **PRISMA-S item** | **Item topic** | **How addressed in this review** | **Location** | **Status** |
| --- | --- | --- | --- | --- |
| 1 | Database name | Each bibliographic database is named individually: PubMed/MEDLINE, Embase, Web of Science Core Collection, Scopus, and the Cochrane Library. Interfaces/vendors are reported for each source. | Main Methods; Supplementary Appendix 1 search run summary | Addressed |
| 2 | Multi-database searching | Databases were searched and exported separately rather than through a single simultaneous multi-database platform. This item was therefore not part of the search methodology. | Supplementary Appendix 1 search run summary | Not applicable; explicitly reported |
| 3 | Study registries | Study registries were not used as bibliographic search sources because the eligibility criteria targeted completed peer-reviewed studies of LLM-based medical report generation. The review itself was registered in PROSPERO. | Main Methods: Information Sources; Protocol and Registration | Not searched; explicitly justified |
| 4 | Online resources and browsing | Europe PMC and OpenAlex were used as supplemental online resources to improve retrieval completeness. Their role was supplemental rather than part of the formal database total. | Main Methods: Information Sources; Supplementary Appendix 1 search documentation elements | Addressed |
| 5 | Citation searching | Backward reference screening and forward citation searching were performed for eligible studies and relevant reviews. Tools used where available included Web of Science, Scopus, and Google Scholar cited-reference features. | Main Methods: Information Sources; Supplementary Appendix 1 backward and forward citation searching sections | Addressed |
| 6 | Contacts | Corresponding authors were contacted when clinically important safety or workflow outcomes were reported in nonconvertible formats or when case-level denominator clarification was needed. | Main Methods: Information Sources; author contact log | Addressed |
| 7 | Other methods | Additional retrieval methods included Europe PMC, OpenAlex, backward reference screening, and forward citation searching. No other undocumented retrieval methods were used. | Main Methods: Information Sources; Supplementary Appendix 1 | Addressed |
| 8 | Full search strategies | Exact database-specific search strings are provided for PubMed/MEDLINE, Embase, Web of Science Core Collection, Scopus, and the Cochrane Library. Search strings are reported as run, with controlled vocabulary, field tags, Boolean operators, and exclusions where applicable. | Supplementary Appendix 1 exact strategies | Addressed |
| 9 | Limits and restrictions | Date range, English-language restriction, publication/document type limits, and exclusion of reviews/editorials/letters/protocols/conference abstracts are reported for each source where supported by the interface. | Main Methods: Eligibility Criteria and Search Strategy; Supplementary Appendix 1 database sections | Addressed |
| 10 | Search filters | No externally validated published search filter was applied. The search used investigator-built concept blocks for LLM/VLM/foundation-model terms, report generation terms, and imaging/pathology/endoscopy terms. This item was therefore not part of the methodology beyond reporting that no published filter was used. | Supplementary Appendix 1 search principles and database strings | Not used; explicitly reported |
| 11 | Prior work | The search strategy was not copied from a single prior systematic review. Existing terminology in prior reviews and eligible studies informed synonym expansion, but the final strategy was adapted for this review's broader medical report-generation scope. | Supplementary Appendix 1 search principles | Addressed |
| 12 | Updates | The original locked search was completed on March 18, 2026, and searches were updated in May 2026 before final analysis. Updated formal database exports were completed on May 14-15, 2026, and supplemental citation/reference screening continued through May 17, 2026. | Main Methods: Search Strategy; Supplementary Appendix 1 search run summary | Addressed |
| 13 | Dates of searches | Search dates are provided for each database: PubMed/MEDLINE, Embase, Web of Science Core Collection, and Cochrane Library on May 14, 2026; Scopus on May 15, 2026; supplemental searching through May 17, 2026. | Supplementary Appendix 1 search run summary | Addressed |
| 14 | Peer review | No formal PRESS or librarian peer review of the final search strategy was conducted. The strategy was iteratively checked by the review team for term coverage, database syntax, and retrieval plausibility. This is explicitly reported rather than implied. | Supplementary Appendix 1 search principles; this checklist | Not formally peer reviewed; explicitly reported |
| 15 | Total records | Records retrieved before deduplication are reported by database and for the formal database total. Updated formal database searches retrieved 5579 records before deduplication; across original and updated searches, 6936 records were identified before deduplication. | Main Methods: Search Strategy; Supplementary Appendix 1 search run summary; PRISMA flow | Addressed |
| 16 | Deduplication | Records were deduplicated using DOI, PMID, normalized title, and year. Records already represented in the original March 18, 2026 corpus were removed from the updated-screening set and counted once. | Main Methods: Search Strategy; Supplementary Appendix 1 search documentation elements; PRISMA flow | Addressed |

**Synthesis Without Meta-Analysis (SWiM) Checklist**

| **SWiM item** | **How addressed** | **Current location** |
| --- | --- | --- |
| 1. Grouping studies for synthesis | Studies are grouped by task type, clinical modality/report source, human oversight category, and outcome domain because report generation studies differ fundamentally by input, output, clinical workflow, and comparator. | Methods: Data Synthesis; Results: Study Characteristics; Table 1; Table 2 |
| 1b. Protocol changes in synthesis groupings | The protocol anticipated quantitative synthesis if compatible synthesis sets were available. The manuscript explains that synthesis remained narrative because no outcome met comparability and analyzability requirements. | Methods: Deviations From Protocol |
| 2. Standardized metric and transformation methods | No common standardized effect metric was used for pooling because outcomes included lexical metrics, semantic metrics, clinical error ratings, acceptance/preference, edit burden, reporting time, diagnostic time, and expert Likert ratings with incompatible denominators. | Methods: Data Synthesis; Supplementary Table S7 |
| 3. Synthesis methods | Structured narrative synthesis was used, prioritizing clinical, safety, human evaluation, and workflow outcomes with transparent denominators over benchmark-only language metrics. | Methods: Data Synthesis; Results; Table 2 |
| 4. Criteria for prioritizing results | Results are prioritized by clinical directness: prospective/real world workflow outcomes, blinded or multi-reader evaluation, safety or error outcomes, acceptance/preference, and workflow burden. | Methods: Data Synthesis; Table 2 selection basis |
| 5. Heterogeneity investigation | Heterogeneity is examined descriptively across modality, task type, model category, comparator/reference standard, human oversight, risk of bias, and outcome denominator. No I2 estimate is reported because no pooled estimate was calculated. | Results; Supplementary Table S7; Discussion: Limitations |
| 6. Certainty of evidence | Certainty is interpreted qualitatively using design, risk of bias, directness, outcome transparency, external validation, and availability of human/safety/workflow outcomes. | Methods: Risk of Bias Assessment; Results: Risk of bias; Supplementary Table S6 |
| 7. Data presentation methods | Study characteristics are presented in Table 1; clinically relevant human evaluation, safety, and workflow findings are presented in Table 2; nonpoolability reasons are presented in Supplementary Table S7. | Tables 1-2; Supplementary Table S7; Figures 2-3 |
| 8. Reporting synthesis results | Numerical findings are retained when they come from individual studies and are not treated as pooled estimates. Directional findings are presented by task and workflow context. | Results; Table 2; Supplementary Tables S4 and S5 |
| 9. Limitations of synthesis | The Discussion explains that narrative synthesis limits precision but is methodologically appropriate given heterogeneity, nonreconstructable denominators, clustered ratings, and inconsistent safety/workflow reporting. | Discussion: Limitations |

Note: The PRISMA-S checklist is carried forward from the current third-revision search supplement. The SWiM checklist is retained because no clinically important outcome met criteria for meta-analysis; the reasons are summarized in Supplementary Table S7.
